# Functional genomics indicate that schizophrenia may be an adult vascular-ischemic disorder

**DOI:** 10.1038/tp.2015.103

**Published:** 2015-08-11

**Authors:** H W Moises, D Wollschläger, H Binder

**Affiliations:** 1 Molecular Genetics Laboratory, Department of Psychiatry (Retired), Kiel University Hospital, Kiel, Germany; 2 Computational Genomics Lab, Frankfurt, Germany; 3 Division Biostatistics/Bioinformatics, Institute of Medical Biostatistics, Epidemiology and Informatics, University Medical Center of the Johannes Gutenberg University Mainz, Mainz, Germany

## Abstract

In search for the elusive schizophrenia pathway, candidate genes for the disorder from a discovery sample were localized within the energy-delivering and ischemia protection pathway. To test the adult vascular-ischemic (AVIH) and the competing neurodevelopmental hypothesis (NDH), functional genomic analyses of practically all available schizophrenia-associated genes from candidate gene, genome-wide association and postmortem expression studies were performed. Our results indicate a significant overrepresentation of genes involved in vascular function (*P*<0.001), vasoregulation (that is, perivascular (*P*<0.001) and shear stress (*P*<0.01), cerebral ischemia (*P*<0.001), neurodevelopment (*P*<0.001) and postischemic repair (*P*<0.001) among schizophrenia-associated genes from genetic association studies. These findings support both the NDH and the AVIH. The genes from postmortem studies showed an upregulation of vascular-ischemic genes (*P*=0.020) combined with downregulated synaptic (*P*=0.005) genes, and ND/repair (*P*=0.003) genes. Evidence for the AVIH and the NDH is critically discussed. We conclude that schizophrenia is probably a mild adult vascular-ischemic and postischemic repair disorder. Adult postischemic repair involves ND genes for adult neurogenesis, synaptic plasticity, glutamate and increased long-term potentiation of excitatory neurotransmission (i-LTP). Schizophrenia might be caused by the cerebral analog of microvascular angina.

## Introduction

Schizophrenia is characterized by cognitive deficits, hallucinations, delusions and a heterogeneous, sometimes deteriorating clinical course.^1^ Brain imaging studies show neuronal processing abnormalities^2^ and a progressive decline of brain volume affecting both white and gray matter.^3, 4^ Although it has a strong genetic component heritability estimates (64–90%),^5, 6, 7^ onset and relapse are associated with environmental factors.^7, 8^

Since the first complete genome scan with significant results by Moises *et al.*^9^ identified several chromosomal loci for genes predisposing individuals to schizophrenia, an ever-increasing number of genes have been implicated in the disorder. To facilitate the interpretation of the findings from over 1700 genetic association studies, Bertram *et al.* created a publicly accessible database^10^ hosted by the Schizophrenia Research Forum (http://www.szgene.org). In addition, the National Human Genome Research Institute provides a curated resource of single-nucleotide polymorphism–trait associations (GWAS catalog)^11^ (http://www.genome.gov/gwastudies/). The function of this diverse set of genes in the pathogenesis of the disorder still remains elusive. Recently, Sullivan^12^ proposed that the genetic risk for schizophrenia operates at the pathway level and that the identification of a common pathway for candidate genes would help to elucidate the etiology of this complex disorder.

The aim of the present study was to identify schizophrenia pathways. The investigation was started in August 2010 at a time when ZNF804A was the only replicated gene from genome-wide association studies (GWASs).^13, 14^ Therefore first, candidate genes were selected based on the criterion of independent replication. In 2014, shortly before submission of the manuscript, an impressive large-scale GWAS by the Schizophrenia Working Group of the Psychiatric Genomics Consortium (SWGPGC) was published,^15^ which prompted us to test our results from candidate gene studies in samples of genes obtained from GWASs.

## Materials and methods

Family-based methods, such as the transmission disequilibrium test (TDT), are thought to avoid false-positive results caused by stratification problems of case–control designs. For this reason, a sample of 33 candidate genes from TDT studies identified by two independent research groups was employed as a discovery sample. The discovery sample is described in more detail in Supplementary Table S1. Four replication samples were analyzed.

The first replication sample was derived from case–control studies and consisted of 58 candidate genes found by at least three independent research groups (details are given in Supplementary Table S2). The second replication sample comprised 164 genes from the National Human Genome Research Institute catalog of GWASs (http://www.genome.gov/gwastudies) (for details, see Supplementary Table S3). The third replication sample consisted of 42 genes carefully selected from GWASs by Ayalew *et al.*^16^ using a convergent functional approach^16^ (see Supplementary Table S4). A fourth replication sample comprised 111 genes assigned by proximity to the 108 genome-wide significant regions of the SWGPGC's recent large GWAS^15^ (for rules of assignment, see Supplementary Table S5). In addition, to exclude a bias introduced by gene assignment, the entire list of 343 genes within range of the genome-wide significant loci, as published by the SWGPGC, was investigated in a separate analysis.

To gain an overall picture, the updated TDT sample and the four replication samples were combined in which the fourth replication sample was conservatively represented only by the 111 proximity-assigned genes. Finally, a comprehensive analysis of a large combined cohort from seven independent postmortem gene expression studies of the prefrontal cortex of schizophrenic patients by Mistry *et al.*^17^ provided samples of 113 differentially expressed, 31 upregulated and 82 downregulated genes. For background, 13 176 unique genes were employed as indicated by Mistry *et al.* (personal communication). The list of the seven postmortem studies is given in Table 1 of the original publication.^17^

### Construction of a candidate schizophrenia pathway

The first step was to simplify the puzzle by focusing on a reduced number of more reliable findings (that is, candidate genes from TDT studies as a discovery sample). Second, using this discovery sample, an attempt was made to identify a candidate pathway by protein–protein interactions and pathway analyses. For the former, STRING was employed, and for the latter KEGG, GO, PANTHER and DAVID (as requested by an anonymous reviewer; references and results of the analyses are shown in Supplementary Figures S2–S5 and Supplementary Tables S10, S12–S14).

Third, a manual approach was used for generating a candidate pathway from the discovery sample. The main problem for the construction of a pathway based on all 33 genes of the discovery sample was to find a common denominator for neurotransmitter-related and vasoregulatory genes (for example, PLA2G4A and NOS1AP). Both groups of genes converge on blood vessels and are involved in the regulation of cerebral blood flow (CBF). Neurotransmitter-related genes, as part of perivascular nerves and vasoregulatory genes, might be responsible for the impaired vasodilatation of schizophrenic patients, revealed by the niacin flush test.^20, 21^ Subsequently, results from the protein interaction analysis by STRING (Supplementary Figure S2), the pathway analyses by KEGG, GO, PANTHER and DAVID (Supplementary Tables S12–S14 and Supplementary Figures S2–S5) and the focused literature searches (Supplementary Table S11) were used to localize the genes of the discovery sample within neurovascular-coupling structures.^22, 23^ The involvement of NOS1AP and NOS1 in schizophrenia has recently been confirmed by meta-analysis.^24^

However, the development of an energy-supply-based candidate pathway for schizophrenia does not prove the correctness of our interpretation of the data. Similar lists of candidate genes for schizophrenia have been used by others to develop synaptic (SY) or neurodevelopmental (ND) models. According to the recommendation by Cantor *et al.*,^25^ a candidate pathway must be formally tested by statistical methods in independent replication samples not previously employed for the identification of the candidate.

### Functional genomic analysis

A functional gene set approach was adopted for statistical analysis. The functional gene sets were obtained by extensive literature mining. The authors gratefully acknowledge that the idea to build databases for functional gene sets from the literature was inspired by the prior work of Schmidt-Kastner *et al.*^26^

The functional gene sets consisted of 3500 vascular genes (V), 2866 genes induced by acute cerebral ischemia or reperfusion (I), 159 genes involved in postischemic repair (R), 4020 genes differentially expressed during ND, and 2988 SY genes. For more details, see Supplementary Tables S6–S9. To test for interactions between V, I, VI and ND genes, samples of overlapping genes were created. The same was done by substituting ‘Repair' genes (R and ND) for ND (see the Venn diagram in Supplementary Figure S1).

### Statistical analyses

The number of intersections between schizophrenia-associated genes and functional gene sets was determined by the intersect function implemented in R.^27^ Statistical significance and confidence intervals for the number of matches between schizophrenia-associated genes and each functional gene set were determined using separate Fisher's exact tests (one sided) as implemented in R.^27^ The validity of this statistical approach was verified by a custom genome resampling test (described in the Supplementary Information) that led to the same results. The level of significance was set at 0.01 after application of the conservative Bonferroni correction for multiple testing.

## Results

### Candidate schizophrenia pathway

**Figure 1:**
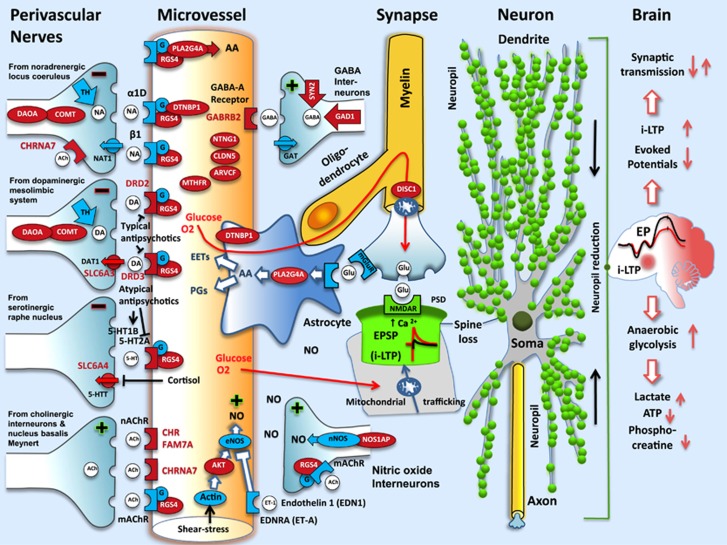
Localisations of candidate genes for schizophrenia from TDT studies within the energy-delivering pathway for synapses (adapted from Nave,^22^ Hamel^28^ and Drake and Iadecola^23^). The pathway encompasses perivascular nerves, microvessels, astrocytes, oligodendrocytes, myelin and mitochondrial trafficking. Candidate genes are marked in red. Vasodilatation or vasoconstriction is indicated by + or − symbols, respectively. Noradrenaline, dopamine and serotonin have a vasoconstrictory effect, whereas acetylcholine, GABA, glutamate, nitric oxide, shear stress and growth factors appear to promote vasodilatation. Mild ischemia induces reversible loss of dendritic spines and structure,^29^ impaired synaptic transmission (indicated by decreased amplitude of evoked potentials), anaerobic glycolysis and reduction of high-energy phosphates, such as ATP, phosphocreatine and i-LTP (reviewed in Di Filippo^30^and Heiss^31^). EP, evoked potentials; EPSP, excitatory postsynaptic potential; i-LTP, postischemic long-term potentiation; NO, nitric oxide; PSD, postsynaptic density; TDT, transmission disequilibrium test. References for localisations of genes are given in Supplementary Table S11.

**Figure 2:**
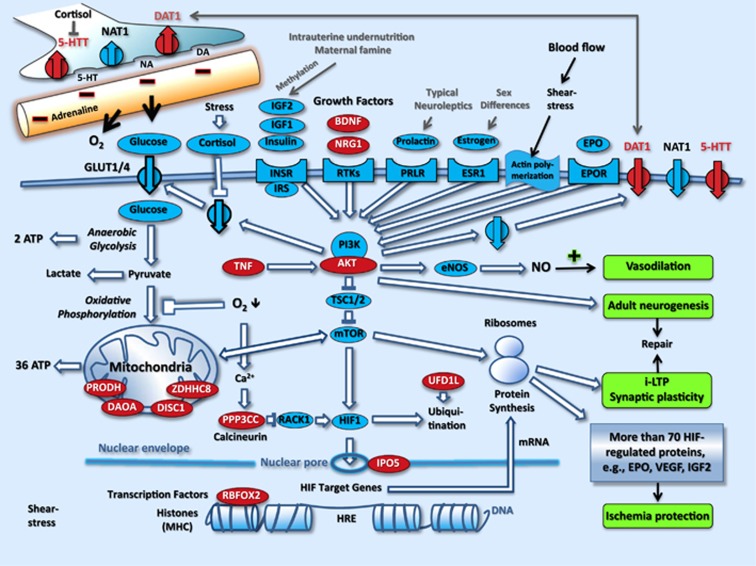
The cellular localisations of candidate genes for schizophrenia from TDT studies indicate a role in energy production and protection from ischemia. On the basis of information from databases (KEGG, GO and STRING) and the literature (see Supplementary Figures S2–S5 and Supplementary Tables S11–S14), Akt regulates endothelial NO production^32^ and expression of transporters (circles with an arrow) for glucose, dopamine, noradrenaline and serotonin at the cell surface (reviewed in González and Robinson^33^). PI3K/Akt is of central importance to the signal transduction of hormones and growth factors in blood flow, metabolism, ischemia protection and postischemic repair by adult neurogenesis^34^ and by synaptic plasticity (i-LTP).^30, 35^ HIF1, hypoxia-inducible factor 1; HRE, HIF-responsive element; MHC, major histocompatibility complex; NO, nitric oxide; ROS, radical oxygen species; RTK, receptor tyrosine kinase; TDT, transmission disequilibrium test; TNF, tumor necrosis factor. References for gene localisations are given in Supplementary Table S11.

The candidate pathway for schizophrenia illustrates that the majority of genes of the discovery sample can be localized within the intercellular (Figure 1) and intracellular (Figure 2) pathways for energy supply to synapses and protection from ischemia.

### Functional genomic analysis

**Figure 3:**
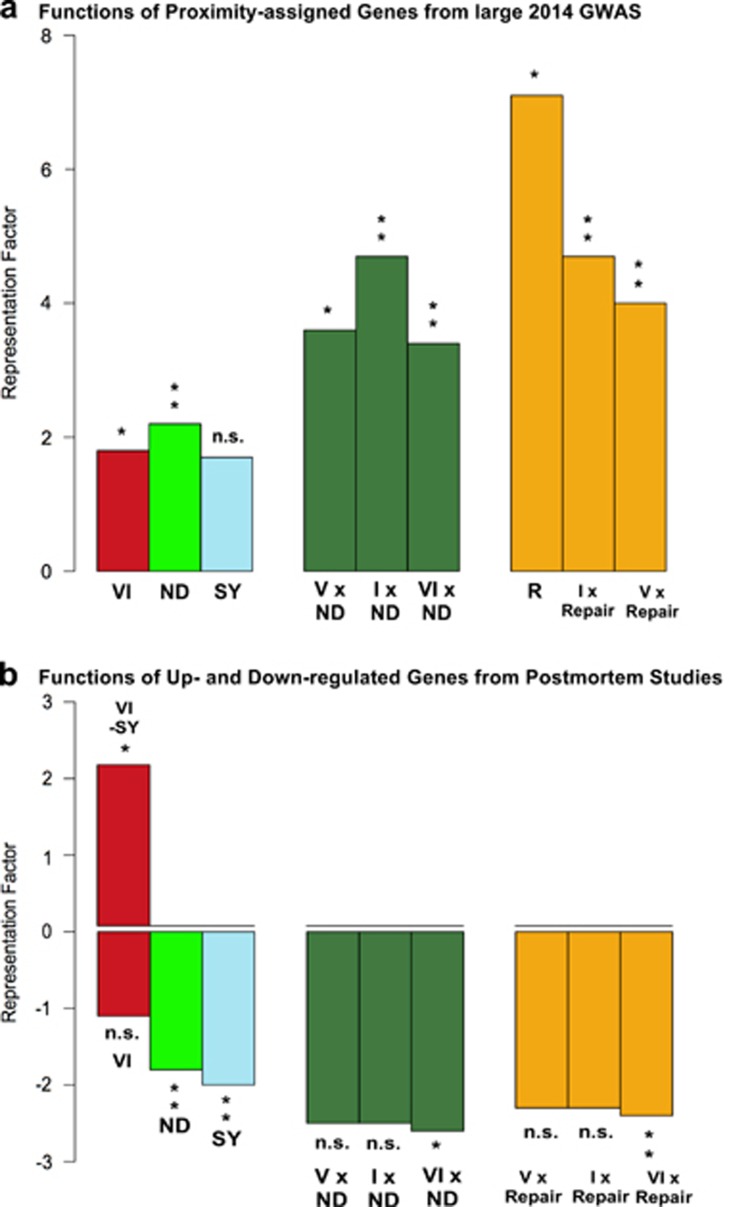
Functions of schizophrenia-associated genes. The results for genes from the large 2014 GWAS^15^ and from postmortem studies^17^ are depicted in **a** and **b**, respectively. (**a**) Vascular (V), ischemic (I), neurodevelopmental (ND) (which are also active during postischemic repair^18, 19^) and postischemic repair (R) genes appear to have a significant role in the genetic predisposition to schizophrenia. ‘Repair', ND and R genes combined. (**b**) The postmortem profile of upregulated vascular-ischemic genes (VI-SY) and downregulated ND and SY genes in the adult brain of schizophrenic patients is compatible with cerebral ischemia. GWAS, genome-wide association study; ND, neurodevelopmental genes; n.s., not significant. Level of significance (Bonferroni corrected, see Supplementary Tables S20 and S21): (**a**) *P⩽0.01; **P⩽0.001; (**b**) *P⩽0.05; **P⩽0.01.

The results of the functional genomic analyses of the discovery and the four replications samples are given in Supplementary Tables S15–S20, whereas the results of the combined sample are provided in Table 1. Concerning the important large GWAS by the SWGPGC (2014), the results of the analysis of all the 343 genes within range of the significant loci are shown in Supplementary Table S19, whereas those of the 111 genes assigned by proximity to genome-wide significant index single-nucleotide polymorphisms are provided in Supplementary Table S20 and Figure 3a.

The results of the analysis of differentially expressed genes from postmortem studies are depicted in Supplementary Table S21 and Figure 3b. For the upregulated genes, only the significant result is shown in Figure 3b. An overview of the significant findings from the functional genomic analysis is given in Supplementary Table S22.

### Quasi-experimental study

The data and references for the quasi-experimental disturbance of the candidate pathway are listed in Supplementary Table S23, and those for ND disturbance in Supplementary Table S24. A summary of these data is depicted in Supplementary Figure S6.

## Discussion

The search for the elusive candidate pathway for schizophrenia was conducted in three steps. First, a candidate pathway was developed (Figures 1 and 2). Second, to test the pathway, functional genomic analyses of practically all available schizophrenia-associated genes from candidate gene, GWA and postmortem expression studies were employed (Figure 3 and Supplementary Table S22). Third, a quasi-experimental approach was used to recheck the individual components of the candidate pathway (Supplementary Figure S6 and Supplementary Tables S23–S24).

With regard to perivascular nerves, the candidate pathway demonstrates that neurotransmitter-related candidate genes for schizophrenia not only support a SY hypothesis, but also a vascular-ischemia model (see Figure 1). In addition, several factors involved in schizophrenia (such as prenatal risk and growth factors, sex differences, stress and antipsychotics) exert an influence on vasoconstriction via the PI3K/AKT-mediated reuptake of serotonin, norepinephrine and dopamine. Chronic abuse of the dopamine reuptake inhibitor amphetamine is known to cause vasoconstriction, cardiovascular and cerebrovascular ischemia, and a clinical picture indistinguishable from schizophrenia. Furthermore, a role for blood-flow regulation in schizophrenia is suggested by the influence of the PI3K/AKT pathway on the production of nitric oxide via actin and eNOS (Figures 1 and 2). Taken together, the candidate pathway depicted in Figures 1 and 2 suggests that schizophrenia might be a mild adult vascular-ischemic disorder.

Next, to test the adult vascular-ischemia hypothesis (AVIH), functional genomic analyses of all available schizophrenia-associated genes from candidate gene, GWA and postmortem studies were carried out. The results clearly show in all samples a significant overrepresentation of a combined gene set comprising all gene sets involved in cerebral ischemia (that is, VI, R and ND) (see overview in Supplementary Table S22). Nearly identical results are obtained by the individual or interacting gene sets (see Figure 3a; Table 1; Supplementary Tables S15–S20). Only the SY gene set stands out by its lack of significant overrepresentation in the important large GWAS by the SWGPGC (2014)^15^ (see Figure 3a).

ND genes have two functions. They are important for neurodevelopment and for postischemic repair.^18, 19^ Therefore, the overrepresentation of ND genes and of VI × ND interacting genes does not automatically provide proof for the ND hypothesis (NDH) of schizophrenia. An alternative explanation has to be considered, as the possibility exists that these results might indicate a role for genetically impaired postischemic repair in the pathogenesis and course of schizophrenia, a major psychosis defined by its chronic course by Kraepelin.

This interpretation is supported by the significant overrepresentation of the functional gene set consisting of postischemic repair genes (R) in five of six schizophrenia-associated gene samples (see Supplementary Tables S15–S20 and S22). As ND genes also have an important role in postischemic repair,^18, 19^ the two gene sets involved in postischemic repair were combined (R and ND) for analysis and termed ‘Repair'. In all samples from association studies, the Repair gene set resulted in an increased overrepresentation compared with the ND genes alone. The higher representation factor suggests, but does not prove, that the repair function of ND genes might be more important than their ND function in the pathogenesis and course of schizophrenia (see Figure 3a; Table 1; Supplementary Tables S15–S20). The interaction of VI and Repair genes is likely to be of importance for the outcome of mild ischemia in the fetal and the adult brain. Therefore, our findings are consistent with both the NDH and the AVIH.

The results from postmortem studies, however, do not support a purely ND model (see Figure 3b). In adult schizophrenic patients (mean age 55.27±19 years^17^), VI genes (VI-SY) were upregulated and SY and ND genes were downregulated (Figure 3b), a finding consistent with cerebral ischemia. To test this interpretation, lists of downregulated genes were compiled from the ischemia-induced gene set (I) and employed for intersection analysis. The results indicate that of the genes downregulated by cerebral ischemia (*N*=476), 43% were ND and 43.7% SY genes (both *P*⩽2.2E−16).

To further test the AVIH vs the NDH of schizophrenia, data from the literature were compiled for a quasi-experimental investigation (Supplementary Tables S23–S24). Again, the results favor the AVIH by showing that severe neurodevelopmental impairment in very preterm infants does seem to have little effect on the rate of schizophrenia (see Supplementary Tables S23–S24 and Supplementary Figure S6).

Importantly, the results of our study are in agreement with the findings of other researchers.

Concerning pathway analyses, Schmidt-Kastner *et al.*^26, 36^ have previously tested candidate genes for schizophrenia for overlap with VI genes from the brain and were the first to report a high percentage of overlap in 2006 and again in 2012. Our VI results independently replicated and confirmed their findings. The main difference was in our analysis of additional gene sets (that is, ND, SY, R and postmortem samples) and in the conclusion. Assuming the correctness of the NDH, Schmidt-Kastner *et al.*^26, 36^ interpreted their results as a confirmation of this hypothesis. The present study, aimed at testing the AVIH, differed in several ways: material from the fetal brain was excluded, neurodevelopmental, repair, and SY genes were included and extensive statistical testing was applied in an attempt to differentiate between the AVIH and the NDH. However, our findings based on genetic association studies equally support both the NDH and the AVIH.

In addition, the results of pathway analyses by other researchers do not conflict with the AVIH. Evidence for gene enrichment has been found in pathways for actin remodeling,^37^ glutamate metabolism,^15, 38^ voltage-sensitive calcium channels,^15^ myelin,^39^ immune system,^15, 40^ inflammation,^41^ lysosomal function,^37^ adult neurogenesis^41^ and SY plasticity.^15^ All these divergent results have in common that they have a role in either ischemia or postischemic repair.

The ischemia hypothesis of schizophrenia has a long tradition largely forgotten or neglected by contemporary researchers. In 1890, Meynert,^42^ Freud's professor in Vienna, speculated in his influential textbook on psychiatry about the possibility that mania (a term for acute psychosis at that time) might be caused by reduced CBF and depression by an increase in functional hyperemia.

The results of our functional genomic analyses are in agreement with important postmortem findings in schizophrenia indicating myelin dysfunction (reviewed in Davis *et al.*^43^and Nave and Ehrenreich^44^), which might be due to the selective ischemic vulnerability of myelin-producing oligodendrocytes,^45^ further with mitochondrial dysfunction and impaired energy metabolism^17, 46, 47, 48, 49^ and a reduction of dendritic spines and SY proteins (reviewed in Falkai *et al.*^50^and Harrison *et al.*^51^). The latter may also stem from mild cerebral ischemia^29^ (see Figure 1).

Vascular factors in schizophrenia were already investigated early on. For example, Cotton *et al.*^52^ reported in 1940 an inverse relationship between the size of the retinal vascular bed and a progressive course in schizophrenia, and Senitz and Winkelmann^53^ simplified angioarchitecture and abnormal arborization of brain vessels. Ten years ago using stereological methods, Kreczmanski *et al.*^54, 55^ ruled out alterations in microvessel length density, total microvessel length and microvessel length per neuron in the adult brain afflicted by schizophrenia. Kreczmanski *et al.* proposed that compromised brain metabolism and occurrence of oxidative stress in the brains from patients with schizophrenia^48^ are likely caused by other mechanisms such as functional disruption in the coupling of CBF to neuronal metabolic needs.^55^ Evidence for vasoregulatory disturbances was also recently found by Meier *et al.*^56^ in a population-representative birth cohort. These authors reported wider retinal venules in schizophrenic patients that might be caused by endothelial dysfunction or hypoxia/ischemia. Electron microscopical evidence for cerebral ischemia or chronic hypoperfusion was obtained by Uranova *et al.*^57^ from the capillaries of the neocortex.

Furthermore, actin has an important role in the regulation of blood flow^58^ (see Figures 1 and 2 and Supplementary Figure S5) and in schizophrenia as revealed by exome sequencing^59^ and pathway analysis.^37^ In addition, all effective treatments in schizophrenia converge on an improvement of cerebral perfusion and/or protection against ischemia (see Supplementary Table S29).

Vascular inflammation as a cause of schizophrenia has been postulated by Hanson and Gottesman.^60^ Undoubtedly, vascular inflammation is able to cause a schizophrenia-like syndrome as demonstrated by lupus erythematosus (see Supplementary Figure S6, reference in Supplementary Table S23). Moreover, ischemia is known to induce inflammation. The results presented here suggest that the focus on only one factor might be too narrow. Other factors are also able to induce mild cerebral ischemia and schizophrenia-like symptoms (see Figure 1 and Supplementary Figure S6).

Concerning postischemic repair, our findings indicate a significant role for repair genes in the predisposition to schizophrenia (Figure 3a; Table 1). Evidence for the involvement of repair mechanisms in schizophrenia has been obtained by several groups (see Supplementary Table S30). Most importantly, Reif *et al.* found that the neural stem cell proliferation required for adult neurogenesis is reduced in schizophrenia, but not in major depression.^61^ The PI3K/Akt pathway is involved in schizophrenia and mediates the effects of stress, hormones and growth factors on SY plasticity and adult neurogenesis (that is, on neuronal and SY repair)^34, 35^ (see Figure 2).

Taken together, the AVIH is supported by additional evidence in the literature for mild cerebral ischemia, functional vasoregulatory disturbances and reduced repair capacity in schizophrenic patients.

With regard to disease concepts, our findings based on genetic association studies can be interpreted as support for the NDH, as well as for the AVIH. It can reasonably be argued, as Schmidt-Kastner *et al.* assumed, that ischemia genes expressed in the adult brain are probably also prenatally activated in the developing brain exposed to ischemia^36^ and vice versa. Thus, the difference is not in the results, but in their interpretation.

The NDH is able to explain a large number of facts (see Supplementary Table S27, left column, and Supplementary Figure S7) and is so widely accepted that any different interpretation is confronted with opposition. However, we cannot avoid the discussion of the alternative AVIH because of its important translational consequences. The NDH has been criticized for implying an inevitability about the development of schizophrenia, a therapeutic nihilism in which patients with schizophrenia are ‘doomed from the womb'.^62^ The AVIH, on the other hand, implies that the monitoring of CBF, EEG and blood for signs of ischemia in patients or individuals at risk should improve treatment and outcome of the disease.

**Figure 4:**
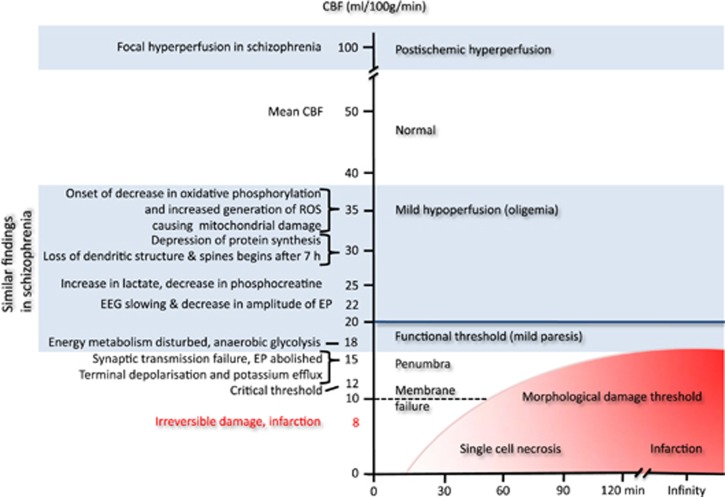
Threshold model of cerebral blood flow. Adapted from Heiss.^63^ The red shaded area indicates infarction and the area in light blue indicates similar findings reported in schizophrenia (Supplementary Table S26 for references). CBF, cerebral blood flow; EEG, electroencephalography; EP, evoked potentials; ROS, reactive oxygen species.

Hypotheses can be tested by their predictions. The NDH predicts evidence for prenatal damage in the brains of schizophrenic patients, whereas the AVIH postulates signs of cerebral ischemia during acute psychosis. With regard to the AVIH, the consequences of mild cerebral ischemia have been well documented by stroke researchers in their CBF threshold (penumbra) model (Figure 4). Similar findings were reported in adult schizophrenic patients during psychosis (see Figure 4 and Supplementary Table S25 for references). This relates schizophrenia to the CBF threshold model as a plausible pathogenic mechanism. Furthermore, signs of cerebral ischemia can be found in schizophrenic patients on the biochemical, cellular, electroencephalographic, brain imaging and clinical levels (see Figure 3b and Supplementary Table S26).

By comparison, the prediction of the NDH is not supported by neuropathological evidence. According to critical reviews by Paul Harrison, one of the leading neuropathologists in this field, cytoarchitectural abnormalities occurring during prenatal neurodevelopment have never been unequivocally established in schizophrenia and the established neuropathological findings could well originate much later in life.^51, 64^ Furthermore, the overall majority of schizophrenic patients (93%) were never exposed to (broadly defined) birth complications that might cause a disturbance in brain development (see meta-analysis of prospective population-based studies by Cannon *et al.* (Supplementary reference 500). The difference in the normal population is only 1.6% (see Supplementary Table S28), hardly convincing evidence for ND disturbances as a cause of schizophrenia. In addition, the superior premorbid intelligence (IQ) of a large number of schizophrenic patients^65, 66, 67, 68^ is at variance with the NDH (see Supplementary Information for a global projection of high-IQ individuals affected by schizophrenia). Such a large number of individuals with normal or superior intelligence are in contradiction to the postulated neurodevelopmental brain defect. Further anomalies of the NDH are listed in Supplementary Table S31 and include progressive brain tissue loss,^3, 4^ remissions, relapses, progression^1^ and treatment-dependent outcomes.^62, 69^

To save the prevailing NDH, it might be argued that it does not exclude the possibility of minor hypoperfusion during psychosis. The all-inclusive argument begs the question of whether the assumption of early brain damage caused during neurodevelopment is really necessary to explain the available evidence.

The NDH is built on epidemiological studies demonstrating that prenatal factors and a slight maturational delay in childhood are associated with an increased risk for schizophrenia (reviewed in Harrison,^70^ Moises *et al.,*^71^ Marenco and Weinberger^72^ and Weinberger and Levitt^73^). The same two factors have repeatedly been demonstrated to be associated with adult vascular disorders, also known as Barker's theory of the fetal origins of adult vascular disorder.^74, 75, 76^ As an explanation, Barker offered phenotypic plasticity (that is an evolutionary mechanism to adapt an individual to its environment),^75^ whereas ND theorists posited brain damage (sometimes described as a miswired brain) inflicted during neurodevelopment (reviewed in Weinberger and Levitt^73^).

The evidence in support of the NDH and the alternative AVIH is listed in Supplementary Table S27 and visually summarized in Supplementary Figure S7. These studies place schizophrenia among several adult vascular disorders such as angina, myocardial infarction and stroke (see Supplementary Figure S7). Adult vascular disorder as an intermediary variable between prenatal risk factors, maturational delay, and schizophrenia has previously been ignored. The Barker theory about the fetal origin of adult vascular disorders^74, 75, 76^ appears to serve as an equivalent substitute for the NDH for explaining prenatal risk factors and maturational delays in schizophrenia (see Supplementary Figure S7).

On the other hand, the AVIH is supported by several lines of evidence. First, the results of our functional genomics analyses, as reported here. Second, the alternative explanations for the facts of the NDH, as listed in Supplementary Table S27 (second column). Third, Barker's epidemiological findings putting schizophrenia among adult vascular disorders (Supplementary Figure S7). Fourth, the agreement with the well-established dopamine hypothesis of antipsychotic drug action (Figure 1). Fifth, the ability to explain the signs of ischemia during acute psychosis (Supplementary Table S26). Sixth, the effect of different treatments on CBF and ischemia (Supplementary Table S29). Seventh, the higher explanatory power of the AVIH as compared with the NDH (Supplementary Table S31).

By comparison, the ND brain damage hypothesis is at variance with our postmortem results (Figure 3b) and those of others,^48, 51, 64^ with several essential facts of schizophrenia convincingly demonstrated by Lieberman^62^ (Supplementary Table S31), including the following: the majority of patients neither experienced birth complications (broadly defined) nor showed any minor physical anomalies (Supplementary Tables S28 and S31), soft neurological signs decrease with the remission of acute psychosis and are nearly as prevalent in normal individuals as in patients (Supplementary Tables S25 and S27), and finally, the superior premorbid intelligence of a large number of patients,^65, 66, 67, 68^ including Noble prize winner John Forbes Nash Jr and his son, who was also a gifted mathematician and a top chess player before the illness struck.^77^

A major problem of the AVIH is the delay between the onset of prodromal or outpost symptoms and the onset of psychotic symptoms.^65, 78, 79^ The interval is ~2–5 years for schizophrenia.^79^ It appears plausible to assume that prodromal and negative symptoms are signs of mild cerebral ischemia.^80, 81, 82^ But then, what causes the positive symptoms of acute psychosis? Postischemic long-term potentiation of excitatory synaptic transmission (i-LTP) appears to be a plausible mechanism.

The term i-LTP refers to a postischemic repair mechanism in the neighborhood of ischemic brain areas by which neuronal networks that are normally not involved in the function of the ischemic area are progressively activated (reviewed in Di Filippo *et al.*^30^). This process takes weeks or even months.^83^ In schizophrenia, increased activation of neuronal networks has been found to be associated with positive symptoms such as auditory verbal hallucinations, delusion of control, inner speech, formal thought disorder and grandiosity^80, 84, 85, 86^ (reviewed in Meyer-Lindenberg and Bullmore,^2^ meta-analysis by Jardi *et al.*^84^). In i-LTP, long-term potentiation of the glutamate-mediated excitatory postsynaptic potential is enhanced by SY plasticity.^30^ Genes for glutamate metabolism and SY plasticity have been found to be associated with schizophrenia.^15, 24, 38, 59, 87^ Multiple episodes of mild cerebral ischemia may cause a perpetual increase of excitatory postsynaptic potential, i-LTP, neuronal excitability and subsequent hyperactivity of cortical areas involved in positive symptoms. Mild ischemia might be responsible for the negative and i-LTP for the positive symptoms in schizophrenia.

We should emphasize, quasi as atonement for our critical review of the widely accepted NDH, that the latter ‘is not a hypothesis in terms of it being falsifiable, rather it is a model or framework for understanding diverse aspects of the pathogenesis'.^88^ Furthermore, it is possible that the first episodes of mild cerebral ischemia might already begin *in utero* or during postnatal neurodevelopment, and that adult ischemia or i-LTP could function as the second hit required by the NDH to explain the adult onset of schizophrenia.

Stress-induced vasoconstriction (Figure 2), in combination with a genetically and/or epigenetically restricted energy-delivering pathway, and impaired vasodilatation (Figures 1 and 2) might be one of the most frequent causes of mild ischemia in schizophrenia, considering the extreme introvert personality of the majority of patients,^89^ the high prevalence of depression and anxiety in first psychotic episodes (as well as in relapses)^90^ and the negative impact of these factors on CBF.^91, 92, 93^

Concerning translational aspects, there is reason for cautious optimism.^94^ Several techniques are available to monitor psychiatric patients for signs of mild cerebral ischemia and to evaluate the effects of treatments on CBF and oligemia. In analogy to cardiology, stress tests may be applied in psychiatry to diagnose an increased risk for ischemia and schizophrenia. In principle, it might even be possible to prevent schizophrenia by treating mild ischemia in the prodromal phase, or at least to reduce its harmful effects on brain tissue and the progression of the disease.

In summary, although the NDH cannot be excluded, the main criteria for theory evaluation, that is, consilience, simplicity and analogy,^95^ suggest that the AVIH currently provides a better explanation for the facts of schizophrenia (Supplementary Table S31). We conclude that schizophrenia is probably a mild adult vascular-ischemic and postischemic disorder of the brain. Evidently, more research is needed to address the interaction of vascular, ischemic, neurodevelopmental and repair factors in major psychoses. From a wider perspective, schizophrenia might be caused by the cerebral analog of angina with normal coronary arteries, variously termed Prinzmetal's, variant, vasospastic or microvascular angina.^96^

## Figures and Tables

**Table 1:** Intersection of functional gene sets with schizophrenia-associated genes from all genetic association studies combined (duplicate genes removed)

*Functions*	*N*	*SZ N*	*E (*N)	*O (*N)	*E (%)*	*O (%)*	*RF*	*CI*	*Nominal* P	*Bonferroni corrected* P
VIRND	6409	345	105	195	30.5	56.5	1.9	179–345	2.2E−16	5.1e−15***
VI	4213	345	69	139	20.1	40.3	2.0	124–345	2.2E−16	5.1e−15***
V	3249	345	53	110	15.5	31.9	2.1	96–345	2.2E−14	5.1e−13***
PV	253	345	4	35	1.2	10.1	8.4	26–345	2.2E−16	5.1e−15***
V−PV	2996	345	49	75	14.2	21.7	1.5	62–345	1.1E−04	2.5e−03*
SS	2818	345	46	69	13.4	20.0	1.5	60–345	4.3E−04	9.9e−03*
I	1673	345	27	74	7.9	21.5	2.7	61–345	4.8E−15	1.1e−13***
R	159	345	3	31	0.8	9.0	11.9	23–345	2.2E−16	5.1e−15***
Repair	3319	345	54	129	15.8	37.4	2.4	114–345	2.2E−16	5.1e−15***
ND	3211	345	53	111	15.3	32.2	2.1	97–345	3.8E−15	8.7e−14***
VI × ND	1050	345	17	57	5.0	16.5	3.3	46–345	8.8E−16	2.0e−14***
VI × Repair	1123	345	18	73	5.3	21.2	4.0	61–345	2.2E−16	5.1e−15***
V × ND	783	345	13	44	3.7	12.8	3.4	34–345	7.6E−13	1.8e−11***
V × Repair	844	345	14	59	4.0	17.1	4.3	48–345	2.2E−16	5.1e−15***
I × ND	522	345	9	37	2.5	10.7	4.3	28–345	6.1E−14	1.4e−12***
I × Repair	568	345	9	47	2.7	13.6	5.0	37–345	2.2E−16	5.1e−15***
SY	1977	345	32	91	9.4	26.4	2.8	77–345	2.2E−16	5.1e−15***
VI−ND	3163	345	52	82	15.4	23.8	1.6	69–345	1.4E−05	3.2e−04**
VI−SY	3273	345	54	76	15.6	22.0	1.4	63–345	9.8E−04	2.3e−02 NS
I−ND	1151	345	19	37	5.5	10.7	2.0	28–345	8.9E−05	2.1e−03*
I−SY	1180	345	19	38	5.6	11.0	2.0	28–345	6.9E−05	1.6e−03*
ND−VI	2161	345	36	54	10.3	15.7	1.5	43–345	1.3E−03	3.0e−02 NS
SY−VI	1037	345	17	28	4.9	8.1	1.6	20–345	7.5E−03	1.7e−01 NS

Abbreviations: CI, 95 percent confidence intervals expressed as the number of identical genes; E, expected number and percentage of intersecting genes by chance; I, ischemia-induced cerebral genes; minus sign (−), overlapping genes removed, for example, V−PV, V without PV; ND, neurodevelopmental genes; NS, not significant; O, observed number or percentage of intersecting genes; PV, perivascular genes; R, postischemic repair genes; Repair, R and ND genes combined, because ND genes are involved in postischemic repair;^18, 19^ RF, representation factor, that is, the number of intersecting genes divided by the expected number of intersecting genes under independence assumption; SS, shear stress-induced endothelial genes; SY, synaptic genes; SZ, schizophrenia-associated genes; V, vascular genes including perivascular genes; VI, vascular and ischemia genes; VIRND, vascular, ischemia and postischemic repair genes.

×, gene interaction, for example, VI × ND, interaction of VI and ND genes.

Level of significance (Bonferroni corrected): *P⩽1.0E−02; **P⩽1.0E−03; ***P⩽1.0E−06.
