# Supplementary material for: Functional genomics indicate that schizophrenia may be an adult vascular-ischemic disorder
Source: Transl Psychiatry. 2015 Aug 11;5(8):e616–. doi: 10.1038/tp.2015.103 (PMC4564558; doi:10.1038/tp.2015.103)
Supplement: Supplementary Information [file tp2015103x1.pdf]

Functional genomics indicate that schizophrenia may be an adult vascular-ischemic disorder

Hans Moises, Daniel Wollschläger, and Harald Binder

Supplementary Information

Table of Contents

SUPPLEMENTARY TABLES 3
SUPPLEMENTARY FIGURES 4

MATERIAL 5

CANDIDATE GENES FOR SCHIZOPHRENIA 5
TABLE S1. DISCOVERY SAMPLE BASED ON TDT-STUDIES 5
TABLE S2. REPLICATION SAMPLE #1 BASED ON CASE-CONTROL STUDIES 5
GENOME-WIDE ASSOCIATION STUDIES (GWAS) 6
TABLE S3. REPLICATION SAMPLE #2 OBTAINED FROM THE NHGRI CATALOG 6
TABLE S4. REPLICATION SAMPLE #3 OBTAINED FROM AYALEW ET AL. (2012) 6
TABLE S5. REPLICATION SAMPLE # 4 OBTAINED FROM SWGPGC 2014 7
FUNCTIONAL GENE SETS 7
TABLE S6. VASCULAR AND ACUTE ISCHEMIA GENES OF THE ADULT BRAIN 7
TABLE S7. NEURODEVELOPMENTAL GENES 7
TABLE S8. SYNAPTIC GENES 7
TABLE S9. POST-ISCHEMIC REPAIR GENES 8
FIGURE S1. VENN DIAGRAMS 8

METHODS 9

CONSTRUCTION OF SCHIZOPHRENIA PATHWAY 9
PROTEIN-PROTEIN INTERACTION ANALYSIS USING STRING 9
FIGURE S2. RESULTS OF INTERACTION ANALYSIS BY STRING 9
GENE ONTOLOGY ANALYSIS 10
TABLE S10. GO TERMS ENRICHMENT 11
TABLE S11. REFERENCES FOR CANDIDATE GENES FROM TDT STUDIES FOR FIG. 1 - 2. 12
PATHWAY ANALYSES 18
PRELIMINARY PATHWAY ANALYSES 18
TABLE S12. RESULTS OF PATHWAY ANALYSIS BY KEGG 20
FIGURE S3. KEGG'S MTOR PATHWAY 21
FIGURE S4. KEGG'S HYPOXIA-INDUCIBLE FACTOR (HIF) PATHWAY 22
FIGURE S5. KEGG'S VASCULAR SMOOTH MUSCLE CONTRACTION (VSMC) PATHWAY 23
TABLE S13. RESULTS OF PATHWAY ANALYSIS BY PANTHER 24
TABLE S14. DAVID'S GENETIC ASSOCIATIONS WITH DISEASES 25
GENE SET ANALYSIS 26
SUPPLEMENTARY STATISTICS INFORMATION 26

RESULTS 26

PUTATIVE SCHIZOPHRENIA PATHWAY 26
DISCOVERY SAMPLE 28

Table S15. Intersection between functional gene sets and candidate genes for schizophrenia obtained from TDT studies. 28
Replication sample #1 **30**
Table S16. Intersection between functional gene sets and candidate genes for schizophrenia obtained from case-control studies (CC). 30
Replication sample #2 **32**
Table S17. Intersection of the schizophrenia gene set obtained from the NHGRI catalog with functional gene sets. 32
Replication sample #3 **33**
Table S18. Intersection of the schizophrenia gene set developed by Ayalew et al.[4] with functional gene sets. 33
Replication sample #4 (all genes within range) **34**
Table S19. Intersection of all the 343 genes within range of 108 genome-wide significant loci reported in 2014 by the SWGPGC in the largest GWAS of schizophrenia to date. 34
Replication sample #4 (genes assigned by proximity) **35**
Table S20. Intersection between functional gene sets and 111 genes assigned by proximity to 108 genome-wide significant loci from the largest GWAS of schizophrenia to date. 35
Postmortem studies of schizophrenia **36**
Table S21. Intersection of functional gene sets with genes differentially expressed in postmortem brains of schizophrenic patients. 36
Overview of significant findings **37**
Table S22. Overview of significant findings. 37
Quasi experimental study **38**
Figure S6. Quasi-experimental impairment of components of the candidate pathway 38
Table S23. Quasi-experimental disturbance of candidate schizophrenia pathway 39
Table S24. Quasi-experimental neurodevelopmental disturbance 43

DISCUSSION **45**

Evidence for ischemia during schizophrenic psychosis **45**
Table S25. Supplementary references for Figure 4 45
Table S26. Signs of cerebral ischemia in adult schizophrenic patients 47
The foundation of the neurodevelopmental hypothesis can also be explained by adult vascular disorders **49**
Table S27. The foundation of the neurodevelopmental hypothesis and adult vascular disorder as alternative explanation. 49
Figure S7. Foundation of neurodevelopmental hypothesis and adult vascular disorder as alternative explanation. 52
Normal or superior intelligence in schizophrenia **53**
Birth complications in only 7% of schizophrenic patients **54**
Table S28. Percentage of schizophrenic patients with broadly defined birth complications 54
Schizophrenia treatments improve CBF and ischemia protection **55**
Table S29. Effects of treatments for schizophrenia on cerebral perfusion and ischemia. 55
Table S30. Evidence for repair mechanisms in schizophrenia. 56
The AVIH seems to offer a better explanation for the evidence compared to the NDH **57**
Table S31. Consilience of the NDH and the AVIH with the evidence 57

SUPPLEMENTARY REFERENCES **58**

SUPPLEMENTARY TABLES

Table S1 Discovery sample (33 TDT)

Table S2 Replication sample #1 (CC)

Table S3 Replication sample #2 (NHGRI)

Table S4 Replication sample #3 (Ayalew 2012)

Table S5 Replication sample #4 (SWGPGC 2014)

Table S6 VI genes

Table S7 ND genes

Table S8 SY genes

Table S9 R genes

Table S10 Results of GO

Table S11 References for Fig.1 & 2

Table S12 KEGG pathways

Table S13 PANTHER pathways

Table S14 DAVID genetic associations with diseases

Table S15 Results of discovery sample (TDT studies)

Table S16 Results of replication sample #1 (case-control studies)

Table S17 Results of using GWAS sample from NHGRI

Table S18 Results of using GWAS sample from Ayalew et al. (2012)

Table S19 Results of using 343 genes within range from large GWAS sample (SWGPGC 2014)

Table S20 Results of using 111 proximity-assigned genes from large GWAS sample (SWGPGC 2014)

Table S21 Results of using genes from postmortem studies

Table S22 Overview of significant findings

Table S23 Quasi-experimental disturbance of candidate pathway

Table S24 Quasi-experimental neurodevelopmental disturbance

Table S25 ref. for Fig. 4 (Penumbra model)

Table S26 Signs of cerebral ischemia during psychosis

Table S27 Evidence in support of ND hypothesis is associated with adult vascular disorders

Table S28 Percentage of schizophrenic patients with broadly defined birth complications

Table S29 Effects of treatments for schizophrenia on cerebral perfusion and ischemia

Table S30 Evidence for repair mechanisms in schizophrenia

Table S31 Consilience of the NDH and the AVIH with the evidence

SUPPLEMENTARY FIGURES

Figure S1 Venn diagrams

Figure S2 Results of STRING

Figure S3 KEGG mTOR pathway

Figure S4 KEGG HIF pathway

Figure S5 KEGG VSMC pathway

Figure S6 Quasi-experimental impairment of pathway components

Figure S7 Foundation of neurodevelopmental hypothesis supports adult vascular disorder interpretation

MATERIAL

Candidate genes for schizophrenia

Table S1. Discovery sample based on TDT-studies

A list of genes extracted from the SZgene database[1] hosted by the Schizophrenia Research Forum (http://www.szgene.org) was used as discovery sample. Reproducibility of association was considered more important than a low *p* value. The criterion for inclusion in this sample was a *p*-value ≤ 0.05 obtained by at least two independent research groups in TDT studies. As of July 7, 2011, the SZgene database contained 33 genes that fulfilled this criterion. The TDT-based 33 candidate genes of schizophrenia are listed below.

(1) AKT1, (2) ARVCF, (3) BDNF, (4) CHRFAM7A, (5) CHRNA7, (6) CLDN5, (7) COMT, (8) DAOA, (9) DISC1, (10) DRD2, (11) DRD3, (12) DTNBP1, (13) GABRB2, (14) GAD1, (15) TRMT2A, (16) HTR2A, (17) IPO5, (18) MTHFR, (19) NOS1AP, (20) NOTCH4, (21) NRG1, (22) NTNG1, (23) PLA2G4A, (24) PPP3CC, (25) PRODH, (26) RBFOX2, (27) RGS4, (28) DAT1, (29) 5HTT, (30) SYN2, (31) TNF, (32) UFD1L, (33) ZDHHC8.

This sample was employed as discovery sample for the construction of the putative schizophrenia pathway depicted in Figs. 1 and 2. The TDT gene sample was updated as requested by an anonymous reviewer during the peer review process. The update increased the number of TDT genes from 33 to 41. The additional genes were (34) ACSL6, (35) DRD1, (36) ERBB4, (37) GABRB2, (38) NRG3, (39) PIP5K2A, (40) SNAP25, (41) ZNF804A. The last TDT sample of 41 candidate genes obtained in December 2014 from the SZ database was employed for the intersection analysis of the combined five samples (discovery sample and four replication sample). The results are shown in Table 1.

Table S2. Replication sample #1 based on case-control studies

Because of the higher rate of false positive findings in case-control studies[2], the requirement for replication was increased. The choice of three independent groups for inclusion was guided by the need to validate the findings by the highest possible number of replications and to obtain at the same time a sufficiently large number of genes for statistical analysis. On July 7, 2011, 58 candidate genes for schizophrenia were found in the SZgene database[1], which showed positive results in case-control studies by three independent groups. The case-control based candidate genes (CC genes) were

(1) ACSL6, (2) AHI1, (3) AKT1, (4) APOE, (5) BDNF, (6) CCKAR, (7) CFB, (8) CHGB, (9) CHI3L1, (10) CHRNA7, (11) COMT, (12) DAO, (13) DAOA, (14) DISC1, (15) DRD2, (16) DRD3, (17) DRD4, (18) DRD5, (19) DTNBP1, (20) EGF, (21) ERBB4, (22) GABRB2, (23) GC, (24) GCLM, (25) GNB1L, (26) GRIK3, (27) GRIN1, (28) GRIN2B, (29) GRM3, (30) GSTM1, (31) HP, (32) HTR2A, (33) IL10, (34) IL1B, (35) IL1RN, (36) KCNN3, (37) MTHFR, (38) NOS1, (39) NOTCH4, (40) NRG1, (41) NRG3, (42) NTF3, (43) NTNG1, (44) PCM1, (45) PDE4B, (46) PIK3C3, (47) PIP4K2A, (48) PLA2G4A, (49) RELN, (50) RGS4, (51) SLC18A1, (52) SLC6A3, DAT1, (53) SLC6A4, 5HTT, (54) SNAP25, (55) TH, (56) TNF, (57) TPH1, (58) UFD1L

The latest update of the SZgene database obtained in December 2014 did not change the number of genes from case-control studies.

Genome-wide association studies (GWAS)

Table S3. Replication sample #2 obtained from the NHGRI catalog

The second replication sample consisted of 164 genes from the NHGRI catalog of GWAS[3] (available at: www.genome.gov/gwastudies/ accessed March 3, 2015). These genes were indicated in the catalog as mapped to loci associated with schizophrenia at a *p*-value threshold of $p < 10E\text{-}08$ and were obtained from 45 GWAS of schizophrenia (PubMed IDs and first authors are given in the following Table S3 (see http://www.ncbi.nlm.nih.gov/pubmed). The dates of publication ranged from 2007 until 2014, but did not include the large-scale GWAS by the SWGPGC (2014).

PMID	First author	PMID	First author
24280982	Ruderfer 2014	21752600	Chen 2011
24043878	Wong 2014	21679298	Ma 2011
23358160	Borglum 2014	21674006	Yamada 2011
24253340	Lencz 2013	21107309	McClay 2011
24166486	Sleiman 2013	21057379	Curtis 2011
24086445	Wang 2013	20939080	Greenbaum 2010
24039173	McGrath 2013	20889312	Wang 2010
23974872	Ripke 2013	20713499	Huang 2010
23453885	Smoller 2013	20558996	Ott 2010
23894747	Aberg 2013	20185149	Athanasiu 2010
23142968	Betcheva 2013	19571811	Purcell 2009
23382809	Xu 2013	19571808	Stefansson 2009
23212062	Fanous 2012	19571809	Shi 2009
22885689	Levinson 2012	19197363	Need 2009
22883433	ISGC 2012	19023125	Potkin 2009
22688191	Bergen 2012	18677311	O'Donovan 2008
22648509	Wang 2012	18369103	Walsh 2008
22479419	Liou 2012	18347602	Sullivan 2008
21747397	Rietschel 2012	18332876	Kirov 2009
21682944	Alkelai 2012	18282107	Shifman 2008
22037555	Shi 2011	17522711	Lencz 2007
22037552	Yue 2011		
21926974	Ripke 2011		
21795503	Alkelai 2011		

Table S4. Replication sample #3 obtained from Ayalew et al. (2012)

An intersection analyses was also performed of the carefully selected set of 42 genes from GWAS described by Ayalew et al. (2012)[4]. This replication sample is characterised by high reproducibility and predictive ability in four independent cohorts of different ethnicities[4].

Table S5. Replication sample # 4 obtained from SWGPGC 2014

A fourth replication sample consisted of 111 genes assigned by proximity to the 108 genome-wide significant regions of the SWGPGC's recent large GWAS[5]. Genes were assigned to index-SNPs using a 50 kb window. If multiple genes were present within the 50 kb window, the hierachy described by Torkamani et al.[6] was employed: coding > intronic > 5'UTR > 3'UTR > 5' upstream > 3' upstream > nearest gene. Additionally, the entire list of 343 genes within range of genome-wide significant loci (as given by the SWGPGC in column 5 of their supplementary Table 3) was used to exclude the possibility of a bias by the gene assignment.

Functional gene sets

Table S6. Vascular and acute ischemia genes of the adult brain

A list of 2 866 ischemia or reperfusion-induced genes were obtained by literature mining from 61 sources including 75 genes from the Entrez Genes database of Homo sapiens (HS), which were extracted by use of the keywords hypoxia, ischemia, ischemic, erythropoietin or vascular. The remainder of the genes came mostly from gene-expression studies of the adult brain reported in the literature[7-65]. After removal of duplicate genes, a set of 1673 ischemia genes remained for statistical analysis.

The gene set for vascular genes consists of 3 500 genes involved in perivascular nerves[66-74] (N = 253), capillary endothelial cells[75] (N = 20), brain endothelial cells[76] (N = 301), capillary shear-stress[77-93] (N = 2 818), blood-brain barrier (BBB)[94] (N = 29) and vascular smooth muscle cells (VSMC)[95] (N = 79). After the removal of duplicates, 3 249 vascular genes remained.

Table S7. Neurodevelopmental genes

A list of 4 020 differentially expressed genes during neurodevelopment was extracted from 18 published studies[96-113]. The Entrez Genes database of HS[114] had no entries for the key words neurodevelopment or brain development. After removal of duplicates, a list of 3 211 neurodevelopmental genes remained for further analysis.

Table S8. Synaptic genes

A list of 2 988 synaptic genes was obtained from the Entrez Genes database of HS, from Bayés et al. ground-breaking study of human Postsynaptic Densities (hPSD)[115] and a list of 1 480 genes was compiled from two additional references[116,117]. The Entrez Genes database provided 50 genes, which were extracted by using the keywords synapse or synaptic. The hPSD study yielded 1 458 genes. Since 1 011 genes were found in several datasets, a gene set of 1 977 synaptic genes remained after removing duplicates.

Table S9. Post-ischemic repair genes

A list of 159 genes involved in post-ischemia repair was compiled from the literature[118-126]. They are designated as "R" in Tables and Figures. Since ND genes are involved in adult neurogenesis and post-ischemic repair[120,127], R and ND genes were combined and termed "Repair".

Figure S1. Venn diagrams

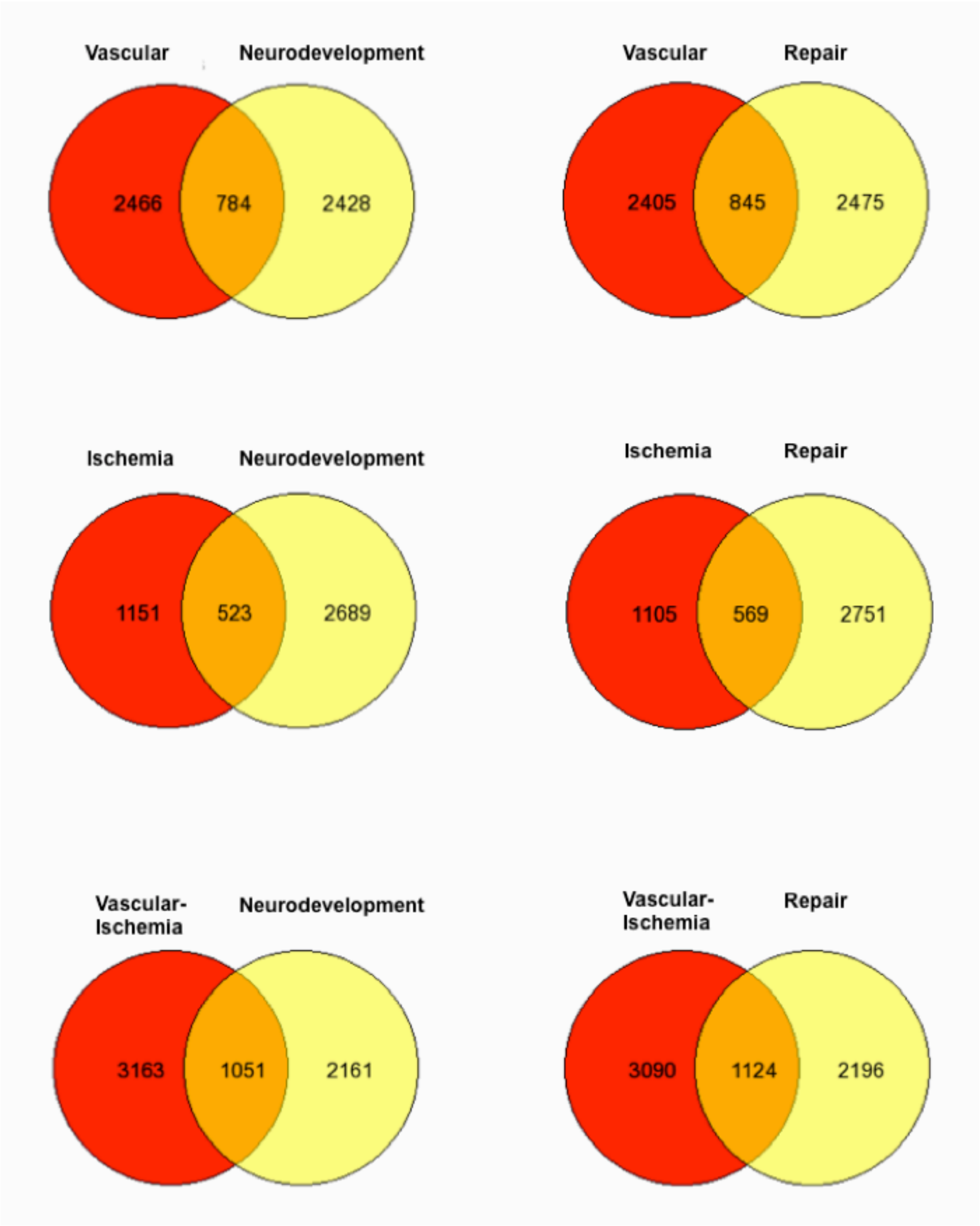

Figure S1. Venn diagram of vascular, ischemia, and vascular-ischemia genes overlapping with neurodevelopmental or repair genes. The diagram was produced by using GeneVenn (available at http://genevenn.sourceforge.net).

METHODS

Construction of schizophrenia pathway

Protein-protein interaction analysis using STRING

For protein-proteins interaction analysis, STRING (**S**earch **T**ool for the **R**etrieval of **In**teracting **G**enes/Proteins) version 8 at (http://string-db.org) was employed[128]. Supplementary Fig. S1 shows the protein-protein interactions of candidate genes for schizophrenia used in the present study.

Figure S2. Results of interaction analysis by STRING

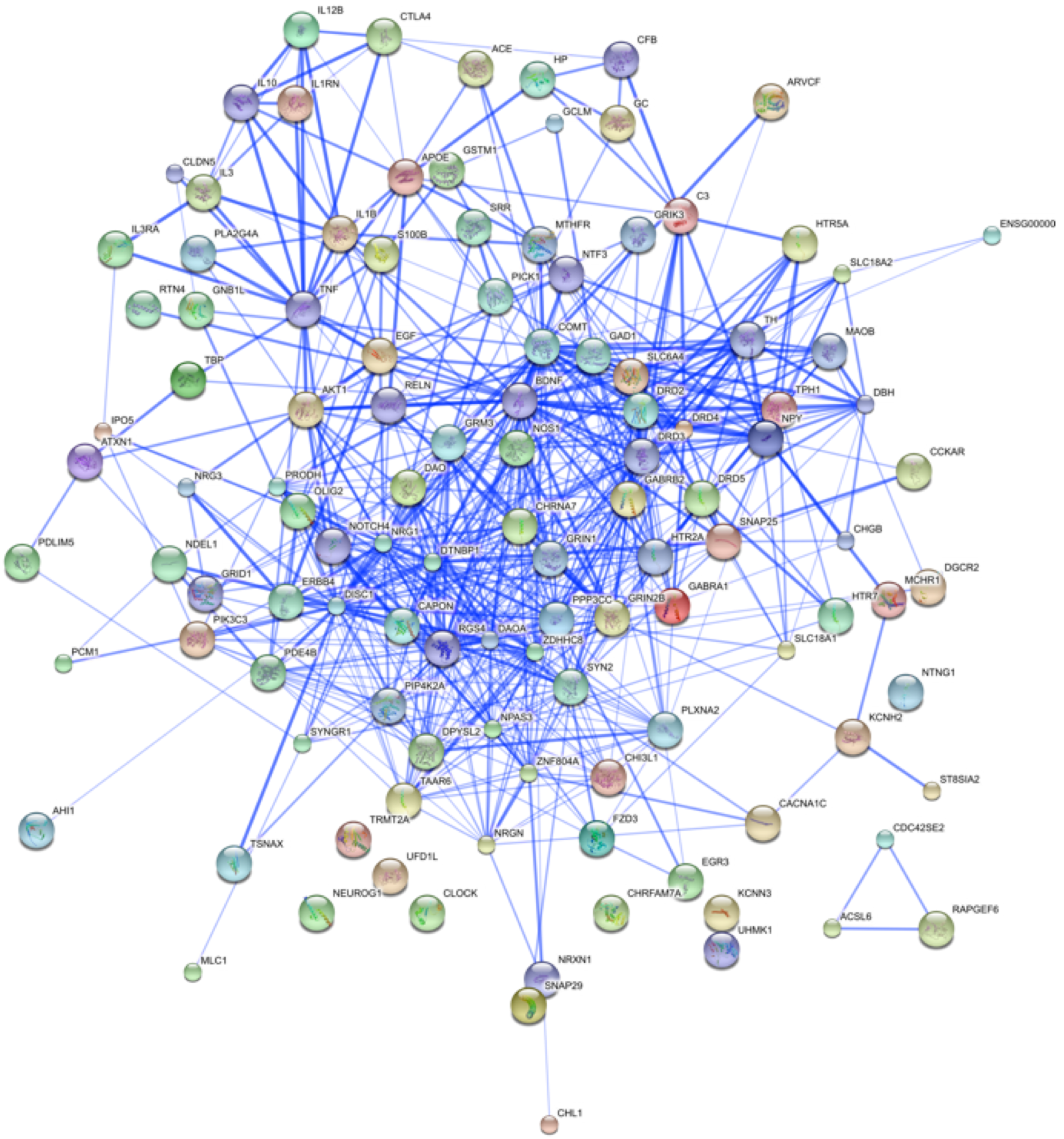

Figure S2. STRING's evidence view revealing protein-protein interactions of candidate genes for schizophrenia employed in the present study. Data from the STRING database were used for constructing the energy-supply pathway shown in Figs. 1 and 2.

Gene ontology analysis

The gene ontology (GO) database of the Gene Ontology Consortium[129] at (http://www.geneontology.org) was employed to search for overrepresentation of biological processes or cellular localisations among the genes of the discovery sample.

As requested by a reviewer, the results of such an analysis are shown in Supplementary Table S8. The 125 schizophrenia-associated genes of the combined four samples were employed for the GO term analysis. The GO analysis produced a long list of significant biological processes, which are difficult to interpret with regard to an unitary etiological factor. Only the first page is shown.

Table S10. GO terms enrichment

AmiGO 2

Home Search Tools & Resources Help Feedback About AmiGO 1.8

Quick search Search

Term Enrichment Results

Information about Term Enrichment Results

Current documentation and discussion of term enrichment with the GO can be found here.

This is a work in progress, so your feedback is appreciated.

Results Produced by PANTHER

Run with different parameters (reset); currently: **format:** xml **input count:** 125 **species:** HUMAN **ontology:** biological_process **correction:** bonferroni

Reference mapped/unmapped: 21804/0 **Input list mapped/unmapped:** 118/7
Unmapped inputs: *267012 6854 64002 100616252 64478 256021 374470*

Download as: raw XML raw tab-delimited

Term	Background frequency	Sample frequency	Expected	+/-	P-value
synaptic transmission (GO:0007268)	616	39	3.334e+00	+	4.809e-28
cell-cell signaling (GO:0007267)	943	42	5.103e+00	+	1.496e-24
regulation of synaptic transmission (GO:0050804)	239	24	1.293e+00	+	1.014e-20
learning or memory (GO:0007611)	192	21	1.039e+00	+	1.269e-18
cognition (GO:0050890)	221	21	1.196e+00	+	2.150e-17
behavior (GO:0007610)	473	26	2.560e+00	+	3.250e-16
response to organonitrogen compound (GO:0010243)	707	30	3.826e+00	+	5.347e-16
neurogenesis (GO:0022008)	1312	38	7.100e+00	+	1.814e-15
memory (GO:0007613)	90	15	4.871e-01	+	1.820e-15
generation of neurons (GO:0048699)	1237	37	6.694e+00	+	2.017e-15
system development (GO:0048731)	3692	61	1.998e+01	+	2.793e-15
response to nitrogen compound (GO:1901698)	769	30	4.162e+00	+	5.188e-15
neuron-neuron synaptic transmission (GO:0007270)	57	13	3.085e-01	+	6.259e-15
regulation of multicellular organismal process (GO:0051239)	2121	46	1.148e+01	+	1.284e-14
single-multicellular organism process (GO:0044707)	5739	74	3.106e+01	+	5.401e-14
[illegible]	[illegible]	[illegible]	[illegible]	[illegible]	[illegible]

Table S11. References for candidate genes from TDT studies for Fig. 1 - 2.

Gene (Entrez Gene ID) functions and localizations
**1. AKT1** (PKB) (207) is part of the PI3K-AKT-mTOR pathway (KEGG's pathway hsa04012+207) (see Fig. 2 in article), which regulates essential cellular functions such as glucose metabolism, growth, vascular homeostasis, angiogenesis, expression and activity of pro- and anti-angiogenic factors, activity of nitric oxide synthase (eNOS), NO production[130], contraction and relaxation of blood vessels induced by various agents[131]. Furthermore, it enhances vascular function[130,132,133], augments Hypoxia-Inducible Factor-1A (HIF1A) expression by increasing protein translation through a mammalian target of rapamycin (mTOR)[134], reduces ischemic damage[134,135] and is a vital cytoprotectant for vascular and neuronal cells[135].
**2. ARVCF** (421) The armadillo repeat gene deleted in velocardiofacial syndrome (ARVCF) associates with E-cadherin[136], which is part of the tight junctions between the endothelial cells of blood vessels. E-cadherin is important for the function of the blood–brain barrier (BBB). Short exposures to ischemia cause a decreased expression of E-cadherin and harm the BBB[137].
**3. BDNF** (627), brain-derived neurotrophic factor, attenuates microvascular permeability disturbances and axonal injury[138], prevents ischemia-induced neuronal cell death in the hippocampus[139] and plays a role in ischemic preconditioning[140].
**4. CHRNA7** (1139), cholinergic receptor, nicotinic, alpha 7, is expressed in vascular smooth muscle cells[141] and induces cerebral vasodilatation[142]. Acetylcholine is known to induce dilation of intracortical microvessels and an increase in cortical perfusion[143].
**5. CHRFAM7A** (89832) CHRFAM7A, cholinergic receptor, nicotinic, alpha 7, exons 5-10, is a partially duplicated variant of CHRNA7. It is unknown but possible that CHRFAM7A is translated and that the gene product is able to interact with alpha 7 polypeptide since most of the contact regions are encoded in exons 5–10. Furthermore, CHRFAM7A has been found to be associated with four types of dementia, Alzheimer's disease, dementia with Lewy bodies, Pick's disease and vascular dementia[144].
**6. CLDN5** (7122), claudin 5 is a major cell adhesion molecule of tight junctions in brain endothelial cells which function as blood-brain barrier (BBB). Hypoxia disrupts the BBB function through changes in the expression of claudin 5[145].
**7. COMT** (1312), catechol-O-methyltransferase, plays an important role in the metabolic degradation of the catecholamine neurotransmitters dopamine, adrenaline and noradrenaline. It is also involved in the vasoconstriction of cortical microvessels by dopamine[69].
**8. DAOA** (267012), D-amino acid oxidase, an enzyme that degrades D-serine, markedly inhibites neuronal damage by cortical ischemia and N-methyl-D-aspartate (NMDA)[29]. One isoform appears to play a role in mitochondrial function[146]. Furthermore, interaction of DAOA with COMT has been observed[147].

9. EGLN1, PHD2, disrupted in schizophrenia 1 (DISC1) (54583). The schizophrenia-associated Leu607Phe polymorphism hinders the axonal transport of mitochondria required for energy production in presynaptic terminals[148]. Furthermore, EGLN1 is part of the HIF-VHL-prolyl hydroxylase pathway[149], which functions as a cellular oxygen sensor and, under normoxic conditions, targets the hypoxia-inducible factor (HIF-1) alpha protein through hydroxylation for ubiquitination and proteasomal degradation via the von Hippel-Lindau (VHL) complex[150,151]. HIF-1 is a transcriptional complex that plays a central role in mammalian oxygen homeostasis and regulates, under hypoxic conditions, the transcription of numerous genes related to angiogenesis, cell survival, and glucose metabolism[152].

10. DRD2 (1813), dopamine receptor D2 is involved in peripheral vasoconstriction[153]. With regard to the brain, central dopaminergic neurones make close contacts with the basal lamina of arterioles and with astrocytic end-feet (reviewed in[154]). Microinjection of dopamine causes a pronounced constriction of cerebral microvessels[69]. Moreover, D2 receptor agonists produced negative changes in regional cerebral blood volume (rCBV). On the other hand, D1/D5 receptor agonists and DAT blockers induce positive hemodynamic changes[155].

11. DRD3 (1814). Similar to DRD2, D3 dopamine receptors are involved in peripheral vasoconstriction[153]. In the brain, D3 receptors are expressed by all astrocytes[156], by 75% of capillary endothelial cells, 25% of capillaries, and 40% of microvessels. D3 receptor agonists cause negative changes in rCBV[155].

12. DTNBP1 (84062), dystrobrevin binding protein 1. Dystrobrevin and probably its binding protein is localized in the astrocytic endfeet and endothelial cells of cerebral microvessels[157,158] and is part of the signal-transduction pathway for the α1D-adrenergic receptor (α1D-AR). The latter are ubiquitously expressed on vascular smooth muscle, cause vasoconstriction when activated by noradrenaline and adrenaline[159] and are responsible for increased blood pressure during exercise, injury, and stress (reviewed in[160]).

13. GABRB2 (2561), gamma-aminobutyric acid A (GABA-A) receptor beta 2. Cortical GABA interneurons provide a rich innervation to local microvessels and appear to act as local integrators for the tight coupling of neuronal activity and local perfusion, which is essential for normal brain function[161]. GABA-A receptors are present in cerebral microvessels[162,163] and respond by vasodilatation to GABA released from nerve terminals[163,164]. Muscimol, a GABA-A receptor agonist, elicites vasodilation in hippocampal microvessels[163]. Vasodilatation by cholinergic neurons is in part mediated by the local release of GABA from cholinoceptive cortical interneurons and through GABA-A receptors[165].

The transcription of GABRB2 itself is highly sensitive to hypoxia[166] and GABA-A receptors are involved in BBB disruption during cerebral ischemia[167]. With regard to cerebral ischemia, GABA exerts neuroprotective effects (reviewed in[168,169]) via GABA(A) and GABA(B) receptors[170]. And GABAergic interneurons survive ischemic injury for up to 30 days in all investigated brain regions[171]. Finally, the induction of ischemic tolerance by preconditioning depends on functional modifications of GABA synapses[172].

14. GAD1 (2571), glutamate decarboxylase 1 (brain, 67kDa) is responsible for catalyzing the production of gamma-aminobutyric acid (GABA) from L-glutamic acid. For GABA's

localisation and role in cortical microvessels, see GABRB2 above.
**15. HTR2A** (3356), 5-hydroxytryptamine (serotonin) receptor 2A mediates vasoconstrictive responses to 5-HT in many vascular smooth muscles and also potentiates the activity of growth factors (reviewed in[173]). Intracerebrally released serotonin cause a decrease of cerebral blood flow (CBF) in several brain regions such as the neocortex suggesting a major vasoconstrictor role[174]. In the CNS, 5-HT2A receptors are abundant in the cerebral cortex and the limbic system and are expressed in neurons as well as in astrocytes[175]. Astrocytes are involved in the regulation of cerebral blood flow (CBF) (reviewed in[176]). In addition, the HTR2A gene appears to be associated with ischemic stroke[177]. Antagonists of the 5-HT2A receptor such as ketanserin and ritanserin increase CBF in cortical areas and reduce ischemic damage (reviewed in[174]). Finally, atypical antipsychotic drugs (such as clozapine, aripiprazole, asenapine, iloperidone, lurasidone, olanzapine, quetiapine, risperidone, and ziprasidone) produce extensive blockade of serotonin 5-HT2A receptors and stimulation of 5-HT1A receptors at clinically effective doses[178]. Both , i.e. 5-HT1A receptor agonists and 5-HT2 receptor antagonists, have a neuroprotective effect against ischemia-induced deficits[179].
**16. IPO5** (3843) also known as imp5, RANBP5, IMB3, Pse1, and KPNB3 encodes the importin 5 protein, which is a member of the importin beta family, a cytoplasmic protein that binds to nuclear pore complexes (NPCs)[180], imports ribosomal proteins in the nucleolus where they are assembled into the eukaryotic ribosomal subunits required for protein synthesis[181] and mediates the nuclear import of H2A, H2B, H3 and H4 histones[182]. These four core histones - H2A such as Hist1H2AG, H2B such as Hist1H2BJ, H3 and H4 - are localized on chromosome 6p22, a region showing a strong association with schizophrenia[183]. The import of the four core histones is essential for the S-phase of the cell cycle during which DNA is replicated and newly synthesized histones are deposited onto the DNA in order to form the chromatin structure[182] (reviewed in[184]).<br><br>The S-phase of the cell cycle is important for the proliferation of vascular endothelial cells during angiogenesis (reviews[185-187]). Angiogenesis is a predictive marker of neurological outcome following hypoxia-ischemia[188,189]. Furthermore, histones H4[190], H3, and H2A are known to play a role in ischemia protection[190-194].
**17. MTHFR** (4524) methylene tetrahydrofolate reductase catalyzes the reduction of 5,10-methylene tetrahydrofolate to 5-methyl tetrahydrofolate, the predominant ciruclatory form of folate and carbon donor for the re-methylation of homocysteine to methionine. Two polymorphisms are known to cause mild enzyme deficiency. A common polymorphism in the MTHFR gene (C677T, Ala --> Val) is associated with a decreased activity of the enzyme due to thermolability. In case of homozygosity for the Val allele, a relative deficiency of the enzyme leads to a mild-to-moderate hyperhomocysteinaemia[195]. Hyperhomocysteinemia is a risk factor for cerebrovascular disease[196].
**18. NOS1AP** (9722) nitric oxide synthase 1 (neuronal) adaptor protein, alias CAPON, encodes a cytosolic adapter protein that activates neuronal nitric oxide synthase (nNOS/NOS1) and is involved in the synthesis of nitric oxide (NO)[197]. In humans, nNOS produces NO in nitric oxide interneurons and vascular smooth muscle cells regulating microvascular tone in humans[198-200]. In brain ischemia, nNOS stimulates the increase of NO from baseline nanomolar to micromolar levels NO (reviewed in[201]). However, the activation of nNOS alone has neurotoxic effects[202], whereas simultaneous activation of

eNOS appears to be neuroprotective (reviewed in[201,203]).
**19. NOTCH4** (4855) alias INT3, encodes for the Notch-4 protein, an endothelial cell specific homologue of Notch. The expression of NOTCH4 is restricted to endothelial cells in the embryonic and adult brain[204]. The Notch-4 protein plays a crucial role in vasculogenesis, vascular repair of injury and angiogenesis[205,206]. The latter is a key response to cerebral ischemia[185-187] and predicts the neurological outcome[188,189]. During angiogenesis, Notch-4 induces microvessel differentiation of brain endothelial cells[207] and the formation of new blood vessels from existing vasculature. To allow for endothelial sprouting, the extracellular matrix around existing vasculature is degraded by matrix metalloproteases (MMPs)[208]. The MMPs is induced by VEGF signaling via VEGFR-2 and the PI3K/Akt pathway[209].
**20. NRG1** (3084) neuregulin 1 alias glial growth factor 2 - also known as GGF; HGL; HRG; NDF; ARIA; GGF2; HRG1; HRGA; SMDF; MST131 – and two of its receptors (erbB2, erbB3) are expressed in brain microvascular endothelial cells[210], astrocytes and oligodendrocytes[211,212]. Neuregulin 1 activates the PI3K/AKT intracellular signaling pathway by binding to erbB receptors[211,213]. Cellular survival after ischemia depends in large extent on the activation of the PI3K/Akt pathway (reviewed in[214,215]). Like other growth factors, NRG1 activates the PI3K/Akt pathway and subsequently the mTOR-dependent protein synthesis[210,213,216,217] required for ischemia protection and repair. NRG1 has been shown to be a powerful neuroprotective factor in ischemia[119,218-220] and to play a role in repair[119]. Following vessel hypoxia and injury, the expression of of NRG1 and erbB is upregulated whereas in uninjured vessels it is low[218,221,222] .
**21. NTNG1** (22854) Netrin G1 belongs to a conserved family of proteins that act as axon guidance cues during vertebrate nervous system development[114,223].<br><br>Another member of this family Netrin-1, has the ability to attract blood vessels as well as axons, and is capable of functioning as a vascular growth factor[224]. In addition, netrin-1 stimulates NO production in mature endothelial cells[225] and has been shown to protect the cerebral cortex from the effect of ischemia[226].<br><br>Little is known about a possible vascular function of netrin G1. However, the fact that the trajectories of nerves and blood vessels are often shared, led to the hypothesis that tissues may use identical or similar factors to guide innervation and vascularization[227]. Human NTNG1 is localized at the chromosomal position 1p13.3. This region is syntenic with mouse chromosome 3, where a modifier locus for renal vascular disease lesions has been identified[228].
**22. PLA2G4A** (5321) (alias cPLA2-alpha). The gene product, cytosolic, calcium – dependent phospholipase A2 (cPLA2), is expressed in astrocytes[229], mediates agonist-induced release of AA[230,231], responds to stress, inflammation, G protein-coupled receptors, adrenoreceptor-mediated vasoconstriction[232] and ischemia[233]. AA is further metabolized into prostaglandin molecules by cyclooxygenase-2 (COX-2) causing relaxation of vascular smooth muscles and subsequent vasodilatation. This mechanism is also activated by the skin flush induced by niacin (nicotinic acid)[231,234-239]. AA are metabolized by cytochrome P450 (CYP) epoxygenase to form epoxyeicosatrienoic acids (EETs) which are known key astrocyte- and endothelium-derived regulators of cerebrovascular function[240]. EETs have been shown to protect astrocytes and neurons against ischemia and to be key regulators of

cortical angiogenesis, which is important for recovery from ischemia[241] (reviewed in[240]).

23. PPP3CC (5533) PPP3CC (aliases CNA3; CALNA3; PP2Bgamma) codes for calcineurin A gamma subunit[114]. Calcineurin is a serine/threonine phosphatase that is activated by calcium and calmodulin[242]. It promotes the expression of Hypoxia-inducible Factor 1 alpha (HIF-1α) via the receptor for activated C kinase 1 (RACK1)[242]. Highly localized in the brain, especially in those parts which are vulnerable to hypoxia/ischemia, it has protective as well as toxic effects and the balance may be important for the outcome of ischemia[243].

24. PRODH (5625) encodes proline oxidase (POX), a mitochondrial inner-membrane enzyme that metabolizes l-proline. Most of the alleles associated with schizophrenia result in severely reducted POX activity and hyperprolinemia[244]. The latter impairs the activity of cytochrome c oxidase, an enzyme of the respiratory electron transport chain of mitochondria[245]. In the cerebral cortex, proline causes mitochondrial dysfunction, oxidative stress and impaired energy metabolism[246].

25. RBFOX2 (23543) alias Rbm9, RNA binding protein, fox-1 homolog. The mammalian Fox genes are complex transcription units that specifically recognize the RNA element UGCAUG and generate transcripts from multiple promoters[247]. Fox-1/2 are preferentially expressed in brain, heart and muscle tissues. They target genes involved in muscle contraction and vascular regulation, such as potassium ion transport, myosin, dystrophin, calmodulin binding[92].

Potassium (K+) channels play an important role in neurovascular coupling (reviewed in[248]), cerebral ischemia (reviewed in[249]) and endothelial dysfunction (reviewed in[250]). Myosin is expressed in vascular smooth muscle and pericytes[251] suggesting a role in vasoconstriction. Dystrophin is involved in flow (shear stress)-induced endothelium-dependent dilation and its absence in mice reduces NO-dependent vascular function[252]. Calmodulin and calcium activate calcineurin, which promotes the expression of hypoxia-inducible Factor 1 alpha (HIF-1α)[242].

26. RGS4 (5999), regulator of G-protein signaling 4, is selectively enriched in the heart and brain (reviewed in[253]). RGS proteins modulate hormone and neurotransmitter signaling[254]. With regard to the former, insulin release from pancreatic beta-cells is negatively regulated by RGS4[255]. Insulin activates the PI3K/Akt pathway, which is important for ischemia protection and repair from ischemia injury by angiogenesis. The latter is inhibited by RGS4[256]. Concerning neurotransmitter signaling, RGS proteins modulate and inhibit signal transduction by G-protein-coupled receptors (GPCRs) (reviewed in[253,257,258]). Mice deficient for RGS4 show increased concentration of serum catecholamines[259]. In addition, RGS4 is linked to regulation of cholinergic and serotonergic signaling in the brain and is expressed in most cortical layers (reviewed in[253]). GPCRs are widely associated with the regulation of vascular smooth muscle cell contractility[260] and RGS proteins are known to play a role in the regulation of vascular tone[261,262].

27. SLC6A3, DAT1 (6531), solute carrier family 6 (neurotransmitter transporter, dopamine) member 3, is situated in the plasma membrane of the dopaminergic neurons where it mediates the re-uptake of dopamine from the synaptic cleft into the presynaptic neuron[263,264]. Dopaminergic signaling in the brain is primarily modulated by dopamine

transporters (DATs) (reviewed in[265]). In rats, DAT blockers induce positive hemodynamic changes via D1/D5 dopamine receptors and smaller negative changes through D2/D3 receptors on microvessels and astrocytes[155]. In humans, the DAT blocker cocaine caused dose-dependent cerebral vasoconstriction as revealed by magnetic resonance angiography[266]. Cocaine abuse and dependence is associated with increased incidence of stroke and myocardial ischemia[267,268]. The latter has been shown to be a consequence of vascular spasms[268].
**28. SLC6A4, 5HTT** (6532), solute carrier family 6 (neurotransmitter transporter, serotonin) member 4 - also known as HTT; 5HTT; OCD1; SERT; 5-HTT; SERT1; hSERT; 5-HTTLPR - encodes a membrane protein that transports the neurotransmitter serotonin from synaptic spaces into presynaptic neurons. It terminates the action of serotonin and recycles it[114]. Serotonergic perivascular nerves are involved in the regulation of cerebrovascular tone[68]. Intracerebrally released serotonin (5-HT) has a major vasoconstrictor effect resulting in a decrease of cerebral blood flow (CBF) in several brain regions including the neocortex (reviewed in[174]). A serotoninergic pathway originating in the raphe nucleus projects to cortical microvessels endowed with several 5-HT receptors including 5-HT1B receptors that mediate their contraction (reviewed in[68,174]). Consistent with serotonin's vasoconstrictor effect, 5-HT2 receptor antagonists such as ketanserin and ritanserin, have been shown to increase CBF in cortical areas and to exert a protective effect in ischemia[174].
**29. SYN2** (6854), synapsin II - is a member of the synapsin family and encodes a neuron-specific phosphoprotein that selectively binds to small synaptic vesicles in the nerve terminal[114]. Synapsin proteins have important functions in maintaining the integrity and stability of synaptic vesicles[269] and are regulators of neurotransmitter release from presynaptic nerve terminals (reviewed in[270,271]).<br><br>SNYN2 is a negative regulator of catecholamine release. SYN2 knock-out mice showed an increase of catecholamine release[272]. Furthermore, double knock-out mice, with deletions of SYN1 and SYN2, display higher concentrations of acetylcholine in the cortex[269]. SYN2 knock-out mice also had an increase of glutamatergic and GABAergic synaptic transmission in the spinal cord after nerve injury[273]. Catecholamines and acetylcholine play a role in neurovascular regulation (reviewed in[68]). Glutamate release following ischemia is thought to cause neuronal injury (reviewed in[274]).
**30. TRMT2A** (27037), HpaII tiny fragments locus 9c protein, HTF9C, TRM2 tRNA methyltransferase 2 homolog A is a protein expressed in proliferating cells. It is overexpressed in breast cancer[275] suggesting a role for TRMT2A in angiogenesis and in protection and recovery from ischemia. Angiogenesis is predictive of neurological outcome following hypoxia-ischemia[188,189].<br><br>The transcription of TRMT2A is repressed in quiescent tissues and growth-arrested cells, activated at the G1/S transition of the cell cycle, and peaks in S phase[276,277]. The G1/S transition is the first brake-point through which the cell must pass before it can enter cell division. During S-phase of the cell cycle, DNA is replicated and de novo chromatin assembly takes place[278].<br><br>A key response to tissue hypoxia is angiogenesis, which requires the proliferation of vascular endothelial cells (reviewed in[185-187]). Prevention of endothelial cells to enter G1 phase of the cell cycle results in reduced angiogenesis[279,280] and hence in protection and

recovery from ischemia.
**31. TNF** (7124), tumor necrosis factor, is a multifunctional proinflammatory cytokine[114] which is induced within 1 hour in brain ischemia, It has oligodendrocyte cytotoxic as well as neuroprotective effects (reviewed in[281,282]). The activation of the Akt pathway has protective effects on TNF-mediated oligodendrocyte cytotoxicity[283]. Concerning neuroprotection, TNF activates also the mammalian target of rapamycin (mTOR) which has an influence on mitochondrial energy metabolism, protein synthesis and adaptation to ischemia[284]. Moreover, TNF activates cPLA2 (reviewed in[285]), which regulates cerebrovascular function via arachidonic acid (AA) and epoxyeicosatrienoic acids (EETs) (reviewed in[240,286]). Finally, TNF improves ischemia repair by upregulating the erythropoietin receptor (EPOR) thereby sensitizing cerebral endothelial cells for erythropoietin-induced angiogenesis[287].
**32. UFD1L** (7353), ubiquitin fusion degradation 1 like. The protein encoded by this gene forms a complex with two other proteins, nuclear protein localisation-4 and valosin-containing protein. This complex is necessary for the degradation of ubiquitinated proteins[114]. Ubiquitination of proteins is the first step in the degradation of proteins by the proteasome system. The ubiquitin-proteasome system degrades hypoxia-inducible factor 1alpha (HIF-1alpha) protein under normoxic conditions, while it is stabilized and accumulated rapidly following exposure to low oxygen tensions[288,289]. HIF-1 is a master regulator of response to hypoxia by activating the transcription of many genes, including those involved in blood flow, cell survival, glucose transport, energy metabolism, i.e. genes whose protein products increase oxygen delivery or facilitate adaptation to hypoxia[114]. Ubiquitin fusion degradation protein 1 (UFD1) is a blood marker for the early diagnosis of ischemic stroke[290].
**33. ZDHHC8** (29801), zinc finger, DHHC-type containing 8 (DHCC8), is localised in mitochondria and presynaptic processes, mostly glutmatergic and to a lesser extent GABAergic processes. It interacts interacts with mitochondrial Complex III. ZDHHC8 dosage change is able to disrupt mitochondrial function and to influence cell survival and death[291].

Pathway analyses

Preliminary pathway analyses

KEGG pathway analysis

The Kyoto Encyclopedia of Genes and Genomes (KEGG)[292] at (http://www.kegg.jp) was used for pathway analysis and as guide for the construction of the candidate schizophrenia pathway depicted in Figs. 1 and 2.

As requested by one of the reviewers, results of these analyses are shown as Supplementary Information. The KEGG pathway analyses produced a five pages long list of pathways, which are difficult to interpret with regard to the etiology of schizophrenia. Table S12 lists the top results on the first page of output from KEGG. Figs. S3S5 are

shown to exemplify the help provided by KEGG pathways for constructing the candidate pathway for schizophrenia depicted in Figs. 1 and 2.

The first page of the results from the KEGG pathway analysis is shown in Table S12. Five genes from our list of candidate genes for schizophrenia were not found in the KEGG database. Yellow highlights pathways related to vascular regulation or the energy-delivering pathway depicted in Figs. 1 and 2.

Table S12. Results of pathway analysis by KEGG

Pathway Search Result

Following object(s) was/were not found ncbi-geneid:54806 ncbi-geneid:421 ncbi-geneid:89832 ncbi-geneid:267012 ncbi-geneid:27185 ncbi-geneid:84062 ncbi-geneid:3843 ncbi-geneid:5999 ncbi-geneid:6854 ncbi-geneid:27037 ncbi-geneid:29801 ncbi-geneid:91752

Sort by the pathway list

Show all objects

- hsa04080 Neuroactive ligand-receptor interaction - Homo sapiens (human) (7)
- hsa04728 Dopaminergic synapse - Homo sapiens (human) (7)
- hsa01100 Metabolic pathways - Homo sapiens (human) (6)
- hsa04010 MAPK signaling pathway - Homo sapiens (human) (5)
- hsa04020 Calcium signaling pathway - Homo sapiens (human) (5)
- hsa04012 ErbB signaling pathway - Homo sapiens (human) (4)
- hsa04726 Serotonergic synapse - Homo sapiens (human) (4)
- hsa05034 Alcoholism - Homo sapiens (human) (4)
- hsa04024 cAMP signaling pathway - Homo sapiens (human) (4)
- hsa05030 Cocaine addiction - Homo sapiens (human) (4)
- hsa04370 VEGF signaling pathway - Homo sapiens (human) (3)
- hsa04664 Fc epsilon RI signaling pathw [illegible] sapiens (human) (3)
- hsa04540 Gap junction - Homo sapiens (human) (3)
- hsa05031 Amphetamine addiction - Homo sapiens (human) (3)
- hsa04380 Osteoclast differentiation - Homo sapiens (human) (3)
- hsa05166 HTLV-I infection - Homo sapiens (human) (3)
- hsa05205 Proteoglycans in cancer - Homo sapiens (human) (3)
- hsa05012 Parkinson's disease - Homo sapiens (human) (3)
- hsa05033 Nicotine addiction - Homo sapiens (human) (3)
- hsa04210 Apoptosis - Homo sapiens (human) (3)
- hsa04727 GABAergic synapse - Homo sapiens (human) (3)
- hsa05032 Morphine addiction - Homo sapiens (human) (3)
- hsa04660 T cell receptor signaling pathway - Homo sapiens (human) (3)
- hsa05152 Tuberculosis - Homo sapiens (human) (3)
- hsa04920 Adipocytokine signaling pathway - Homo sapiens (human) (3)
- hsa05160 Hepatitis C - Homo sapiens (human) (3)
- hsa04150 mTOR signaling pathway - Homo sapiens (human) (2)
- hsa04921 Oxytocin signaling pathway - Homo sapiens (human) (2)

Figure S3. KEGG's mTOR pathway

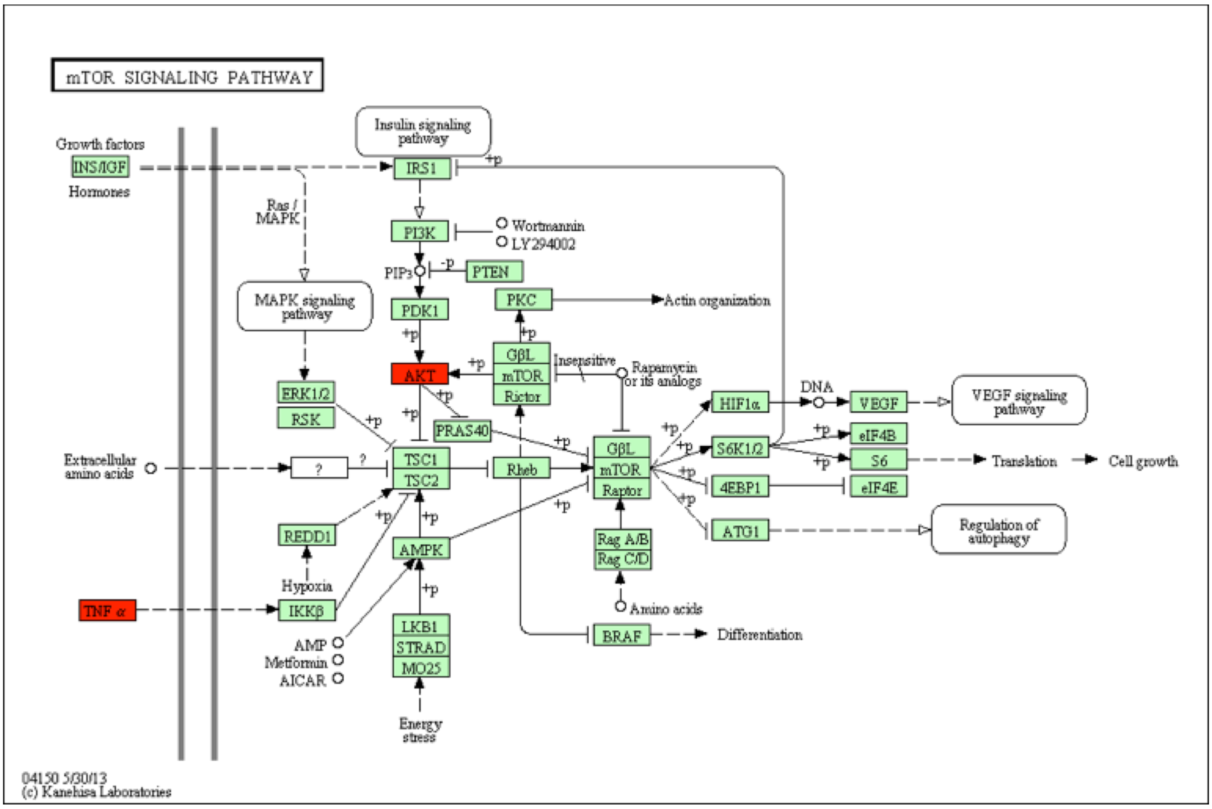

Figure S3. Two candidate genes for schizophrenia from the TDT sample (in red) mapped by KEGG to the mTOR signaling pathway are shown as an example of the data from KEGG employed for constructing the energy-delivering pathway (see Figs. 1 and 2).

Figure S4. KEGG's Hypoxia-Inducible Factor (HIF) pathway

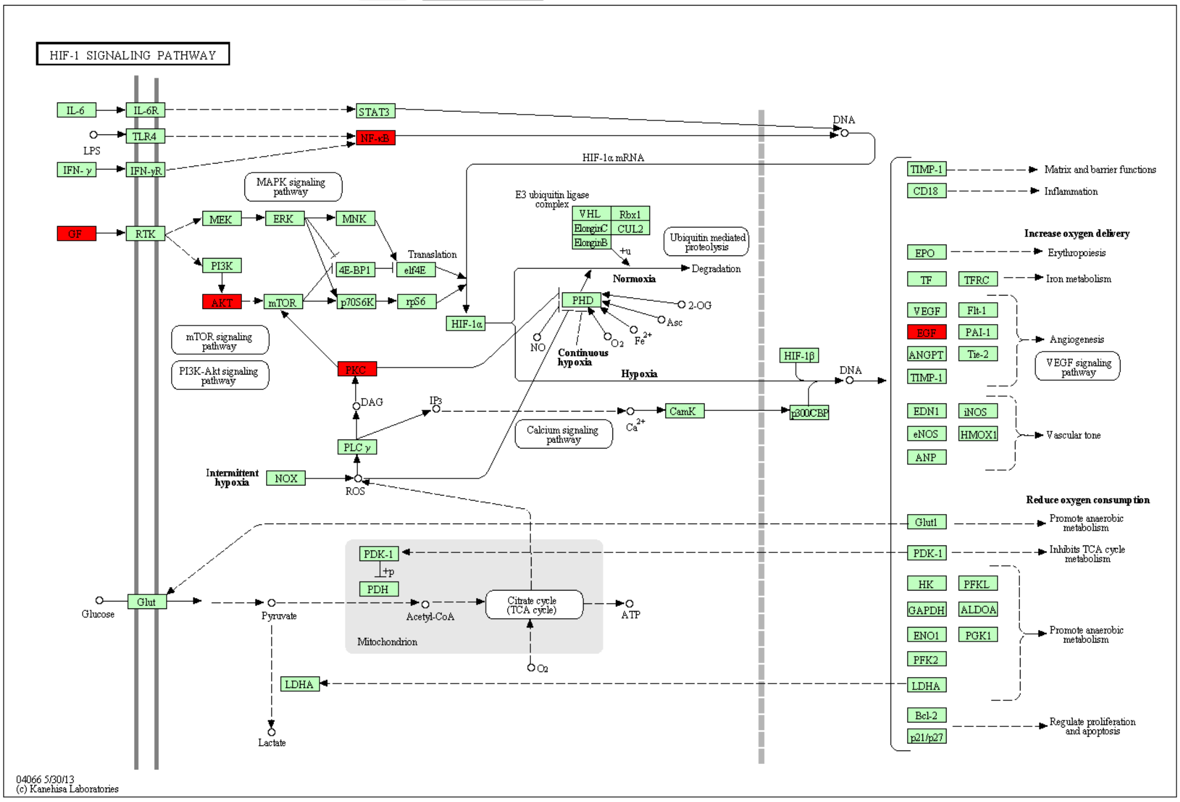

Figure S4. Five schizophrenia-associated genes mapped by KEGG to the Hypoxia-Inducible Factor (HIF) signaling pathway. The combined sample of 345 schizophrenia-associated genes was used to obtain this figure from KEGG.

Figure S5. KEGG's Vascular Smooth Muscle Contraction (VSMC) pathway

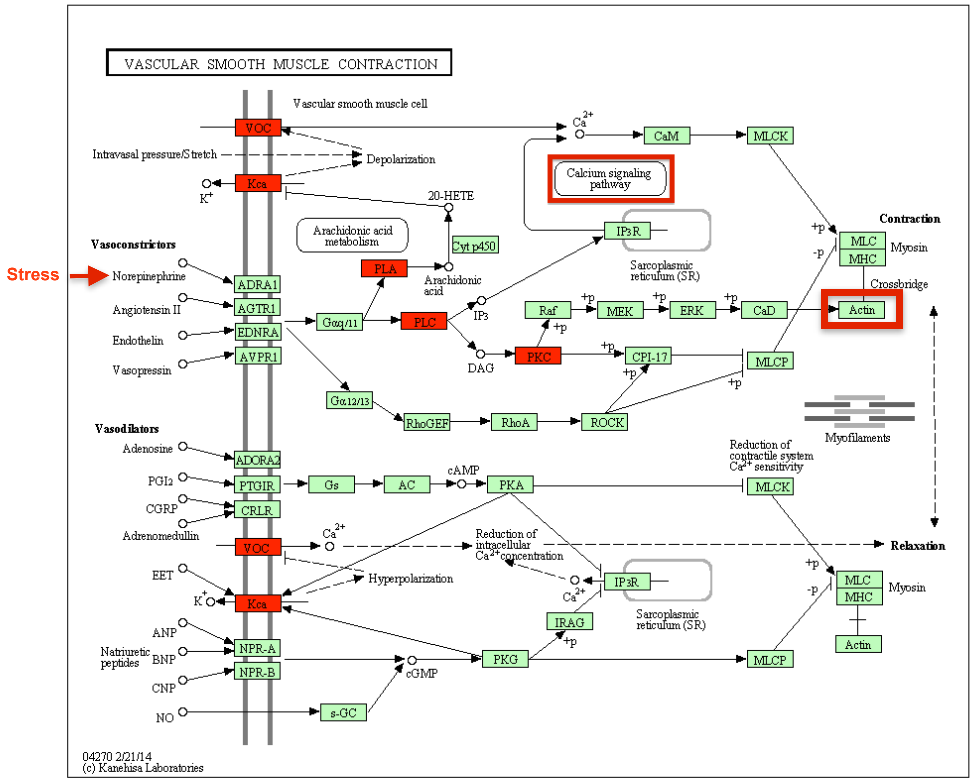

Figure S5. Five schizophrenia-associated genes were mapped by KEGG to the VSMC pathway. The combined sample was used for this Figure. In addition, actin was marked, because Fromer et al. (2014) and Zhao et al. (2014) found evidence for the involvement of actin in schizophrenia[293,294]. Furthermore, the polymerisation status of the submembranous actin web in vascular endothelium determines the activity of eNOS and the release of NO[295] (see also Fig. 1–2). In addition, a role for calcium signaling genes in schizophrenia was reported in the 2014 GWAS by the SWGPGC[5] and has recently been emphasized by Tansey et al. (2015)[296].

PANTHER pathway analysis

The PANTHER (**P**rotein **AN**alysis **TH**rough **E**volutionary **R**elationships) Classification System[297] at (http://www.pantherdb.org) was employed for pathway analysis of the discovery and the combined sample. The results of the latter are shown in Table S13. They involved the dopamine receptor mediated signaling pathway, adrenaline and noradrenaline biosynthesis, EGF receptor signaling pathway, 5HT2 type receptor mediated signaling pathway, nicotinic acetylcholine receptor signaling pathway. P values are Bonferroni corrected for multiple testing.

Table S13. Results of pathway analysis by PANTHER

Displaying only results with P<0.05; click here to display all results

	Homo sapiens (REF)	Client Text Box Input				
PANTHER Pathways	#	#	expected	Fold Enrichment	+/-	Δ P value
Unclassified	19446	16	33.89	.47	-	0.00E00
Dopamine receptor mediated signaling pathway	57	6	.10	> 5	+	1.26E-07
Adrenaline and noradrenaline biosynthesis	28	3	.05	> 5	+	2.66E-03
EGF receptor signaling pathway	123	4	.21	> 5	+	9.88E-03
5HT2 type receptor mediated signaling pathway	50	3	.09	> 5	+	1.48E-02
Nicotinic acetylcholine receptor signaling pathway	74	3	.13	> 5	+	4.65E-02

Genetic disease association analysis by DAVID

The discovery and the combined gene samples described in this article were also analysed genetic disease associations by the **D**atabase for **A**nnotation, **V**isualization and **I**ntegrated **D**iscovery (DAVID)[298] at (http://david.abcc.ncifcrf.gov/). Table S14 shows some of the results.

Table S14. DAVID’s genetic associations with diseases

Rerun Using Options | Create Sublist

22 chart records — Download File

Sublist	Category	Term	RT	Genes	Count	%	P-Value	Bonferroni	Fisher Exact
	GENETIC_ASSOCIATION_DB_DISEASE	schizophrenia	RT		37	92,5	7,0E-37	6,8E-34	6,1E-38
	GENETIC_ASSOCIATION_DB_DISEASE	bipolar disorder	RT		14	35,0	1,1E-10	1,1E-7	9,6E-12
	GENETIC_ASSOCIATION_DB_DISEASE	cognitive function	RT		10	25,0	4,9E-11	4,8E-8	1,4E-12
	GENETIC_ASSOCIATION_DB_DISEASE	alcohol abuse	RT		10	25,0	7,2E-10	7,0E-7	3,0E-11
	GENETIC_ASSOCIATION_DB_DISEASE	Parkinson's disease	RT		10	25,0	2,2E-5	2,1E-2	3,4E-6
	GENETIC_ASSOCIATION_DB_DISEASE	smoking behavior	RT		9	22,5	6,7E-9	6,5E-6	2,7E-10
	GENETIC_ASSOCIATION_DB_DISEASE	depression	RT		9	22,5	1,0E-8	1,0E-5	4,6E-10
	GENETIC_ASSOCIATION_DB_DISEASE	alcoholism	RT		9	22,5	8,3E-8	8,1E-5	4,9E-9
	GENETIC_ASSOCIATION_DB_DISEASE	attention deficit disorder conduct disorder oppositional defiant disorder	RT		9	22,5	2,8E-7	2,7E-4	1,9E-8
	GENETIC_ASSOCIATION_DB_DISEASE	methamphetamine abuse	RT		8	20,0	2,4E-10	2,3E-7	3,4E-12
	GENETIC_ASSOCIATION_DB_DISEASE	heroin abuse	RT		8	20,0	4,0E-10	3,9E-7	6,3E-12
	GENETIC_ASSOCIATION_DB_DISEASE	attention deficit hyperactivity disorder	RT		8	20,0	3,1E-7	3,0E-4	1,6E-8
	GENETIC_ASSOCIATION_DB_DISEASE	Alzheimer's Disease	RT		8	20,0	2,3E-2	1,0E0	8,2E-3
	GENETIC_ASSOCIATION_DB_DISEASE	Tourette syndrome	RT		7	17,5	1,6E-8	1,6E-5	2,9E-10
	GENETIC_ASSOCIATION_DB_DISEASE	tardive dyskinesia	RT		7	17,5	3,3E-7	3,2E-4	1,1E-8
	GENETIC_ASSOCIATION_DB_DISEASE	personality traits	RT		7	17,5	4,2E-7	4,1E-4	1,5E-8
	GENETIC_ASSOCIATION_DB_DISEASE	migraine	RT		7	17,5	3,9E-6	3,8E-3	2,2E-7
	GENETIC_ASSOCIATION_DB_DISEASE	depressive disorder, major	RT		7	17,5	1,4E-5	1,4E-2	1,0E-6
	GENETIC_ASSOCIATION_DB_DISEASE	obsessive compulsive disorder	RT		6	15,0	5,7E-7	5,5E-4	1,2E-8
	GENETIC_ASSOCIATION_DB_DISEASE	Anorexia Nervosa	RT		6	15,0	1,6E-6	1,5E-3	4,3E-8
	GENETIC_ASSOCIATION_DB_DISEASE	schizophrenia; bipolar disorder	RT		6	15,0	2,8E-6	2,7E-3	8,8E-8
	GENETIC_ASSOCIATION_DB_DISEASE	bipolar disorder schizophrenia	RT		6	15,0	3,6E-6	3,5E-3	1,2E-7

2 gene(s) from your list are not in the output.

Legend for Table S14. Functional annotation of the discovery sample by DAVID's genetic association database. Interestingly, seven of the candidate genes for schizophrenia are also involved in migraine (see above).

Gene set analysis

All genes from other species were transformed into human orthologous genes and identified by Entrez Gene IDs. The association between schizophrenia-associated genes and functional gene sets were computed by using the intersect function of the programming language R (version 3.0.2, platform: x86_64-apple-darwin10.8.0, 64-bit)[299].

Supplementary statistics information

Genome resampling test

Homo sapiens' complete list of genes was downloaded on April 8, 2011 from the Entrez Gene database maintained by the National Center for Biotechnology Information (NCBI) (http://www.ncbi.nlm.nih.gov/gene). It had 45 386 entries.

To improve the power (i.e. the chances of obtaining an intersection of randomly drawn genes from the database with functional gene sets), the list was curated by deleting all genes with unknown function from the Entrez Gene database. Using the Unix stream editor SED, genes with the following key words were removed: pseudogene, hypothetical LOC, hypothetical protein, pseudo, miscRNA, readthrough, read-through, open reading frame, deletion syndrome, duplication syndrome, triplication syndrome, unknown, uncharacterised protein, putative uncharacterised protein and repeat sequence. Next, all candidate genes for schizophrenia and functional gene sets were added and duplicates removed to ensure that all genes under investigation are represented equally among the constructed representation of the Human Genome and have a chance of being drawn during genomic resampling. The final modified list of Entrez Genes was comprised of 21 012 human genes mostly of known function.

The number of intersections between schizophrenia-associated genes and functional gene sets was determined by the intersect function implemented in R[299]. A genomic resampling procedure was employed to obtain estimates of the expected numbers of intersecting genes by drawing the same number of genes as the candidate genes 1 million times at random (with replacement) from the representation of the Human Genome described above and determining the intersection of the random genes with the functional gene set. The resampling method has the avantage of being independent of the distribution assumption, but due to computational restrictions significance can only be computed up to a threshold of $p \geq$ 10E-6. Within this limit imposed by computational restriction, the empiciral p-values were identical to the nominal p-values from Fisher's Exact Test.

RESULTS

Putative schizophrenia pathway

The stress-induced increase of cortisol and desoxycorticosterone (DOC)[300] has an inhibitory influence on the expression of glucose transporters[301], brain metabolism[302], and serotonin uptake[303]. Furthermore, stress induces the release of adrenaline into the circulation and of noradrenaline and dopamine in the prefrontal cortex (reviewed in[304]); these are neurotransmitters known for their vasoconstrictive effects[69,303] (reviewed in[305]). Magnitude and duration of the signals of these neurotransmitters is primarily influenced by their plasma membrane transporters (e.g., DAT1, NAT1 and 5-HTT) (reviewed in[306]). The expression of these transporters at the cell surface depends on the activity of the PI3K/Akt pathway and its stimulation by insulin and insulin-like growth factor 1 (IGF-1)[307].

Numerous studies have shown that growth factors, cytokines and hormones (such as insulin, IGF-1, EGF, prolactin, estrogens and erythropoietin) have a positive effect on Akt, thereby probably reducing the vasoconstrictive impact of stress via Akt's positive influence on DAT1[264], NAT1[308] and eNOS[309]. At low concentrations, testosterone exerts an activating effect on Akt, but an inhibitory effect at high concentrations[310]. In addition, prolactin activates Akt[311] suggesting that the hyperprolactinemia caused by typical antipsychotics is likely to have vasodilatatory and ischemia-protective effects (Fig. 2).

In summary, cerebral blood flow and energy supply depend on growth factors, hormones and genes involved in the PI3K–Akt–mTOR pathway. Activation of this pathway also protects tissue from ischemia by influencing the protein synthesis of the hypoxia-inducing factor (HIF)[312], which subsequently induces the translation of more than 70 proteins in order to increase blood flow, cellular survival and alternative energy production from lactate.

Discovery sample

Table S15. Intersection between functional gene sets and candidate genes for schizophrenia obtained from TDT studies.

Functions	*N*	SZ *N*	E (N)	O (N)	E (%)	O (%)	RF	CI	Nominal $p \leq$	Bonferroni corrected $p \leq$
VIRND	6409	41	13	28	30.4	68.3	2.3	22–41	6.8E-07	1.3e-05 **
VI	4213	41	8	25	20.1	61.0	3.0	19–41	1.2E-08	2.3e-07 ***
V	3249	41	6	20	15.4	48.8	3.2	14–41	5.9E-07	1.1e-05 **
PV	253	41	1	11	1.2	26.8	22.3	6–41	1.7E-12	3.2e-11 ***
I	1673	41	3	15	8.0	36.6	4.6	1041	2.8E-07	5.3e-06 **
R	159	41	0	11	0.7	26.8	35.5	6–41	8.5E-15	1.6e-13 ***
Repair	3319	41	6	23	15.8	56.1	3.6	17-41	3.7E-09	7.0e-08 ***
ND	3211	41	6	16	15.3	39.0	2.6	11–41	1.9E-04	3.6e-03 *
SY	1977	41	4	19	9.4	46.3	4.9	13–41	9.9E-10	1.9e-08 ***
VI x ND	1051	41	2	13	5.0	31.7	6.3	8–41	5.4E-08	1.0e-06 ***
VI x Repair	1124	41	2	20	5.3	48.8	9.1	14–41	2.8E-15	5.3e-14 ***
V x ND	784	41	2	11	3.7	26.8	7.2	6–41	2.0E-07	3.8e-06 **
V x Repair	845	41	2	18	4.1	43.9	10.9	13–41	5.3E-15	1.0e-13 ***
I x ND	523	41	1	8	2.5	19.5	7.9	4–41	6.4E-06	1.2e-04 **
I x Repair	569	41	1	12	2.7	29.3	10.8	7–41	5.2E-10	9.9e-09 ***
VI -ND	3163	41	6	12	15.1	29.3	1.9	7–41	0.02 n.s.	0.38 n.s.
VI -SY	3273	41	6	9	15.5	22.0	1.4	5–41	0.18 n.s	1.0 n.s.
ND -VI	2161	41	4	3	10.3	7.3	0.7	1–41	0.81 n.s	1.0 n.s.
SY -VI	1037	41	2	3	4.9	7.3	1.5	1–41	0.33 n.s	1.0 n.s.

Legend for Table S15. CI, 95 percent confidence intervals expressed as number of identical genes; E, Expected number and percentage of intersecting genes by chance; I, ischemia genes; Minus sign (-), overlapping genes removed; ND, neurodevelopmental genes; O, Observed number or percentage of intersecting genes; PV, perivascular nerve genes; R, post-ischemic repair genes; Repair, R and ND genes combined because ND

genes are involved in post-ischemic repair[120,127]; RF, representation factor, i.e., the number of intersecting genes divided by the expected number of intersecting genes drawn from two independent groups. RF > 1 indicates an overrepresentation of genes; SY, synaptic genes; SZ, schizophrenia-associated genes; x, indicate interacting genes; VI, vascular and ischemia genes of the brain combined. VIRND, all genes involved in ischemia combined, i.e., V, I, R and ND genes. Levels of significance (Bonferroni corrected): * indicates $p \leq$ 1.0E-02; ** indicates $p \leq$ 1.0E-03; *** indicates $p \leq p \leq$ 1.0E-06.

Replication sample #1

Table S16. Intersection between functional gene sets and candidate genes for schizophrenia obtained from case-control studies (CC).

Functions	*N*	SZ *N*	E (N)	O (N)	E (%)	O (%)	RF	CI	Nominal *p* ≤	Bonferroni corrected *p* ≤
VIRND	6409	58	18	47	30.6	81.0	2.7	41–58	2.4E-15	5.0e-14 ***
VI	4213	58	12	43	20.1	74.1	3.7	36-58	2.2E-16	4.6e-15 ***
V	3249	58	9	36	15.4	62.1	4.0	29-58	9.0E-16	1.9e-14 ***
PV	253	58	1	19	1.2	32.7	27.2	13-58	2.2E-16	4.6e-15 ***
I	1673	58	5	28	8.0	48.3	6.1	21-58	4.5E-16	9.5e-15 ***
R	159	58	0	19	0.7	32.8	43.3	13–58	2.2E-16	4.6e-15 ***
Repair	3319	58	9	29	15.8	50.0	3.2	22–58	1.3E-09	2.7e-08 ***
ND	3211	58	9	16	15.3	27.6	1.8	10-58	0.01 n.s.	2.1e-01 n.s.
SY	1977	58	6	23	9.4	39.7	4.2	16-58	8.0E-10	1.7e-08 ***
VI x ND	1051	58	3	13	5.0	22.4	4.5	8–58	4.4E-06	9.2e-05 **
VI x Repair	1124	58	3	25	5.3	43.1	8.1	19–58	2.2E-16	4.6e-15 ***
V x ND	784	58	2	12	3.7	20.7	5.6	7–58	1.2E-06	2.5e-05 **
V x Repair	845	58	2	24	4.0	41.4	10.3	18–58	2.2E-16	4.6e-15 ***
I x ND	523	58	1	8	2.5	13.8	5.6	4–58	8.8E-05	1.8e-03 *
I x Repair	569	58	2	16	2.7	27.6	10.2	11–58	1.9E-12	4.0e-11 ***
VI -ND	3163	58	8	30	15.0	51.7	3.4	23-58	7.6E-11	1.6e-09 ***
VI -SY	3273	58	9	21	15.5	36.2	2.3	15-58	1.0E-04	2.1e-03 *
I -ND	1151	58	3	20	5.5	34.5	6.3	14-58	1.4E-11	2.9e-10 ***
I -SY	1180	58	3	15	5.6	25.9	4.6	10-58	5.1E-07	1.1e-05 **
ND -VI	2161	58	6	3	10.2	5.2	0.5	1–58	0.96 n.s.	1.0 n.s.
SY -VI	1037	58	3	1	4.9	1.7	0.3	1–58	0.95 n.s.	1.0 n.s.

Legend for Table S16. Abbreviations are explained at Table S15. Levels of significance (Bonferroni corrected): * indicates $p \leq$ 1.0E-02; ** indicates $p \leq$ 1.0E-03; *** indicates p $\leq p \leq$ 1.0E-06.

Replication sample #2

Table S17. Intersection of the schizophrenia gene set obtained from the NHGRI catalog with functional gene sets.

Functions	*N*	SZ *N*	E (N)	O (N)	E (%)	O (%)	RF	CI	Nominal $p \leq$	Bonferroni corrected $p \leq$
VIRND	6409	164	50	76	30.5	46.3	1.5	65–164	6.0E-06	1.1e-04 **
VI	4213	164	33	44	20.0	26.8	1.3	35–164	0.02	0.38 n.s.
V	3249	164	25	37	15.5	22.6	1.5	28–164	0.01	0.19 n.s.
PV	253	164	2	7	1.2	4.3	3.5	3–164	0.004	7.6e-02 n.s.
I	1673	164	13	16	7.9	9.8	1.2	10–164	0.23	1.0 n.s.
R	159	164	1	6	0.7	3.7	4.8	3–164	0.001	1.9e-02 n.s.
Repair	3319	164	26	50	15.8	30.5	1.9	40–164	9.2E-07	1.7e-05 **
ND	3211	164	25	46	15.3	28.0	1.8	37–164	2.1E-05	4.0e-04 **
SY	1977	164	15	31	9.4	18.9	2.0	23–164	1.4E-04	2.7e-03 *
VI x ND	1051	164	8	15	5.0	9.1	1.8	9–164	0.02	0.38 n.s.
VI x Repair	1124	164	9	18	5.3	11.0	2.1	12–164	0.003	5.7e-02 n.s.
V x ND	784	164	6	13	3.7	7.9	2.1	8–164	0.007	0.13 n.s.
V x Repair	845	164	7	15	4.0	9.1	2.3	9–164	0.003	5.7e-02
I x ND	523	164	4	8	2.5	4.9	2.0	4–164	0.05	0.95 n.s.
I x Repair	569	164	4	10	2.7	6.1	2.3	5–164	0.01	0.19 n.s.
VI -ND	3163	164	25	29	15.1	17.7	1.2	21–164	0.20	1.0 n.s.
VI -SY	3273	164	25	29	15.6	17.7	1.1	21–164	0.26	1.0 n.s.
ND -VI	2161	164	17	31	10.2	18.9	3.7	23–164	6.4E-04	1.2e-02 n.s.
SY -VI	1037	164	8	16	4.9	9.8	2.0	10–164	8.0E-03	0.15 n.s.

Legend for Table S17. Abbreviations are explained at Table S15. Levels of significance (Bonferroni corrected): * indicates $p \leq$ 1.0E-02; ** indicates $p \leq$ 1.0E-03; *** indicates $p \leq$ $p \leq$ 1.0E-06.

Replication sample #3

Table S18. Intersection of the schizophrenia gene set developed by Ayalew et al.[4] with functional gene sets.

Functions	*N*	SZ *N*	E (N)	O (N)	E (%)	O (%)	RF	CI	Nominal *p* ≤	Bonferroni corrected *p* ≤
VIRND	6409	42	13	36	30.5	85.7	2.8	31–42	1.6E-13	3.0e-12 ***
VI	4213	42	8.5	31	20.2	73.8	3.7	25–42	1.2E-13	2.3e-12 ***
V	3249	42	6	24	15.5	57.1	3.7	18–42	6.9E-10	1.3e-08 ***
PV	253	42	1	12	1.2	28.6	23.7	1-42	7.4E-14	1.4e-12 ***
I	1673	42	3.4	22	8.0	52.4	6.6	16–42	8.4E-14	1.6e-12 ***
R	159	42	0	11	0.7	26.2	34.6	6–42	1.2E-14	2.3e-13 ***
Repair	3319	42	7	29	15.8	69.0	4.4	23–42	1.6E-14	3.0e-13 ***
ND	3211	42	6.5	26	15.4	61.9	4.1	20–42	9.9E-12	1.9e-10 ***
SY	1977	42	4.0	29	9.5	69.0	7.3	23–42	2.2E-16	4.2e-15 ***
VI x ND	1051	42	2	21	5.0	50.0	10.0	15–42	2.2E-16	4.2e-15 ***
VI x Repair	1124	42	2	24	5.3	57.1	10.7	18–42	2.2E-16	4.2e-15 ***
V x ND	784	42	2	16	3.8	38.1	10.2	11–42	8.0E-13	1.5e-11 ***
V x Repair	845	42	2	19	4.1	45.2	11.3	14–42	5.6E-16	1.1e-14 ***
I x ND	523	42	1	14	2.4	33.3	13.4	9–42	8.0E-13	1.5e-11 ***
I x Repair	569	42	1	17	2.7	40.5	15.0	12–42	2.4E-16	4.6e-15 ***
VI -ND	3163	42	6.4	10	15.2	23.8	1.6	5–42	0.09 n	1.0 n.s.
VI -SY	3273	42	6.6	7	15.7	16.7	1.1	3–42	0.50	1.0 n.s.
ND -VI	2161	42	4.4	5	10.4	11.9	1.2	2–42	0.44	1.0 n.s.
SY -VI	1037	42	2.1	5	5.0	11.9	2.4	2–42	0.06	1.0 n.s.

Abbreviations are explained at Table S15. Levels of significance (Bonferroni corrected): * indicates $p \leq$ 1.0E-02; ** indicates $p \leq$ 1.0E-03; *** indicates $p \leq p \leq$ 1.0E-06.

Replication sample #4 (all genes within range)

Table S19. Intersection of all the 343 genes within range of 108 genome-wide significant loci reported in 2014 by the SWGPGC in the largest GWAS of schizophrenia to date.

Functions	*N*	SZ *N*	E (N)	O (N)	E (%)	O (%)	RF	CI	Nominal $p \leq$	Bonferroni corrected $p \leq$
VIRND	6409	343	105	154	30.5	44.9	1.5	139–343	5.2E-09	9.9e-08 ***
VI	4213	343	69	97	20.1	28.3	1.4	83–343	1.7E-04	3.2e-03 *
V	3249	343	53	76	15.5	22.2	3.5	63–343	6.8E-04	1.3e-02 n.s.
PV	253	343	4	12	1.2	3.5	2.9	7–343	0.001	1.9e-02 n.s.
I	1673	343	27	46	8.0	13.4	1.7	36–343	3.8E-04	7.2e-03 *
R	159	343	3	7	0.8	2.0	2.7	3–343	0.01	0.19 n.s.
Repair	3319	343	54	95	15.8	27.7	1.8	81–343	7.4E-09	1.4e-07 ***
ND	3211	343	52	91	15.2	26.5	1.7	78–343	5.9E-08	1.1e-06 ***
SY	1977	343	32	49	9.4	14.3	1.5	39–343	0.002	3.8e-02 n.s.
VI x ND	1051	343	17	35	5.0	10.2	2.0	26–343	4.4E-05	8.4e-04 **
VI x Repair	1124	343	18	38	5..3	11.1	2.1	29–343	1.5E-05	2.8e-04 **
V x ND	784	343	13	28	3.7	8.2	2.2	20–343	8.1E-05	1.5e-03 *
V x Repair	845	343	14	31	4.0	9.0	2.3	23–343	2.0E-05	3.8e-04 **
I x ND	523	343	9	22	2.5	6.4	2.6	15–343	4.6E-05	8.7e-04 **
I x Repair	569	343	9	23	2.7	6.7	2.5	16–343	5.8E-05	1.1e-03 *
VI -ND	3163	343	52	62	15.0	18.1	1.2	50–343	0.07	1.0 n.s.
VI -SY	3273	343	53	67	15.6	19.5	1.3	55–343	0.03	0.57 n.s.
ND -VI	2161	343	35	56	10.3	16.3	1.6	45–343	3.7E-04	7.0e-03 *
SY -VI	1037	343	17	19	4.9	5.5	1.1	12–343	0.34	1.0 n.s.

Legend for Table S19. Abbreviations are explained at Table S15. SZ, all the genes within range of the 108 genome-wide significant loci listed in column 5 of supplementary Table 3 of the large GWAS of schizophrenia by the SWGPGC 2014[5]. Levels of significance (Bonferroni corrected): * indicates $p \leq$ 1.0E-02; ** indicates $p \leq$ 1.0E-03; *** indicates $p \leq p \leq$ 1.0E-06.

Replication sample #4 (genes assigned by proximity)

Table S20. Intersection between functional gene sets and 111 genes assigned by proximity to 108 genome-wide significant loci from the largest GWAS of schizophrenia to date.

**Functions**	*N*	**SZ** *N*	**E (N)**	**O (N)**	**E (%)**	**O (%)**	**RF**	**CI**	**Nominal** *p* ≤	**Bonferroni corrected** *p* ≤
VIRND	6409	111	34	58	30.5	52.3	1.7	49–111	1.4E-06	2.7e-05 **
VI	4213	111	22	40	20.0	36.0	1.8	32–111	6.7E-05	1.3e-03 *
V	3249	111	17	33	15.4	29.7	1.9	25–111	1.0E-04	1.9e-03 *
PV	253	111	1	8	1.2	7.2	6.0	4–111	6.5E-05	1.2e-03 *
I	1673	111	9	19	8.0	17.1	2.1	13–111	0.001	1.9e-02 n.s.
R	159	111	1	6	0.8	5.4	7.1	3–111	2.0E-04	3.8e-03 *
Repair	3319	111	18	40	15.8	36.0	2.3	32–111	1.5E-07	2.8e-06 **
ND	3211	111	17	37	15.3	33.3	2.2	29–111	1.8E-06	3.4e-05 **
SY	1977	111	10	18	9.4	16.2	1.7	12–111	0.02	0.38 n.s.
VI x ND	1051	111	6	19	5.0	17.1	3.4	13–111	2.4E-06	4.6e-05 **
VI x Repair	1124	111	6	22	5.3	19.8	3.7	15–111	8.4E-08	1.6e-06 **
V x ND	784	111	4	15	3.7	13.5	3.6	9–111	1.6E-05	3.0e-04 *
V x Repair	845	111	4	18	4.0	16.2	4.0	12–111	4.4E-07	8.4e-06 **
I x ND	523	111	3	13	2.5	11.7	4.7	8–111	3.9E-06	7.4e-05 **
I x Repair	569	111	3	14	2.7	12.6	4.7	9–111	1.8E-06	3.4e-05 **
VI -ND	3163	111	17	21	15.1	18.9	1.2	15–111	0.16	1.0 n.s.
VI -SY	3273	111	17	27	17.3	24.3	1.6	20–111	0.01	0.19 n.s.
ND -VI	2161	111	11	18	10.3	16.2	1.6	12–111	0.04	0.76 n.s.
SY -VI	1037	111	5	5	4.9	4.5	0.9	2–111	0.65	1.0 n.s.

Abbreviations are explained at Table S15. Levels of significance (Bonferroni corrected): * indicates $p \leq$ 1.0E-02; ** indicates $p \leq$ 1.0E-03; *** indicates $p \leq p \leq$ 1.0E-06.

Postmortem studies of schizophrenia

Table S21. Intersection of functional gene sets with genes differentially expressed in postmortem brains of schizophrenic patients.

Functional Gene Set		Genes Differentially Expressed in Postmortem Brains of Schizophrenic Patients								
		SZ All			SZ Up			SZ Down		
	N	*N*	RF	Bonferroni corrected *p* ≤	*N*	RF	Bonferroni corrected *p* ≤	*N*	RF	Bonferroni corrected *p* ≤
VIRND	6409	113	1.3	0.03 *	31	1.6	0.02*	82	1.2	1.0 n.s.
VI	4213	113	1.2	0.95 n.s.	31	1.7	0.13 n.s.	82	1.1	1.0 n.s.
V	3249	113	1.1	1.0 n.s.	31	1.6	1.0 n.s.	82	1.0	1.0 n.s.
I	1673	113	1.4	1.0 n.s.	31	1.8	1.0 n.s.	82	1.2	1.0 n.s.
R	159	113	0.7	1.0 n.s.	31	2.7	1.0 n.s.	82	0.0	1.0 n.s.
Repair	3319	113	1.7	3.4e-04 **	31	1.7	0.57 n.s.	82	1.7	3.2e-03 **
ND	3211	113	1.8	1.3e-04 **	31	1.7	0.38 n.s.	82	1.8	1.5e-03 **
SY	1977	113	1.7	0.08 n.s.	31	0.6	1.0 n.s.	82	2.0	5.3e-03 **
VI x ND	1051	113	2.6	4.6e-04 ***	31	2.4	0.57 n.s.	82	2.6	4.0e-03 **
VI x Repair	1124	113	2.4	1.3e-03 **	31	2.3	0.76 n.s.	82	2.4	8.7e-03 **
V x ND	784	113	2.2	0.06 n.s.	31	1.6	1.0 n.s.	82	2.5	0.06 n.s.
V x Repair	845	113	2.1	0.11 n.s.	31	1.5	1.0 n.s.	82	2.3	0.11 n.s.
I x ND	523	113	2.5	0.10 n.s.	31	2.4	1.0 n.s.	82	2.5	0.38 n.s.
I x Repair	569	113	2.3	0.17 n.s.	31	2.2	1.0 n.s.	82	2.3	0.57 n.s.
VI -ND	3163	113	0.8	1.0 n.s.	31	1.5	1.0 n.s.	82	0.6	0.19 n.s.
VI -SY	3273	113	1.2	1.0 n.s.	31	2.1	0.02 *	82	0.8	1.0 n.s.
ND -VI	2161	113	1.4	0.76 n.s.	31	1.4	1.0 n.s.	82	1.4	1.0 n.s.
ND -I	2689	113	1.7	5.9e-03 **	31	1.6	1.0 n.s.	82	1.7	0.06 n.s.
SY -VI	1037	113	1.8	0.19 n.s.	31	0.8	1.0 n.s.	82	2.2	0.10 n.s.

Abbreviations are explained at Table S15. The differentially expressed gene samples were obtained from the large combined cohort by Mistry et al.[313]. Because of the small sample

sizes, the p-value for significance was increased to 0.05 (Bonferroni corrected): * indicates $p \le 0.05$; ** indicates $p \le 0.01$; *** indicates $p \le p \le 1.0e\text{-}03$

Overview of significant findings

Table S22. Overview of significant findings.

**Sample**	**VIRND**	**VI x ND**	**VI x Repair**	**VI**	**I**	**R**	**Repair**	**ND**	**SY**	**ND -VI**	**VI - ND**	**VI - SY**
**Candidate-gene association studies**												
**Discovery**	**	***	***	***	**	***	***	*	***			
**Replication #1**	***	**	***	***	***	***	***		***		***	*
**GWA studies**												
**Replication #2**	**						**	**	*			
**Replication #3**	***	***	***	***	***	***	***	***	***			
**Replication #4**												
(Proximity)	**	**	**	*		*	**	**				
(Within range)	***	**	**	*	*		***	***		*		
**All combined**	***	***	***	***	***	***	***	***	***		**	
**Postmortem gene expression studies of prefrontal cortex**												
All DE genes	*	***	**				**	**				
Up-regulated	*											*
Down-regulated		**	**				**	**	**			

Legend for Table S22. Significant findings from Tables S15–S21 and Table 1 are shown. The number of asterisks refers to Bonferroni corrected *p*-values as given in these Tables.

- minus sign, overlapping genes removed;
DE, differentially expressed genes;
I, genes induced by cerebral ischemia;
ND, neurodevelopmental genes;
proximity, genes assigned by proximity to index SNP;
R, post-ischemic repair genes;
Repair, R and ND genes combined, because ND genes are involved in post-ischemic repair[120,127];
SY, synaptic genes;
VI, vascular-ischemia genes;
VIRND, all genes involved in ischemia, i.e., V, I, R and ND genes;
within range, all genes within range of index SNP;
x, overlapping, i.e., interacting genes.

Quasi experimental study

Figure S6. Quasi-experimental impairment of components of the candidate pathway

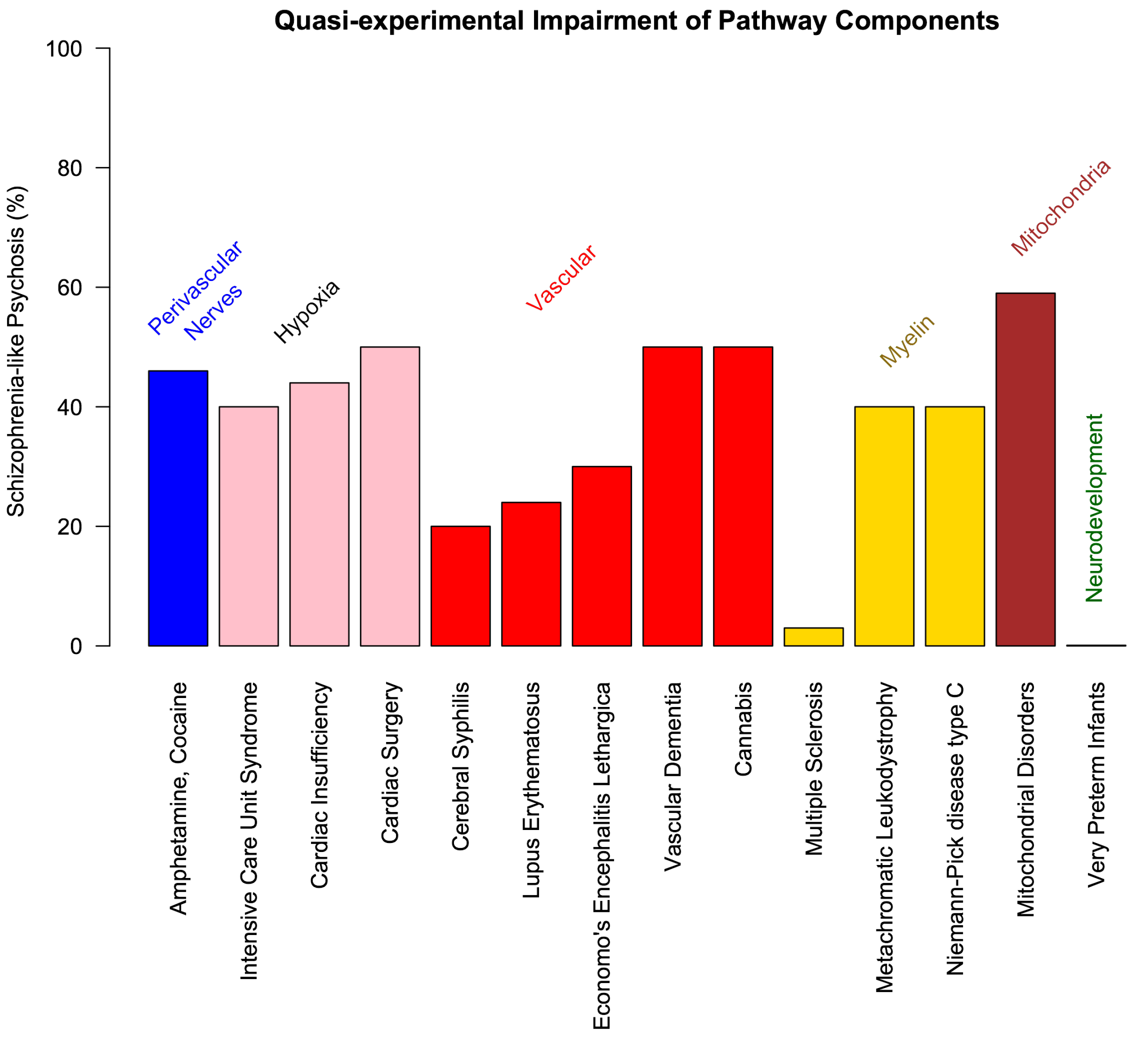

Figure S6. Results of quasi-experimental impairment of components of the postulated schizophrenia pathway. Disturbances of all components of the energy-supply pathway, i.e. perivascular nerves, oxygen, microvessels, oligodendrocytes, and mitochondria (see Fig. 1 in article), appear to produce a high percentage of schizophrenia-like symptoms. Multiple sclerosis (MS) is the exception, which might be due to the localized damage of myelin in MS. Very preterm birth seems to impair neurodevelopment independent of an increase in risk for ischemic disorders[314,315]. It causes high rates of neurodevelopmental disabilities from 25% to 50% such as cerebral palsy (5% to 15%)[316], but only 0.05 % (495 of 1 022 431) of term births compared to 0.1 % (6 of 5125) of very preterm births (gestational age < 32 weeks) developed later a non-affective psychosis[317]. For more data and references, see also supplementary Table S23–S24.

Table S23. Quasi-experimental disturbance of candidate schizophrenia pathway as independent variables and the production of schizophrenia-like symptoms.

**Putative schizophrenia pathway**	**Evidence for mild ischemia**	**Schizophrenia-like symptoms§**	**Max. Rate**
Hypoxia			
Cardiac Insufficiency	Insufficient tissue oxygenation, mild ischemia	Cardiac psychosis, paranoid-hallucinatory syndromes in 44% of acute cardiogenic psychosis[318].	44%
Cardiac Surgery	Measures of hypoxia and cerebral hypoperfusion predict postoperative neuropsychiatric disorders[319,320]	Paranoid-hallucinatory syndromes[321,322], psychoses, delirium, and cognitive dysfunction are common following a lucid postoperative interval[323]. Neuropsychological dysfunction occurs frequently in 40% to 50%, up to 79% of patients[323].	50%
Intensive Care Unit Syndrome (ICUS)	ICU syndrome/delirium is associated with decreased anemia and extended times on the ventilator[324].	Hallucinations and delusions usually as part of delirium[325].	40%
Perivascular nerves			
Amphetamine (AMPH) and Cocaine	AMPH and cocaine cause a decrease in CBF[326], cerebral vasospasms[327,328] and ischemic as well as hemorrhagic strokes[267,329-331], probably via their action on DAT1, NAT1 and 5-HTT resulting in an increase of dopamine, noradrenaline and serotonin[332,333].	Paranoid state with auditory and visual hallucinations in chronic users[334,335] resembling schizophrenia[336-338]. Drug-induced psychosis has been reported in 8–46% of regular users of amphetamines[339].	46%
Anticholin-ergics	Reduced cortical perfusion, mainly in the frontal cortex[340].	Paranoid-hallucinatory psychosis[341,342], worsening of positive symptoms in schizophrenia[343].	
Traumatic Brain Injury (TBI)	TBI consistently damages cerebral perivascular nerves and impairs autoregulation of CBF[344].	Higher frequency of prior TBI in schizophrenia compared to other psychiatric disorders[345].	
Vascular component			

Vascular Dementia (VaD)	VaD results from ischemic injury or sustained mild ischemia[346,347].	In mild VaD, 37% of patients had hallucinations and 50% paranoid symptoms[348]. In VaD, 8.1% heard voices and 8.6% talked to people who were not there[349].	50%
Neuro-psychiatric Systemic Lupus Erythematosus (NPSLE)	Vasculitis is rare but vascular hyalinization, endothelial proliferation and perivascular gliosis are common[350]. Furthermore, small lesions in white matter (WM) (100%), diffuse WM abnormalities (43%) and cerebral infarction (29%)[351]. Cerebral hypoperfusion measured by SPECT is related to neuropsychiatric symptoms in NPSLE[352].	Psychosis has been reported in 5% of NPSLE[353] including schizophrenia-like psychosis[354-356]. In consultation psychiatry, 24% of NPSLE had schizophrenia or unclassified psychosis[354].	24%
Cerebral syphilis	Treponema pallidum invades - through the intercellular junctions of endothelial cells[357] into perivascular areas[358] causing perivasculitis, adhesion of leukocytes, endothelial cell abnormalities[359] and ischemic stroke as primary symptom in about 14% of neurosyphilis patients[360].	Schizophrenia-like psychoses have been reported in patients with general paresis from 3.5% up to 20% (reviewed in[361])	20%
Economo's Encephalitis Lethargica (EL)	Perivascular inflammation and infiltrates of lymphocytes within the Virchow-Robin spaces of small vessels in acute EL[362]. In 45% of cases, the cortex is affected[363].	Postencephalitic schizophrenia-like psychoses[364] in 15-30% of EL cases[365].	30%
Cannabis	Increase of CBF in acute cannabis use, but decrease of CBF in frontal cortex of chronic users[366].	Cannabis-induced psychosis shares genetic predisposition and many common symptoms with schizophrenia[367,368]. The maximum proportion of psychosis attributable to cannabis in psychosis-free subjects is higher than 50 percent[369].	50%
Oligodendro-cyte & myelin component			
Metachromatic	Bilateral fronto-temporal white	Adult onset psychosis in 25-	40%

leukodystrophy	matter is affected (reviewed in[370]).	40% (reviewed in[370]).	
Niemann–Pick disease type C	Callosal and periventricular white matter is affected (reviewed in[370]).	Adult-onset psychosis or dementia in 25-40% (reviewed in[370]).	40%
Multiple sclerosis (MS)	MS is an inflammatory-mediated demyelinating disease of the human brain (reviewed in[371]).	In 2% - 3% of MS patients, a psychosis develops (reviewed in[372]).	3%
Mitochondrial component			
Mitochondrial disorders	Symptoms of mitochondrial disorders can appear either in infancy or adulthood. Various organs can be affected including the brain with stroke-like episodes in MELAS (Mitochondrial encephalo-myopathy, lactic acidosis, stroke-like episodes) (reviewed in[373]).	Psychosis or psychotic features combined with mood disorders were diagnosed in 35 of 59 cases (59%) with a mitochondrial disorder (reviewed in[374]).	59%
Neuro-development			
Preterm infants	Very preterm infants (< 32 weeks of gestational age), have shown in follow-up studies high rates of neurodevelopmental disability with 5% to 15% having cerebral palsy, severe neurosensory impairment, and 25% to 50% having cognitive, behavioral, and social difficulties that impede progress in school[316].	Increased rate of psychiatric hospitalization for non-affective psychoses (i.e. schizophrenia and schizoaffective psychosis) from 0.05% (495 of 1022431 cases) for term birth to 0.1% (6 of 5125 cases) for very preterm infants (< 32 weeks of gestational age)[317].	0.1%

§ defined as hallucinations and/or delusions with or without disorientation

Experienced clinical psychiatrist will have observed, that the former, i.e., hallucinations and/or delusions with disorientation, correspond to the diagnosis of delirium and might demand an explanation for the lumping together of schizophrenia with delirium.

First, lumping and splitting of diagnostic categories are widely used in psychosis research (see Kraepelin's lumping of paranoia, hebephrenia and catatonia and his splitting of major psychoses into schizophrenia and manic depression or the current debate about the lumping of bipolar disorder and schizophrenia based on genomic findings[375,376].

Second, similarities between schizophrenia and neurodevelopmental disorders, such

as cerebral palsy, epilepsy, and mental retardation, have frequently been used to support the neurodevelopmental hypothesis[377,378]. See, e.g., page 401 of Weinberger's and Harrison's recent, excellent book on schizophrenia[379]: "In a sense, schizophrenia appears to be on a developmental continuum with other behavioral disorders that appear in childhood, including autism, intellectual disability, and epilepsy, arising perhaps from overlapping biological risk factors that may each have distinct covariants, but schizophrenia reflects the relatively least noise burden of this group of developmental disturbances[378]".

Third, the same reasoning may be applied to ischemia, with stroke on one end of the continuum, delirium and schizophrenia in the middle, hyperperfusion on the other end (see Fig. 4), and cerebral localization as covariant. Differences in severity or cerebral localization of ischemia might account for differences in disorientation. Consequently, an acute ischemic psychosis of known etiology would be diagnosed as delirium and an ischemic psychoses of unknown cause without disorientation as schizophrenia. For this discussion of hallucinations/delusions with disorientation, it is important to note that disorientation has also been found in some acute[380], as well as in chronic schizophrenic patients[381-384]. In regard to cerebral localization, isolated time orientation has been observed in 4 percent of patients with thalamic ischemia[385].

Table S24. Quasi-experimental neurodevelopmental disturbance

as independent variable and the production of schizophrenia-like symptoms.

Etiology of neurodevelop-mental disturbance	Evidence for neurodevelopmental disturbance	Schizophrenia-like symptoms§	Max. Rate
Obstetric complications (OBC)	After intrauterine and neonatal insults, the most common long-term outcome were developmental delay (59%), cognition and learning difficulties or cerebral palsy (21%), hearing impairment (20%) and visual impairment (18%) (meta-analysis[386]).	Since OBC is associated with the development of cardiovascular disorders[387,388] and stroke in adulthood[314,315,389,390] (reviewed in[391,392]), OBC cannot be considered here as proving that schizophrenia-like symptoms are caused by neurodevelopmental disturbance.	
Perinatal brain damage	A 1966 North Finland Birth Cohort revealed that 29.9% of the children surviving perinatal brain damage developed cerebral palsy, epilepsy or mental retardation (IQ less than 71)[393].	In the 1966 North Finland Birth Cohort, six of the 125 survivors (4.8%) of severe perinatal brain damage developed later schizophrenia[394]. However, these data cannot exclude perinatal brain damage due to OBC, which is associated with coronary heart disease and stroke (see row above).	
Preterm infants	Since length of gestation and preterm birth is not associated with coronary heart disease[314] or stroke[315], the effect of neurodevelop-mental disturbances independent of vascular factors can only be investigated in preterm infants. Very preterm infants (< 32 weeks of gestational age), have shown high rates of neurodevelop-mental disability in follow-up studies with 5% to 15% having cerebral palsy, severe neurosensory impairment and 25% to 50% having	Increased rate of psychiatric hospitalization for non-affective psychoses (i.e. schizophrenia and schizoaffective psychosis) from 0.05% (495 of 1022431 cases) for term birth to 0.1% (6 of 5125 cases) for very preterm infants (< 32 weeks of gestational age)[317].	0.1%

	cognitive, behavioral, and social difficulties that impede progress in school[316].		

§ defined as hallucinations and/or delusions with or without disorientation

DISCUSSION

Evidence for ischemia during schizophrenic psychosis

Table S25. Supplementary references for Figure 4

**CBF** (ml/100 g/min)	**Consequence**	**Similar findings in schizophrenia**
100	Hyperperfusion in man[395]. Focal hyperperfusion after ischemia[396]	Regional cerebral hyperperfusion and hypoperfusion in unmedicated patients[397-399]
60	Normal CBF depending on species[400-402]	
50	Mean human CBF[403]	
40	Mild hypoperfusion (oligemia)[395,403]	Reduced CBF and CBV mostly frontal (review[404])
35	Onset of decrease in oxidative phosphorylation and increased generation of reactive oxygen species (ROS) causing mitochondrial damage[405]. Mild ischemia may be much more damaging than total ischemia because the availability of sufficient oxygen for producing ROS[405].	Evidence for increased oxidative damage[406-408], mitochondrial dysfunction[409-411] (reviews[412,413], and impaired energy metabolism (review[414]) have been found in the post-mortem brains of schizophrenic patients.
30[§]	Depression of protein synthesis at approximately 60 - 40 % of mean CBF[415,416]. After 7 hrs, onset of loss of dendritic structure and spines at 64% of perfusion in some animals[417].	Only 1 rather preliminary study measured protein metabolism in 4 schizophrenic patients and found focal suppression of protein metabolism in the parietal cortex of 1 patient[418]. Reduced dendritic spine density and arborization[419-421].
25[§]	Beginning of decrease in phosphocreatine (high energy phosphate) and increase in lactate[422] (review[423]).	Decrease of phosphocreatine and ATP in the frontal cortex of neuroleptic-free schizophrenic patients[424], but not in medicated or chronic patients[425]. Increase of lactate was found in the blood[426], post-mortem brain tissue[409], and CSF[427] of schizophrenic patients.

22	Increase in cortical conduction time[428] and reduction in the amplitude of evoked potentials (EP)[403,429].	Increase in latency and reduction in the amplitude of EP (ERP, event-related potentials) is one of the most replicable biological marker in schizophrenia[430](meta-analysis[431]). The same changes have been reported for gamma oscillations[432,433].
20	Functional threshold (mild paresis), penumbra threshold[395].	Disturbed voluntary motor activity in schizophrenic patients[434]. Increased frequency of frontal release signs (primitive reflexes, i.e. soft neurological signs) in transient ischemic attacks[435] and schizophrenia[436,437].
18	Disturbance of energy metabolism: anaerobic glycolysis at ca. 33 % of mean CBF, corresponding to 17 ml/100g/min in humans[401,416].	Reduction of glycolysis was found in brain tissue of schizophrenic patients obtained during prefrontal leukotomies[438] or post-mortem[409,439,440].
18	Threshold for synaptic transmission failure (range 20 – 8)[400,416,429].	
18§	Evoked potentials abolished (range 22 – 6)[401,441].	
18	Nerve cells cease spontaneous activity[401].	
15§	Drop in ATP (range 15 - 8 depending on species[416,422].	
15	Terminal depolarization and potassium efflux[401]	
12	Critical threshold[395]	
10	Membrane failure[401]	
8	Irreversible damage, infarction[395]	Nine of 10 post-mortem studies reported focal infarctions in the brain of schizophrenic patients[442] (review[443]).

Legend for Table S25: § Values from animal studies were converted to approximate CBF values for humans by using the percentage of reductions from mean CBF. However, this approach cannot take into account the physiological and biochemical differences among species.

Table S26. Signs of cerebral ischemia in adult schizophrenic patients

**Consequences of cerebral hypoxia/ischemia**	**Presence in schizophrenic patients**
**Biochemical signs**	
Mitochondrial dysfunction follows incomplete cerebral ischemia despite reperfusion[444-446].	Reduced oxygen uptake in brain tissue from schizophrenic patients[447], impaired energy metabolism (review[414]), mitochondrial dysfunction[313,409-411] (reviewed in[412,413]).
Lactate formation is increased during hypoxia-ischemia (reviews[423,448]).	Increase of lactate in schizophrenic patients has been reported for blood[426], post-mortem brain tissue[409], and CSF[427].
Oxidative stress: Incomplete ischemia generates reactive oxygen species (ROS), which attack mitochondrial lipids, proteins, and DNA[444-446].	Evidence for increased oxidative damage[406-408]
ROS initiate lipid peroxidation[449]	Increased lipide peroxides[450-453]
Inflammation is activated in response to focal cerebral ischemia[454,455].	Inflammatory signs in schizophrenia (reviewed in[456]), increased hsCRP levels (meta-analysis[457]).
Increased Endothelin 1 (ET-1)[458]	ET-1 has a very long-lasting constrictive effect on cerebral vasculature[459]. ET-1 blood values are elevated in cerebral ischemia[458] and schizophrenia[460].
Increased S100B serum level[461]	Increased serum S100B[462,463]
High skeletal muscle creatine kinase (CK) in serum after stroke[464]	High skeletal muscle creatine kinase in serum during acute schizophrenic psychosis[465-467]
Impaired Blood-Brain Barrier (BBB)[145]	Evidence for increased BBB permeability in 5% - 20% of schizophrenic patients[468-470]
**Cellular signs**	
Hypoxia/ischemia and chronic cerebral hypoperfusion lead to slight degeneration of astrocytic end-feet processes and BBB disruption[471,472].	Signs of ultrastructural damage to capillaries of the neocortex in schizophrenic patients that resemble those observed in chronic hypoperfusion, oxidative stress, damaged blood brain-barrier, or cerebral ischemia[473].
Mitochondrial damage[474]	Reduced density of mitochondria as well as deformed, hypoplastic and small mitochondria (review[406])
Oligodendrocyte are selectively vulnerable to ischemia{Lyons	Neuropathological, transcriptomic, proteomic and brain imaging studies show

1998) (reviewed in[475]).	damage of oligodendrocytes and myelin (reviewed in[476-479]).
Loss of spine and dendrite structure[417]	Lower density of dendritic spines, reduced dendritic arborizations[480], and decreased presynaptic protein markers (reviewed in[481,482]).
**Electroencephalographic signs**	
Slowing of EEG[400,483], increased latency of evoked potentials (EPs, ERPs)[403]	Delayed latency of P300 (meta-analyses[484,485])
Reduced amplitude in ERPs[400,403]	Reduced amplitude of P300 (meta-analysis[484] decreasing with symptom exacerbations and increasing with improvements[486] (reviewed in[430]).
Gamma-oscillations are highly vulnerable to hypoxia[487,488].	Delayed latency and decreased magnitude of gamma-oscillations[432,433]
**Brain imaging evidence**	
Hypoperfusion[395]	Hypoperfusion in frontal cortex (hypofrontality), parietal cortex and medial cingulate gyri) (meta-analyses[489,490]; review[404]). Hypoperfusion correlates with negative symptoms[397,398]. Amelioration of regional cerebral blood flow is associated with clinical improvement[491].
Focal hyperperfusion after ischemia[396].	Regional cerebral hyperperfusion and hypoperfusion in unmedicated patients[397-399,404]. Positive symptoms correlated with either cerebral hyper- or hypoperfusion. This correlation disappeared after reduction of positive symptoms[398].
**Clinical signs of hypoxia**	
Inappropriate affect and facial expression, silly laugther during experimental hypoxia[492]	Inappropriate affect[493,494], elation[494], inappropriate laughter[495], and silly emotions[496].
Perseveration during experimental hypoxia[492]	Perseveration[494]
Cognitive impairment during experimental hypoxia[492]	Cognitive impairment is the core of the disorder[493] and begins between the premorbid phase and first episode[497]

The foundation of the neurodevelopmental hypothesis can also be explained by adult vascular disorders

The left column of Table S27 lists the evidence interpreted as support for the neurodevelopmental hypothesis according to reviews by Harrison[498], Moises et al.[499], Marenco et al.[377], and Weinberger[378]. The right column shows findings of a literature search in PUBMED and Google SCHOLAR using the key words of the left column and "cardiovascular" or "cerebrovascular".

Summing up, the foundation of the neurodevelopmental hypothesis is not only associated with schizophrenia, but also with adult vascular disorders (depicted in supplementary Fig. S7). In conclusion, schizophrenia as adult vascular disorder is an alternative explanation for the available evidence previously interpreted as support for the neurodevelopmental hypothesis.

Table S27. The foundation of the neurodevelopmental hypothesis and adult vascular disorder as alternative explanation.

**Evidence supporting the ND hypothesis**	**Alternative explanation**
**Prenatal evidence**	
Broadly defined **obstetric complications** (OC)[377,378,498] are associated with an increased risk for schizophrenia (meta-analysis[500]). However, overall effect of OC on the occurrence of schizophrenia is small[377]. 93% of schizophrenic patients did not experience such OC (see Table S28).	**Obstetric complications** (OC) are frequently a sign of placental inefficiency[388,501], which causes fetal undernutrition, intrauterine growth restriction (IUGR), OBC, insulin-resistance, type 2 diabetes and increased risk for cardiovascular disorders[387,388], and stroke in adulthood[314,315,389,390] (reviewed in[391,392]).
**Preeclampsia**[377]: The only OC study able to adjust for mother's psychotic illness during her adult life found only preeclampsia to be significantly associated with an increased risk for schizophrenia[502].	**Preeclampsia** has a strong genetic component (reviewed in[503]) associated with an increased risk of cardiovascular or cerebrovascular disease (meta-analysis[504]).
**Birth weight**[377] is inversely related to schizophrenia[505].	**Birth weight** is inversely related to systolic blood pressure, ischemic heart disease, and stroke[506].
**Maternal influenza**[377,378,498]	**Maternal influenza** is associated with an 20% increase in cardiovascular disease[507].
**Prenatal famine**[377,378,498] results in a 2-fold increase of risk for schizophrenia[508-510].	**Prenatal famine** causes increase of hypertension, raised glucose levels, increased blood pressure response to stress,

	and a 2-fold increase of risk for coronary heart disease (reviewed in[510]).
**Blood group incompatibilities**[378,511]	Thickening of the amniotic epithelium[512] and the trophoblast basement membrane[513] suggests reduced diffusion and availability of nutrients mimicking prenatal famine.
**Winter birth**[498]. The effect correlates with the latitude[514] (colder winter temperatures) and is not detectable in the Southern Hemisphere[515] (relatively warm winters).	Cold outdoor temperature at birth is associated with increased coronary heart disease and insulin resistance[516].
**Maternal homocysteine level** elevated[378]	Elevated homocysteine concentrations at pregnancy are associated with increased risk of cardiovascular disease, angina, and stroke (reviewed in[517]).
**Paternal age** at conception[378]	Paternal age results in reduced telomere length in his offspring[518]. Reduced telomere length is associated with premature myocardial infarction[519].
**Maternal severe stress** during pregnancy[378]	Maternal stress, anxiety, and glucocorticoids reduce fetal growth and birth weight, and predispose the offspring to adult cardiovascular disorder and stroke[520,521].
**Structural cerebral abnormalities** (ventricular enlargement, reduced cortical volume)[498]	Progressive changes in ventricular and gray matter volume challenge the neurodevelopmental hypothesis (meta-analyses[522,523])
**Neuropathology**[498]: Reported cytoarchitectural abnormalities related to intrauterine development have not been replicated and are not unequivocally established[443].	In comparison, the undisputed cytoarchitectural findings, such as alterations in neuronal size, and synaptic and dendritic organization, could well originate much later[443].
**Postnatal evidence**	
**Delays** in motor and speech development[378,498], poor motor coordination[377]. The delays are very modest and do not cause concern to physicians or parents. Furthermore, most individuals with such a delay do not develop	Maturational delay, i.e., slow growth in fetal life, infancy, and during childhood is associated with adult cerebrovascular disorder[524]. Furthermore, delays in motor development are associated with the personality dimension of neuroticism (trait anxiety) in adulthood[525]. Neuroticism seems to predispose to schizophrenia[526].

schizophrenia[378].	
**Behavioural abnormalities** as infants[498] such as hyperactivity, poor verbal abilities, nervous, withdrawn, or disruptive, aggressive, and antisocial behavior in school[377].	These behavioural abnormalities are characteristic for the two personality dimensions frequently observed in schizophrenic patients: introversion / neuroticism (trait anxiety)[527] and impulsive/antisocial/psychopathic personality[528-530]. Social introversion and psychoticism/psychopathy (type A behaviour pattern) are associated with an increased risk for myocardial infarction[531,532]
**Soft neurological signs** have been observed in up to 60% of schizophrenic patients and are interpreted as evidence for premorbid brain damage in schizophrenia[436,437] (meta-analysis[533]).	These signs are present in up to 40.6% of normal individuals (meta-analysis[533]). Transient ischemia increases the frequency of these signs[435]. In schizophrenic patients, soft neurological signs decrease in parallel with the remission of acute psychosis (meta-analysis[534]).
**Enuresis** in childhood[378,535]	Enuresis is associated with anxiety/ withdrawal[536], extraversion[537], conduct problems, attention deficit behaviors, and anxious/withdrawn[536] or antisocial/ psychopathic personality[538]. The latter predisposes to schizophrenia[528-530]
**Lower premorbid IQ** of about 0.5 standard deviations (SD)[378] (meta-analysis[539]).	The superior premorbid high intelligence (IQ) of a considerable number of patients{ [540-542] is not compatible with a premorbid fronto-cortical brain damage. However, a premorbid high IQ is not at variance with the adult ischemia hypothesis. .

Figure S7. Foundation of neurodevelopmental hypothesis and adult vascular disorder as alternative explanation.

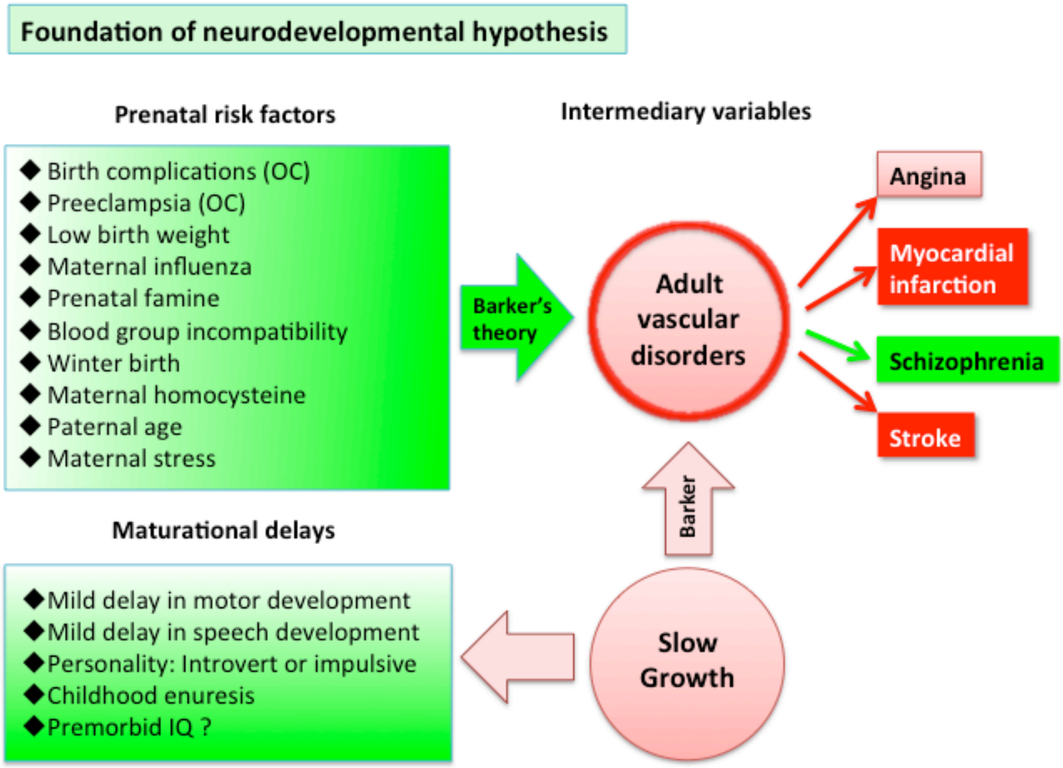

Figure S7. The foundation of the neurodevelopmental hypothesis of schizophrenia consists of epidemiological studies showing that prenatal factors and delayed growth are associated with an increased risk for schizophrenia (in green, for reviews[377,378,498,499]). The same factors increase the risk for adult cardiovascular and cerebrovascular disorders (in red, references in supplementary Table S27), also known as Barker's theory[391,392,524]. Adult vascular disorder as intermediary variable between broadly defined birth complications (OC) and schizophrenia has been ignored suggesting that OC are a proxy variable for the predisposition to adult cerebrovascular disorders, and that the Barker theory might be a well-founded substitute for the neurodevelopmental hypothesis to explain prenatal risk factors and maturational delays in schizophrenia. IQ, intelligence quotient; OC, obstetric complications.

Normal or superior intelligence in schizophrenia

The average premorbid IQ of schizophrenic patients is on average only 0.5 SD below the population average[539], which might be explained by their higher trait anxiety (neuroticism)[526] and not necessarily an indication of a neurodevelopmental brain damage. Trait anxiety is known to correlate positively with test anxiety and negatively with IQ test results (reviewed in[543])

The Danish draft-board study by Ufer-Parnas et al. found a premorbid uni-modal normal distribution (mean 94.38, SD 16.24) in schizophrenia[541]. Superior intelligence or the cognitive abilities of genius are often defined as an IQ ≥ 120 or IQ ≥ 130, respectively. The percentage and number of schizophrenic patients exceeding that level can be calculated from the normal distribution of IQ scores, 0.4% for global lifetime prevalence[544], and an estimated global population of 7 billion for 2015.

The results show that 36.5%, 10.2%, 5.7%, and 1.4% of schizophrenic patients are expected to premorbidly have an IQs of ≥ 100, ≥ 115, ≥ 120, ≥ 130 corresponding globally to 10.2 millions of schizophrenic patients with a normal IQ ≥ 100, 2.8 millions with IQ ≥ 115, 1.6 millions with IQ ≥ 120, and 0.4 millions with IQ ≥ 130. Such a large number of individuals with normal or superior intelligence contradicts the postulated neurodevelopmentally caused premorbid brain defect.

Birth complications in only 7% of schizophrenic patients

Data for calculating the percentage of obstetric complications in schizophrenic patients were obtained from Table 2 of the meta-analysis of prospective population-based studies by Cannon et al. (2002)[500].

In 93% of schizophrenic patients and in 94.6% of normal controls, no evidence of (broadly defined) birth complications were found (see Table S28). In conclusion, the overall majority of schizophrenic patients were not exposed to birth complications that might have caused a defect of brain development.

Table S28. Percentage of schizophrenic patients with broadly defined birth complications

Obstetric complications	Schizophrenic patients	Exposed	Controls	Exposed
Diabetes in pregnancy	237	3	1909	3
Placental abruption	308	3	508352	1643
Birth weight <2000 g	504	6	10926	78
Emergency Cesarean section	818	20	507863	1595
Congenital malformations	737	10	508781	6144
Uterine atony	659	27	507703	16913
Rhesus variables	759	18	17537	2911
Threatened premature delivery	308	8	508352	6498
Asphyxia	1109	60	2297	119
Bleeding in pregnancy	1223	34	524972	9367
Birth weight <2500 g	1294	60	536045	19343
Head circumference <32 cm	758	53	508315	15388
Smoking in pregnancy	105	26	17886	5752
Preeclampsia	1712	75	510275	18286
Anemia in pregnancy	522	20	1526	96
Gestational age <37 weeks	1290	67	536051	21710
Small for gestational age	1272	86	519229	23485
Induction of labor	689	186	2361	232
Apgar score <7 at 1 minute after birth	390	18	507434	22771
Gestational age >42 weeks	1187	34	508747	16065
Child stayed in hospital after mother discharged	973	110	1488	99
Forceps delivery or vacuum extraction	1724	124	527058	29753
Birth length <49 cm	761	130	51320	105205
Cephalopelvic disproportion	662	10	2338	42
Cord around neck	893	171	1345	333
Cesarean section	1214	63	526045	42947
Birth weight <2500 g and premature	954	41	11376	215
Nonvertex presentation	1667	74	510208	61130
Breech delivery	464	11	508508	13580
Urinary tract infection in pregnancy	690	20	507730	7115
Nonspontaneous delivery	331	46	17108	1554
Total	23208	1614	7300987	391603

**Percentage with complications**	**7,0%**	**5,4%**
**Percentage without complications**	**93,0%**	**94,6%**
**Difference**	**1.6%**	

Data from[500].

Schizophrenia treatments improve CBF and ischemia protection

All treatments found to improve schizophrenia also improve cerebral perfusion and/or protect against ischemia or its harmful consequences such as inflammation (see Table S29).

Table S29. Effects of treatments for schizophrenia on cerebral perfusion and ischemia.

Treatment in schizophrenia	Effects
Acetylsalicylic acid[545]	prevents cerebral ischemia[546]
Atypical antipsychotics[547,548]	enhance CBF[397,491,549]. Clinical improvement correlates with CBF[491]. A recent meta-analysis provides a more variable picture with increased as well as decreased areas of rCBF following antipsychotic treatment[550].
Celecoxib[551-553], nonsteroidal anti-inflammatory drugs (NSAID)[554]	Celecoxib is an non-steroidal anti-inflammatory drug with potent neuroprotective effect against ischemia-induced inflammatory reaction[555]
Electroconvulsive therapy (ECT)[556]	Epileptic seizures are accompanied by an increase in focal CBF[557,558]. ECT improves CBF and catatonia[559]
Erythropoietin (EPO)[560]	EPO enhances cerebral vasodilatation[561], activates the PI3K/Akt pathway[562] and improves the consequences of cerebral ischemia[563]
Exercise[564]	increases cerebral vasodilatation, BDNF[565], and cerebral blood volume in the hippocampus[566]
Ginkgo Biloba Extract[567,568]	increases CBF[569,570], and protects against cerebral ischemia[571]
Glucose[572,573]	Glucose, the obligatory energy substrate for the brain[574], is lacking in ischemia.
Insulin Coma Therapy (ICT)[575,576]	Insulin causes cerebral vasodilatation[577], and activates the PI3K/Akt pathway in neurons following brain ischemia[578]. Furthermore, insulin-induced hypoglycemia leads to a marked increase in CBF[579].
Nicotine (alpha-7 nicotinic agonists)[580,581]	enhances cholinergic vasodilation in the cerebral cortex[582]
Reserpine[583]	depletes dopamine and noradrenaline from the brain[584]. Its use as antihypertensive drug suggests a vascular effect[585].
Transcranial Magnetic Stimulation (rTMS)[586,587]	increases CBF in some areas of the brain[588-590].
Typical neuroleptics[591]	Dopamine causes a dose-dependent vasoconstriction in about 50% of cortical microvessels[69].

	Antipsychotic drugs block D2/3 receptors[592] and increase CBF[397,491,549,593], mainly in paranoid patients[550]. But see also evidence for a decrease of CBF[490,550]. Clinical improvement was found in one study to correlate with CBF[491].

Table S30. Evidence for repair mechanisms in schizophrenia.

	**Evidence**
1	Adult neurogenesis and synaptic plasticity are involved in postischemic repair and in schizophrenia (reviewed in[594,595]).
2	Adult neurogenesis and synaptic plasticity have been implicated in schizophrenia by previous pathway analyses (see supplementary information).
3	Motor endplate alterations in schizophrenic patients resemble axonal destruction followed by regeneration[596].
4	Erythropoietin, a stimulator of adult neurogenesis, improves cognitive functions in chronic schizophrenic patients[560].
5	Neuroleptics appear to stimulate adult neurogenesis either directly or indirectly via prolactin[597,598].
6	Drug responding patients show signs of myelin repair in brain imaging[599].
7	Physical exercise increases BDNF, adult neurogenesis, hippocampal volume, and improves negative symptoms in schizophrenic patients[564,565,600,601].
8	The PI3K/Akt pathway mediates not only the effects of stress, growth factors, and hormones on metabolism, vasoconstriction, and vasodilatation, but also on synaptic plasticity and adult neurogenesis, i.e., repair[602,603] (see Fig. 2).
9	Neural stem cell proliferation required for adult neurogenesis is reduced in schizophrenia, but not in major depression[604].

The AVIH seems to offer a better explanation for the evidence compared to the NDH

Theory choice is a search for the best explanation of the evidence. The three main criteria for the evaluation of a hypothesis are consilience, simplicity, and analogy[605]. The adult vascular-ischemia hypothesis better fulfills the criteria of consilience, simplicity, and analogy than the neurodevelopmental hypothesis. For consilience, see Table S31.

Table S31. Consilience of the NDH and the AVIH with the evidence

Evidence/facts		Explanation by	
		**ND hypothesis**	**AVI hypothesis**
	**Genetic**		
1	Overrepresentation of VI[606], ND, and repair genes (Table S22, Fig. 3a)	**Yes**	**Yes**
	**Prenatal**		
2	Evidence supporting the ND hypothesis (Table S27 and Fig. S7)	**Yes**	**Yes**
3	Absence of birth complications in 93% of schizophrenic patients, difference to normal population only 1.6% (meta-analysis[500]) (see Table S28)	No	**Yes**
4	Absence of minor physical signs in 65% of patients[607] (reviewed in[608])	No	**Yes**
5	Absence of neuropathological evidence for neurodevelopmental brain defect[443,482]	No	**Yes**
	**Premorbid**		
6	High premorbid intelligence (IQ)[540-542,609-612] (supplementary info above)	No	**Yes**
	**Schizophrenic psychosis**		
7	Late-onset schizophrenia (reviewed in[613])	No	**Yes**
8	Signs of cerebral ischemia during acute psychosis (Fig. 4, Table S26)	No	**Yes**
9	Progressive brain tissue loss[614] (meta-analysis[523])	No	**Yes**
10	Improvement by blockade of dopamine D2/D3 and 5-HT2A receptor (review[178]) (see Fig. 1)	No	**Yes**
11	Soft neurological signs decrease in parallel with the remission of acute psychosis (meta-analysis[534]) (see also refs. in Table 27).	No	**Yes**
12	Course: remissions, relapses, and progressions[615]	No	**Yes**
13	Treatment-dependent outcome (review[608])	No	**Yes**

Legend for Table S31. AVI, adult vascular-ischemia; ND, neurodevelopmental; VI, vascular-ischemia.

The criterion of simplicity is met by requiring less auxiliary hypotheses, e.g., for evidence # 3–13 in Table S31 above. Finally, disorders disturbing the cerebral energy-supply (Table 23, Fig. S6) and adult vascular disorders (Fig. 7) provide useful analogies for better understanding the pathogenesis of schizophrenia, whereas the analogy of the NDH with neurodevelopmental disturbances in very preterm infants is surprisingly unconvincing (see supplementary Fig. S7, Tables S23–S24).

Supplementary References

1 Allen NC, Bagade S, McQueen MB, Ioannidis JP, Kavvoura FK, Khoury MJ, *et al.* Systematic meta-analyses and field synopsis of genetic association studies in schizophrenia: the SzGene database. *Nat Genet* 2008;**40:**827-834.

2 Kidd KK. Associations of disease with genetic markers: déjà vu all over again. *Am J Med Genet* 1993;**48:**71-73.

3 Welter D, MacArthur J, Morales J, Burdett T, Hall P, Junkins H, *et al.* The NHGRI GWAS Catalog, a curated resource of SNP-trait associations. *Nucleic Acids Res* 2014;**42:**D1001-D1006.

4 Ayalew M, Le-Niculescu H, Levey DF, Jain N, Changala B, Patel SD, *et al.* Convergent functional genomics of schizophrenia: from comprehensive understanding to genetic risk prediction. *Mol Psychiatry* 2012;**17:**887-905.

5 Schizophrenia Working Group of the Psychiatric Genomics Consortium. Biological insights from 108 schizophrenia-associated genetic loci. *Nature* 2014;**511:**421-427.

6 Torkamani A, Topol EJ, Schork NJ. Pathway analysis of seven common diseases assessed by genome-wide association. *Genomics* 2008;**92:**265-272.

7 Honkaniemi J, Sharp FR. Global ischemia induces immediate-early genes encoding zinc finger transcription factors. *J Cereb Blood Flow Metab* 1996;**16:**557-565.

8 Covini N, Tamburin M, Consalez G, Salvati P, Benatti L. ZFM1/SF1 mRNA in rat and gerbil brain after global ischaemia. *Eur J Neurosci* 1999;**11:**781-787.

9 Ide T, Takada K, Qiu JH, Saito N, Kawahara N, Asai A, *et al.* Ubiquitin stress response in postischemic hippocampal neurons under nontolerant and tolerant conditions. *J Cereb Blood Flow Metab* 1999;**19:**750-756.

10 Soriano MA, Tessier M, Certa U, Gill R. Parallel gene expression monitoring using oligonucleotide probe arrays of multiple transcripts with an animal model of focal ischemia. *J Cereb Blood Flow Metab* 2000;**20:**1045-1055.

11 Sun HB, Yokota H, Chi XX, Xu ZC. Differential expression of neurexin mRNA in CA1 and CA3 hippocampal neurons in response to ischemic insult. *Brain Res Mol Brain Res* 2000;**84:**146-149.

12 Bates S, Read SJ, Harrison DC, Topp S, Morrow R, Gale D, *et al.* Characterisation of gene expression changes following permanent MCAO in the rat using subtractive hybridisation. *Brain Res Mol Brain Res* 2001;**93:**70-80.

13 Jin K, Mao XO, Eshoo MW, Nagayama T, Minami M, Simon RP, *et al.* Microarray analysis of hippocampal gene expression in global cerebral ischemia. *Ann Neurol* 2001;**50:**93-103.

14 Gozal E, Gozal D, Pierce WM, Thongboonkerd V, Scherzer JA, Sachleben LR, *et al.* Proteomic analysis of CA1 and CA3 regions of rat hippocampus and differential susceptibility to intermittent hypoxia. *J Neurochem* 2002;**83:**331-345.

15 Jin K, Mao XO, Eshoo MW, del Rio G, Rao R, Chen D, *et al.* cDNA microarray analysis of changes in gene expression induced by neuronal hypoxia in vitro. *Neurochem Res* 2002;**27:**1105-1112.

16 Keyvani K, Witte OW, Paulus W. Gene expression profiling in perilesional and contralateral areas after ischemia in rat brain. *J Cereb Blood Flow Metab* 2002;**22:**153-160.

17 Kim SH, Won SJ, Sohn S, Kwon HJ, Lee JY, Park JH, *et al.* Brain-derived neurotrophic factor can act as a pronecrotic factor through transcriptional and translational activation of NADPH oxidase. *J Cell Biol* 2002;**159:**821-831.

18 Raghavendra Rao VL, Bowen KK, Dhodda VK, Song G, Franklin JL, Gavva NR, *et al.* Gene expression analysis of spontaneously hypertensive rat cerebral cortex following transient focal cerebral ischemia. *J Neurochem* 2002;**83:**1072-1086.

19 Schmidt-Kastner R, Zhang B, Belayev L, Khoutorova L, Amin R, Busto R, *et al.* DNA microarray analysis of cortical gene expression during early recirculation after focal brain ischemia in rat. *Brain Res Mol Brain Res* 2002;**108:**81-93.
20 Tang Y, Lu A, Aronow BJ, Wagner KR, Sharp FR. Genomic responses of the brain to ischemic stroke, intracerebral haemorrhage, kainate seizures, hypoglycemia, and hypoxia. *Eur J Neurosci* 2002;**15:**1937-1952.
21 Trendelenburg G, Prass K, Priller J, Kapinya K, Polley A, Muselmann C, *et al.* Serial analysis of gene expression identifies metallothionein-II as major neuroprotective gene in mouse focal cerebral ischemia. *J Neurosci* 2002;**22:**5879-5888.
22 Gilbert RW, Costain WJ, Blanchard ME, Mullen KL, Currie RW, Robertson HA. DNA microarray analysis of hippocampal gene expression measured twelve hours after hypoxia-ischemia in the mouse. *J Cereb Blood Flow Metab* 2003;**23:**1195-1211.
23 Hayashi T, Noshita N, Sugawara T, Chan PH. Temporal profile of angiogenesis and expression of related genes in the brain after ischemia. *J Cereb Blood Flow Metab* 2003;**23:**166-180.
24 Lu A, Tang Y, Ran R, Clark JF, Aronow BJ, Sharp FR. Genomics of the periinfarction cortex after focal cerebral ischemia. *J Cereb Blood Flow Metab* 2003;**23:**786-810.
25 Stenzel-Poore MP, Stevens SL, Xiong Z, Lessov NS, Harrington CA, Mori M, *et al.* Effect of ischaemic preconditioning on genomic response to cerebral ischaemia: similarity to neuroprotective strategies in hibernation and hypoxia-tolerant states. *Lancet* 2003;**362:**1028-1037.
26 Dhodda VK, Sailor KA, Bowen KK, Vemuganti R. Putative endogenous mediators of preconditioning-induced ischemic tolerance in rat brain identified by genomic and proteomic analysis. *J Neurochem* 2004;**89:**73-89.
27 Hammond EM, Giaccia AJ. The role of ATM and ATR in the cellular response to hypoxia and re-oxygenation. *DNA Repair (Amst)* 2004;**3:**1117-1122.
28 Jin K, Mao XO, Greenberg DA. Proteomic analysis of neuronal hypoxia in vitro. *Neurochem Res* 2004;**29:**1123-1128.
29 Katsuki H, Nonaka M, Shirakawa H, Kume T, Akaike A. Endogenous D-serine is involved in induction of neuronal death by N-methyl-D-aspartate and simulated ischemia in rat cerebrocortical slices. *J Pharmacol Exp Ther* 2004;**311:**836-844.
30 Kawahara N, Wang Y, Mukasa A, Furuya K, Shimizu T, Hamakubo T, *et al.* Genome-wide gene expression analysis for induced ischemic tolerance and delayed neuronal death following transient global ischemia in rats. *J Cereb Blood Flow Metab* 2004;**24:**212-223.
31 Kim JB, Piao CS, Lee KW, Han PL, Ahn JI, Lee YS, *et al.* Delayed genomic responses to transient middle cerebral artery occlusion in the rat. *J Neurochem* 2004;**89:**1271-1282.
32 Lu XC, Williams AJ, Yao C, Berti R, Hartings JA, Whipple R, *et al.* Microarray analysis of acute and delayed gene expression profile in rats after focal ischemic brain injury and reperfusion. *J Neurosci Res* 2004;**77:**843-857.
33 MacManus JP, Graber T, Luebbert C, Preston E, Rasquinha I, Smith B, *et al.* Translation-state analysis of gene expression in mouse brain after focal ischemia. *J Cereb Blood Flow Metab* 2004;**24:**657-667.
34 Nagata T, Takahashi Y, Sugahara M, Murata A, Nishida Y, Ishikawa K, *et al.* Profiling of genes associated with transcriptional responses in mouse hippocampus after transient forebrain ischemia using high-density oligonucleotide DNA array. *Brain Res Mol Brain Res* 2004;**121:**1-11.
35 Oprica M, Van Dam AM, Lundkvist J, Iverfeldt K, Winblad B, Bartfai T, *et al.* Effects of chronic overexpression of interleukin-1 receptor antagonist in a model of permanent focal cerebral ischemia in mouse. *Acta Neuropathol* 2004;**108:**69-80.
36 Haqqani AS, Nesic M, Preston E, Baumann E, Kelly J, Stanimirovic D. Characterization of vascular protein expression patterns in cerebral ischemia/reperfusion using laser capture microdissection and ICAT-nanoLC-MS/MS. *FASEB J* 2005;**19:**1809-1821.

37 Haqqani AS, Kelly J, Baumann E, Haseloff RF, Blasig IE, Stanimirovic DB. Protein markers of ischemic insult in brain endothelial cells identified using 2D gel electrophoresis and ICAT-based quantitative proteomics. *J Proteome Res* 2007;**6:**226-239.
38 DeGracia DJ, Hu BR. Irreversible translation arrest in the reperfused brain. *J Cereb Blood Flow Metab* 2007;**27:**875-893.
39 Föcking M, Besselmann M, Trapp T. Proteomics of experimental stroke in mice. *Acta Neurobiol Exp (Wars)* 2006;**66:**273-278.
40 Ford G, Xu Z, Gates A, Jiang J, Ford BD. Expression Analysis Systematic Explorer (EASE) analysis reveals differential gene expression in permanent and transient focal stroke rat models. *Brain Res* 2006;**1071:**226-236.
41 Gidday JM. Cerebral preconditioning and ischaemic tolerance. *Nat Rev Neurosci* 2006;**7:**437-448.
42 Haseloff RF, Krause E, Bigl M, Mikoteit K, Stanimirovic D, Blasig IE. Differential protein expression in brain capillary endothelial cells induced by hypoxia and posthypoxic reoxygenation. *Proteomics* 2006;**6:**1803-1809.
43 Tang Y, Pacary E, Fréret T, Divoux D, Petit E, Schumann-Bard P, *et al.* Effect of hypoxic preconditioning on brain genomic response before and following ischemia in the adult mouse: identification of potential neuroprotective candidates for stroke. *Neurobiol Dis* 2006;**21:**18-28.
44 Won SJ, Kim SH, Xie L, Wang Y, Mao XO, Jin K, *et al.* Reelin-deficient mice show impaired neurogenesis and increased stroke size. *Exp Neurol* 2006;**198:**250-259.
45 Cid C, Garcia-Bonilla L, Camafeita E, Burda J, Salinas M, Alcazar A. Proteomic characterization of protein phosphatase 1 complexes in ischemia-reperfusion and ischemic tolerance. *Proteomics* 2007;**7:**3207-3218.
46 Lee BJ, Lee JG, Kim YH. A 12-week, double-blind, placebo-controlled trial of donepezil as an adjunct to haloperidol for treating cognitive impairments in patients with chronic schizophrenia. *J Psychopharmacol* 2007;**21:**421-427.
47 Mitsios N, Saka M, Krupinski J, Pennucci R, Sanfeliu C, Wang Q, *et al.* A microarray study of gene and protein regulation in human and rat brain following middle cerebral artery occlusion. *BMC Neurosci* 2007;**8:**93.
48 Tsuchiya A, Hayashi T, Deguchi K, Sehara Y, Yamashita T, Zhang H, *et al.* Expression of netrin-1 and its receptors DCC and neogenin in rat brain after ischemia. *Brain Res* 2007;**1159:**1-7.
49 Yang Y, Estrada EY, Thompson JF, Liu W, Rosenberg GA. Matrix metalloproteinase-mediated disruption of tight junction proteins in cerebral vessels is reversed by synthetic matrix metalloproteinase inhibitor in focal ischemia in rat. *J Cereb Blood Flow Metab* 2007;**27:**697-709.
50 Arboleda-Velasquez JF, Zhou Z, Shin HK, Louvi A, Kim HH, Savitz SI, *et al.* Linking Notch signaling to ischemic stroke. *Proc Natl Acad Sci U S A* 2008;**105:**4856-4861.
51 Dave KR, DeFazio RA, Raval AP, Torraco A, Saul I, Barrientos A, *et al.* Ischemic preconditioning targets the respiration of synaptic mitochondria via protein kinase C epsilon. *J Neurosci* 2008;**28:**4172-4182.
52 Van Elzen R, Moens L, Dewilde S. Expression profiling of the cerebral ischemic and hypoxic response. *Expert Rev Proteomics* 2008;**5:**263-282.
53 de Bilbao F, Arsenijevic D, Moll T, Garcia-Gabay I, Vallet P, Langhans W, *et al.* In vivo over-expression of interleukin-10 increases resistance to focal brain ischemia in mice. *J Neurochem* 2009;**110:**12-22.
54 Dirnagl U, Becker K, Meisel A. Preconditioning and tolerance against cerebral ischaemia: from experimental strategies to clinical use. *Lancet Neurol* 2009;**8:**398-412.
55 Jin G, Tsuji K, Xing C, Yang YG, Wang X, Lo EH. CD47 gene knockout protects against transient focal cerebral ischemia in mice. *Exp Neurol* 2009;**217:**165-170.

56 Lebesgue D, Chevaleyre V, Zukin RS, Etgen AM. Estradiol rescues neurons from global ischemia-induced cell death: multiple cellular pathways of neuroprotection. *Steroids* 2009;**74:**555-561.
57 Sevimli S, Diederich K, Strecker JK, Schilling M, Klocke R, Nikol S, *et al.* Endogenous brain protection by granulocyte-colony stimulating factor after ischemic stroke. *Exp Neurol* 2009;**217:**328-335.
58 Wang L, Zhang X, Liu L, Cui L, Yang R, Li M, *et al.* Tanshinone II A down-regulates HMGB1, RAGE, TLR4, NF-kappaB expression, ameliorates BBB permeability and endothelial cell function, and protects rat brains against focal ischemia. *Brain Res* 2010;**1321:**143-151.
59 Buller B, Liu X, Wang X, Zhang RL, Zhang L, Hozeska-Solgot A, *et al.* MicroRNA-21 protects neurons from ischemic death. *FEBS J* 2010;**277:**4299-4307.
60 Ghosh G, Subramanian IV, Adhikari N, Zhang X, Joshi HP, Basi D, *et al.* Hypoxia-induced microRNA-424 expression in human endothelial cells regulates HIF-α isoforms and promotes angiogenesis. *J Clin Invest* 2010;**120:**4141-4154.
61 Van Elzen R, Ghesquière B, Timmerman E, Vandamme S, Moens L, Gevaert K, *et al.* Integrated proteomic analysis reveals a substantial enrichment of protein trafficking processes in hippocampus tissue after hypoxic stress. *J Proteome Res* 2010;**9:**204-215.
62 Jiao H, Wang Z, Liu Y, Wang P, Xue Y. Specific role of tight junction proteins claudin-5, occludin, and ZO-1 of the blood-brain barrier in a focal cerebral ischemic insult. *J Mol Neurosci* 2011;**44:**130-139.
63 Jin R, Song Z, Yu S, Piazza A, Nanda A, Penninger JM, *et al.* Phosphatidylinositol-3-kinase gamma plays a central role in blood-brain barrier dysfunction in acute experimental stroke. *Stroke* 2011;**42:**2033-2044.
64 Sasaki T, Takemori H, Yagita Y, Terasaki Y, Uebi T, Horike N, *et al.* SIK2 is a key regulator for neuronal survival after ischemia via TORC1-CREB. *Neuron* 2011;**69:**106-119.
65 Ziu M, Fletcher L, Rana S, Jimenez DF, Digicaylioglu M. Temporal differences in microRNA expression patterns in astrocytes and neurons after ischemic injury. *PLoS One* 2011;**6:**e14724.
66 Cauli B, Tong XK, Rancillac A, Serluca N, Lambolez B, Rossier J, *et al.* Cortical GABA interneurons in neurovascular coupling: relays for subcortical vasoactive pathways. *J Neurosci* 2004;**24:**8940-8949.
67 Fleming I. Cytochrome p450 and vascular homeostasis. *Circ Res* 2001;**89:**753-762.
68 Hamel E. Perivascular nerves and the regulation of cerebrovascular tone. *J Appl Physiol* 2006;**100:**1059-1064.
69 Krimer LS, Muly EC, Williams GV, Goldman-Rakic PS. Dopaminergic regulation of cerebral cortical microcirculation. *Nat Neurosci* 1998;**1:**286-289.
70 Lecrux C, Hamel E. The neurovascular unit in brain function and disease. *Acta Physiol (Oxf)* 2011;**203:**47-59.
71 MacIntyre J, Dong A, Straiker A, Zhu J, Howlett SE, Bagher A, *et al.* Cannabinoid and lipid-mediated vasorelaxation in retinal microvasculature. *Eur J Pharmacol* 2014;**735:**105-114.
72 Núñez E, Benito C, Pazos MR, Barbachano A, Fajardo O, González S, *et al.* Cannabinoid CB2 receptors are expressed by perivascular microglial cells in the human brain: an immunohistochemical study. *Synapse* 2004;**53:**208-213.
73 Ralevic V, Kendall DA. Cannabinoid modulation of perivascular sympathetic and sensory neurotransmission. *Curr Vasc Pharmacol* 2009;**7:**15-25.
74 Yamagata K, Andreasson KI, Kaufmann WE, Barnes CA, Worley PF. Expression of a mitogen-inducible cyclooxygenase in brain neurons: regulation by synaptic activity and glucocorticoids. *Neuron* 1993;**11:**371-386.
75 Shusta EV, Boado RJ, Mathern GW, Pardridge WM. Vascular genomics of the human brain. *J Cereb Blood Flow Metab* 2002;**22:**245-252.

76 Daneman R, Zhou L, Agalliu D, Cahoy JD, Kaushal A, Barres BA. The mouse blood-brain barrier transcriptome: a new resource for understanding the development and function of brain endothelial cells. *PLoS One* 2010;**5:**e13741.
77 Garcia-Cardeña G, Comander J, Anderson KR, Blackman BR, Gimbrone MA. Biomechanical activation of vascular endothelium as a determinant of its functional phenotype. *Proc Natl Acad Sci U S A* 2001;**98:**4478-4485.
78 Ishibazawa A, Nagaoka T, Yokota H, Ono S, Yoshida A. Low shear stress up-regulation of proinflammatory gene expression in human retinal microvascular endothelial cells. *Exp Eye Res* 2013;**116:**308-311.
79 Kinderlerer AR, Ali F, Johns M, Lidington EA, Leung V, Boyle JJ, *et al.* KLF2-dependent, shear stress-induced expression of CD59: a novel cytoprotective mechanism against complement-mediated injury in the vasculature. *J Biol Chem* 2008;**283:**14636-14644.
80 LaMack JA, Himburg HA, Zhang J, Friedman MH. Endothelial gene expression in regions of defined shear exposure in the porcine iliac arteries. *Ann Biomed Eng* 2010;**38:**2252-2262.
81 McCormick SM, Eskin SG, McIntire LV, Teng CL, Lu CM, Russell CG, *et al.* DNA microarray reveals changes in gene expression of shear stressed human umbilical vein endothelial cells. *Proc Natl Acad Sci U S A* 2001;**98:**8955-8960.
82 Mun GI, Lee SJ, An SM, Kim IK, Boo YC. Differential gene expression in young and senescent endothelial cells under static and laminar shear stress conditions. *Free Radic Biol Med* 2009;**47:**291-299.
83 Nagel T, Resnick N, Atkinson WJ, Dewey CF, Gimbrone MA. Shear stress selectively upregulates intercellular adhesion molecule-1 expression in cultured human vascular endothelial cells. *J Clin Invest* 1994;**94:**885-891.
84 Ni CW, Qiu H, Rezvan A, Kwon K, Nam D, Son DJ, *et al.* Discovery of novel mechanosensitive genes in vivo using mouse carotid artery endothelium exposed to disturbed flow. *Blood* 2010;**116:**e66-e73.
85 Noria S, Xu F, McCue S, Jones M, Gotlieb AI, Langille BL. Assembly and reorientation of stress fibers drives morphological changes to endothelial cells exposed to shear stress. *Am J Pathol* 2004;**164:**1211-1223.
86 Parmar KM, Larman HB, Dai G, Zhang Y, Wang ET, Moorthy SN, *et al.* Integration of flow-dependent endothelial phenotypes by Kruppel-like factor 2. *J Clin Invest* 2006;**116:**49-58.
87 Passerini AG, Polacek DC, Shi C, Francesco NM, Manduchi E, Grant GR, *et al.* Coexisting proinflammatory and antioxidative endothelial transcription profiles in a disturbed flow region of the adult porcine aorta. *Proc Natl Acad Sci U S A* 2004;**101:**2482-2487.
88 Roberts OL, Holmes K, Müller J, Cross DA, Cross MJ. ERK5 and the regulation of endothelial cell function. *Biochem Soc Trans* 2009;**37:**1254-1259.
89 Topper JN, Cai J, Falb D, Gimbrone MA. Identification of vascular endothelial genes differentially responsive to fluid mechanical stimuli: cyclooxygenase-2, manganese superoxide dismutase, and endothelial cell nitric oxide synthase are selectively up-regulated by steady laminar shear stress. *Proc Natl Acad Sci U S A* 1996;**93:**10417-10422.
90 White SJ, Hayes EM, Lehoux S, Jeremy JY, Horrevoets AJ, Newby AC. Characterization of the differential response of endothelial cells exposed to normal and elevated laminar shear stress. *J Cell Physiol* 2011;**226:**2841-2848.
91 Yee A, Bosworth KA, Conway DE, Eskin SG, McIntire LV. Gene expression of endothelial cells under pulsatile non-reversing vs. steady shear stress; comparison of nitric oxide production. *Ann Biomed Eng* 2008;**36:**571-579.
92 Zhang C, Zhang Z, Castle J, Sun S, Johnson J, Krainer AR, *et al.* Defining the regulatory network of the tissue-specific splicing factors Fox-1 and Fox-2. *Genes Dev* 2008;**22:**2550-2563.

93 Zhao F, Kuroiwa T, Miyasaka N, Nagaoka T, Nakane M, Tamura A, *et al.* Ultrastructural and MRI study of the substantia nigra evolving exofocal post-ischemic neuronal death in the rat. *Neuropathology* 2002;**22:**91-105.
94 Agarwal N, Lippmann ES, Shusta EV. Identification and expression profiling of blood-brain barrier membrane proteins. *J Neurochem* 2010;**112:**625-635.
95 Beauchamp NJ, van Achterberg TA, Engelse MA, Pannekoek H, de Vries CJ. Gene expression profiling of resting and activated vascular smooth muscle cells by serial analysis of gene expression and clustering analysis. *Genomics* 2003;**82:**288-299.
96 Mody M, Cao Y, Cui Z, Tay KY, Shyong A, Shimizu E, *et al.* Genome-wide gene expression profiles of the developing mouse hippocampus. *Proc Natl Acad Sci U S A* 2001;**98:**8862-8867.
97 Bielas S, Higginbotham H, Koizumi H, Tanaka T, Gleeson JG. Cortical neuronal migration mutants suggest separate but intersecting pathways. *Annu Rev Cell Dev Biol* 2004;**20:**593-618.
98 Erraji-Benchekroun L, Underwood MD, Arango V, Galfalvy H, Pavlidis P, Smyrniotopoulos P, *et al.* Molecular aging in human prefrontal cortex is selective and continuous throughout adult life. *Biol Psychiatry* 2005;**57:**549-558.
99 Segarra J, Balenci L, Drenth T, Maina F, Lamballe F. Combined signaling through ERK, PI3K/AKT, and RAC1/p38 is required for met-triggered cortical neuron migration. *J Biol Chem* 2006;**281:**4771-4778.
100 Semeralul MO, Boutros PC, Likhodi O, Okey AB, Van Tol HH, Wong AH. Microarray analysis of the developing cortex. *J Neurobiol* 2006;**66:**1646-1658.
101 Yamashita N, Uchida Y, Ohshima T, Hirai S, Nakamura F, Taniguchi M, *et al.* Collapsin response mediator protein 1 mediates reelin signaling in cortical neuronal migration. *J Neurosci* 2006;**26:**13357-13362.
102 Jaspers NG, Raams A, Silengo MC, Wijgers N, Niedernhofer LJ, Robinson AR, *et al.* First reported patient with human ERCC1 deficiency has cerebro-oculo-facio-skeletal syndrome with a mild defect in nucleotide excision repair and severe developmental failure. *Am J Hum Genet* 2007;**80:**457-466.
103 Xie GX, Palmer PP. How regulators of G protein signaling achieve selective regulation. *J Mol Biol* 2007;**366:**349-365.
104 Choi HY, Rahmani M, Wong BW, Allahverdian S, McManus BM, Pickering JG, *et al.* ATP-binding cassette transporter A1 expression and apolipoprotein A-I binding are impaired in intima-type arterial smooth muscle cells. *Circulation* 2009;**119:**3223-3231.
105 Diaz AL, Gleeson JG. The molecular and genetic mechanisms of neocortex development. *Clin Perinatol* 2009;**36:**503-512.
106 Feng L, Cooper JA. Dual functions of Dab1 during brain development. *Mol Cell Biol* 2009;**29:**324-332.
107 Han X, Kitamoto S, Lian Q, Boisvert WA. Interleukin-10 facilitates both cholesterol uptake and efflux in macrophages. *J Biol Chem* 2009;**284:**32950-32958.
108 Lee PR, Fields RD. Regulation of myelin genes implicated in psychiatric disorders by functional activity in axons. *Front Neuroanat* 2009;**3:**4.
109 Dixon-Salazar TJ, Gleeson JG. Genetic regulation of human brain development: lessons from Mendelian diseases. *Ann N Y Acad Sci* 2010;**1214:**156-167.
110 Simó S, Jossin Y, Cooper JA. Cullin 5 regulates cortical layering by modulating the speed and duration of Dab1-dependent neuronal migration. *J Neurosci* 2010;**30:**5668-5676.
111 Khodosevich K, Seeburg PH, Monyer H. Major signaling pathways in migrating neuroblasts. *Front Mol Neurosci* 2009;**2:**7.
112 Stansberg C, Ersland KM, van der Valk P, Steen VM. Gene expression in the rat brain: high similarity but unique differences between frontomedial-, temporal- and occipital cortex. *BMC Neurosci* 2011;**12:**15.

113 Wirtz S, Schuelke M. Region-specific expression of mitochondrial complex I genes during murine brain development. *PLoS One* 2011;**6:**e18897.
114 Maglott D, Ostell J, Pruitt KD, Tatusova T. Entrez Gene: gene-centered information at NCBI. *Nucleic Acids Res* 2011;**39:**D52-D57.
115 Bayés A, van de Lagemaat LN, Collins MO, Croning MD, Whittle IR, Choudhary JS, *et al.* Characterization of the proteome, diseases and evolution of the human postsynaptic density. *Nat Neurosci* 2011;**14:**19-21.
116 Li X, Tang J, Wu Z, Zhao G, Liu C, George MS. SPECT study of Chinese schizophrenic patients suggests that cerebral hypoperfusion and laterality exist in different ethnic groups. *World J Biol Psychiatry* 2005;**6:**98-106.
117 Ruano D, Abecasis GR, Glaser B, Lips ES, Cornelisse LN, de Jong AP, *et al.* Functional gene group analysis reveals a role of synaptic heterotrimeric G proteins in cognitive ability. *Am J Hum Genet* 2010;**86:**113-125.
118 Tsai YW, Yang YR, Sun SH, Liang KC, Wang RY. Post ischemia intermittent hypoxia induces hippocampal neurogenesis and synaptic alterations and alleviates long-term memory impairment. *J Cereb Blood Flow Metab* 2013;**33:**764-773.
119 Iaci JF, Ganguly A, Finklestein SP, Parry TJ, Ren J, Saha S, *et al.* Glial growth factor 2 promotes functional recovery with treatment initiated up to 7 days after permanent focal ischemic stroke. *Neuropharmacology* 2010;**59:**640-649.
120 Liu XS, Zhang ZG, Zhang RL, Gregg S, Morris DC, Wang Y, *et al.* Stroke induces gene profile changes associated with neurogenesis and angiogenesis in adult subventricular zone progenitor cells. *J Cereb Blood Flow Metab* 2007;**27:**564-574.
121 Wiltrout C, Lang B, Yan Y, Dempsey RJ, Vemuganti R. Repairing brain after stroke: a review on post-ischemic neurogenesis. *Neurochem Int* 2007;**50:**1028-1041.
122 Jain KK. Treatment of cerebral ischemia with neurotrophic factors *MedLink Neurology* 2006;
123 Lichtenwalner RJ, Parent JM. Adult neurogenesis and the ischemic forebrain. *J Cereb Blood Flow Metab* 2006;**26:**1-20.
124 Xu Z, Croslan DR, Harris AE, Ford GD, Ford BD. Extended therapeutic window and functional recovery after intraarterial administration of neuregulin-1 after focal ischemic stroke. *J Cereb Blood Flow Metab* 2006;**26:**527-535.
125 Guan J, Bennet L, Gluckman PD, Gunn AJ. Insulin-like growth factor-1 and post-ischemic brain injury. *Prog Neurobiol* 2003;**70:**443-462.
126 Schurr A. Energy metabolism, stress hormones and neural recovery from cerebral ischemia/hypoxia. *Neurochem Int* 2002;**41:**1-8.
127 Cramer SC, Chopp M. Recovery recapitulates ontogeny. *Trends Neurosci* 2000;**23:**265-271.
128 Jensen LJ, Kuhn M, Stark M, Chaffron S, Creevey C, Muller J, *et al.* STRING 8--a global view on proteins and their functional interactions in 630 organisms. *Nucleic Acids Res* 2009;**37:**D412-D416.
129 The Gene Ontology Consortium. Gene Ontology Consortium: going forward. *Nucleic Acids Res* 2014;
130 Somanath PR, Razorenova OV, Chen J, Byzova TV. Akt1 in endothelial cell and angiogenesis. *Cell Cycle* 2006;**5:**512-518.
131 Kobayashi T, Matsumoto T, Kamata K. The PI3-K/Akt pathway: roles related to alterations in vasomotor responses in diabetic models. *J Smooth Muscle Res* 2005;**41:**283-302.
132 Shiojima I, Walsh K. Role of Akt signaling in vascular homeostasis and angiogenesis. *Circ Res* 2002;**90:**1243-1250.
133 Schleicher M, Yu J, Murata T, Derakhshan B, Atochin D, Qian L, *et al.* The Akt1-eNOS axis illustrates the specificity of kinase-substrate relationships in vivo. *Sci Signal* 2009;**2:**ra41.

134 Pore N, Jiang Z, Shu HK, Bernhard E, Kao GD, Maity A. Akt1 activation can augment hypoxia-inducible factor-1alpha expression by increasing protein translation through a mammalian target of rapamycin-independent pathway. *Mol Cancer Res* 2006;**4:**471-479.
135 Chong ZZ, Li F, Maiese K. Activating Akt and the brain's resources to drive cellular survival and prevent inflammatory injury. *Histol Histopathol* 2005;**20:**299-315.
136 Mariner DJ, Wang J, Reynolds AB. ARVCF localizes to the nucleus and adherens junction and is mutually exclusive with p120(ctn) in E-cadherin complexes. *J Cell Sci* 2000;**113 (Pt 8):**1481-1490.
137 Abbruscato TJ, Davis TP. Protein expression of brain endothelial cell E-cadherin after hypoxia/aglycemia: influence of astrocyte contact. *Brain Res* 1999;**842:**277-286.
138 Sharma HS. Neurotrophic factors attenuate microvascular permeability disturbances and axonal injury following trauma to the rat spinal cord. *Acta Neurochir Suppl* 2003;**86:**383-388.
139 Pringle AK, Sundstrom LE, Wilde GJ, Williams LR, Iannotti F. Brain-derived neurotrophic factor, but not neurotrophin-3, prevents ischaemia-induced neuronal cell death in organotypic rat hippocampal slice cultures. *Neurosci Lett* 1996;**211:**203-206.
140 Hubold C, Lang UE, Gehring H, Schultes B, Schweiger U, Peters A, *et al.* Increased serum brain-derived neurotrophic factor protein upon hypoxia in healthy young men. *J Neural Transm* 2009;**116:**1221-1225.
141 Utsugisawa K, Nagane Y, Obara D, Tohgi H. Overexpression of alpha7 nicotinic acetylcholine receptor prevents G1-arrest and DNA fragmentation in PC12 cells after hypoxia. *J Neurochem* 2002;**81:**497-505.
142 Si ML, Lee TJ. Alpha7-nicotinic acetylcholine receptors on cerebral perivascular sympathetic nerves mediate choline-induced nitrergic neurogenic vasodilation. *Circ Res* 2002;**91:**62-69.
143 Hamel E. Cholinergic modulation of the cortical microvascular bed. *Prog Brain Res* 2004;**145:**171-178.
144 Fehér A, Juhász A, Rimanóczy A, Csibri E, Kálmán J, Janka Z. Association between a genetic variant of the alpha-7 nicotinic acetylcholine receptor subunit and four types of dementia. *Dement Geriatr Cogn Disord* 2009;**28:**56-62.
145 Koto T, Takubo K, Ishida S, Shinoda H, Inoue M, Tsubota K, *et al.* Hypoxia disrupts the barrier function of neural blood vessels through changes in the expression of claudin-5 in endothelial cells. *Am J Pathol* 2007;**170:**1389-1397.
146 Kvajo M, Dhilla A, Swor DE, Karayiorgou M, Gogos JA. Evidence implicating the candidate schizophrenia/bipolar disorder susceptibility gene G72 in mitochondrial function. *Mol Psychiatry* 2008;**13:**685-696.
147 Nixon DC, Prust MJ, Sambataro F, Tan HY, Mattay VS, Weinberger DR, *et al.* Interactive effects of DAOA (G72) and catechol-O-methyltransferase on neurophysiology in prefrontal cortex. *Biol Psychiatry* 2011;**69:**1006-1008.
148 Atkin TA, MacAskill AF, Brandon NJ, Kittler JT. Disrupted in Schizophrenia-1 regulates intracellular trafficking of mitochondria in neurons. *Mol Psychiatry* 2011;**16:**122-4, 121.
149 Epstein AC, Gleadle JM, McNeill LA, Hewitson KS, O'Rourke J, Mole DR, *et al.* C. elegans EGL-9 and mammalian homologs define a family of dioxygenases that regulate HIF by prolyl hydroxylation. *Cell* 2001;**107:**43-54.
150.Marx, J. Cell biology. How cells endure low oxygen. *Science* **303,** 1454-6 (2004).doi:10.1126/science.303.5663.1454
151 Semenza GL. Hypoxia-inducible factor 1 (HIF-1) pathway. *Sci STKE* 2007;**2007:**cm8.
152 Lee JW, Bae SH, Jeong JW, Kim SH, Kim KW. Hypoxia-inducible factor (HIF-1)alpha: its protein stability and biological functions. *Exp Mol Med* 2004;**36:**1-12.
153 Jose PA, Eisner GM, Felder RA. Regulation of blood pressure by dopamine receptors. *Nephron Physiol* 2003;**95:**p19-p27.

154 Sándor P. Nervous control of the cerebrovascular system: doubts and facts. *Neurochem Int* 1999;**35:**237-259.
155 Choi JK, Chen YI, Hamel E, Jenkins BG. Brain hemodynamic changes mediated by dopamine receptors: Role of the cerebral microvasculature in dopamine-mediated neurovascular coupling. *Neuroimage* 2006;**30:**700-712.
156 Miyazaki I, Asanuma M, Diaz-Corrales FJ, Miyoshi K, Ogawa N. Direct evidence for expression of dopamine receptors in astrocytes from basal ganglia. *Brain Res* 2004;**1029:**120-123.
157 Ueda H, Baba T, Terada N, Kato Y, Fujii Y, Takayama I, *et al.* Immunolocalization of dystrobrevin in the astrocytic endfeet and endothelial cells in the rat cerebellum. *Neurosci Lett* 2000;**283:**121-124.
158 Iijima S, Masaki H, Wakayama Y, Inoue M, Jimi T, Hara H, *et al.* Immunohistochemical detection of dysbindin at the astroglial endfeet around the capillaries of mouse brain. *J Mol Histol* 2009;**40:**117-121.
159 Lyssand JS, DeFino MC, Tang XB, Hertz AL, Feller DB, Wacker JL, *et al.* Blood pressure is regulated by an alpha1D-adrenergic receptor/dystrophin signalosome. *J Biol Chem* 2008;**283:**18792-18800.
160 Guimarães S, Moura D. Vascular adrenoceptors: an update. *Pharmacol Rev* 2001;**53:**319-356.
161 Cauli B, Tong XK, Rancillac A, Serluca N, Lambolez B, Rossier J, *et al.* Cortical GABA interneurons in neurovascular coupling: relays for subcortical vasoactive pathways. *J Neurosci* 2004;**24:**8940-8949.
162 Napoleone P, Erdö S, Amenta F. Autoradiographic localization of the GABAA receptor agonist [3H]muscimol in rat cerebral vessels. *Brain Res* 1987;**423:**109-115.
163 Fergus A, Lee KS. GABAergic regulation of cerebral microvascular tone in the rat. *J Cereb Blood Flow Metab* 1997;**17:**992-1003.
164 Vaucher E, Tong XK, Cholet N, Lantin S, Hamel E. GABA neurons provide a rich input to microvessels but not nitric oxide neurons in the rat cerebral cortex: a means for direct regulation of local cerebral blood flow. *J Comp Neurol* 2000;**421:**161-171.
165 Kocharyan A, Fernandes P, Tong XK, Vaucher E, Hamel E. Specific subtypes of cortical GABA interneurons contribute to the neurovascular coupling response to basal forebrain stimulation. *J Cereb Blood Flow Metab* 2008;**28:**221-231.
166 de Plazas SF, Rapacioli M, Rodríguez Gil DJ, Vacotto M, Flores V. Acute hypoxia differentially affects the gamma-aminobutyric acid type A receptor alpha(1), alpha(2), beta(2), and gamma(2) subunit mRNA levels in the developing chick optic tectum: stage-dependent sensitivity. *J Neurosci Res* 2007;**85:**3135-3144.
167 Chi OZ, Hunter C, Liu X, Chi Y, Weiss HR. Effects of GABA(A) receptor blockade on regional cerebral blood flow and blood-brain barrier disruption in focal cerebral ischemia. *J Neurol Sci* 2011;**301:**66-70.
168 Shuaib A, Breker-Klassen MM. Inhibitory mechanisms in cerebral ischemia: a brief review. *Neurosci Biobehav Rev* 1997;**21:**219-226.
169 Schwartz-Bloom RD, Sah R. gamma-Aminobutyric acid(A) neurotransmission and cerebral ischemia. *J Neurochem* 2001;**77:**353-371.
170 Kulinskii VI, Mikhel'son GV. Additivity and independence of neuroprotective effects of GABAA and GABAB receptor agonists in complete global cerebral ischemia. *Bull Exp Biol Med* 2000;**130:**772-774.
171 Frahm C, Haupt C, Witte OW. GABA neurons survive focal ischemic injury. *Neuroscience* 2004;**127:**341-346.
172 DeFazio RA, Raval AP, Lin HW, Dave KR, Della-Morte D, Perez-Pinzon MA. GABA synapses mediate neuroprotection after ischemic and epsilonPKC preconditioning in rat hippocampal slice cultures. *J Cereb Blood Flow Metab* 2009;**29:**375-384.

173 Masson J, Emerit MB, Hamon M, Darmon M. Serotonergic signaling: multiple effectors and pleiotropic effects *Wiley Interdisciplinary Reviews: Membrane Transport and Signaling* 2012;**1:**685-713.
174 Cohen Z, Bonvento G, Lacombe P, Hamel E. Serotonin in the regulation of brain microcirculation. *Prog Neurobiol* 1996;**50:**335-362.
175 Xu T, Pandey SC. Cellular localization of serotonin(2A) (5HT(2A)) receptors in the rat brain. *Brain Res Bull* 2000;**51:**499-505.
176 Koehler RC, Roman RJ, Harder DR. Astrocytes and the regulation of cerebral blood flow. *Trends Neurosci* 2009;**32:**160-169.
177 Olesen OF, Bennike B, Dam H, Mellerup E. Association of the 5-HT2A receptor gene polymorphism 102T/C with ischemic stroke. *J Mol Neurosci* 2006;**30:**323-328.
178 Meltzer HY, Massey BW. The role of serotonin receptors in the action of atypical antipsychotic drugs. *Curr Opin Pharmacol* 2011;**11:**59-67.
179 Shibata S, Kagami-Ishi Y, Tominaga K, Kodama K, Ueki S, Watanabe S. Ischemia-induced impairment of 2-deoxyglucose uptake and CA1 field potentials in rat hippocampal slices: protection by 5-HT1A receptor agonists and 5-HT2 receptor antagonists. *Eur J Pharmacol* 1992;**229:**21-29.
180 Deane R, Schäfer W, Zimmermann HP, Mueller L, Görlich D, Prehn S, *et al.* Ran-binding protein 5 (RanBP5) is related to the nuclear transport factor importin-beta but interacts differently with RanBP1. *Mol Cell Biol* 1997;**17:**5087-5096.
181 Jäkel S, Görlich D. Importin beta, transportin, RanBP5 and RanBP7 mediate nuclear import of ribosomal proteins in mammalian cells. *EMBO J* 1998;**17:**4491-4502.
182 Mühlhäusser P, Müller EC, Otto A, Kutay U. Multiple pathways contribute to nuclear import of core histones. *EMBO Rep* 2001;**2:**690-696.
183 Shi J, Levinson DF, Duan J, Sanders AR, Zheng Y, Pe'er I, *et al.* Common variants on chromosome 6p22.1 are associated with schizophrenia. *Nature* 2009;**460:**753-757.
184 Verreault A. De novo nucleosome assembly: new pieces in an old puzzle. *Genes Dev* 2000;**14:**1430-1438.
185 Carmeliet P. Manipulating angiogenesis in medicine. *J Intern Med* 2004;**255:**538-561.
186 Carmeliet P, Jain RK. Molecular mechanisms and clinical applications of angiogenesis. *Nature* 2011;**473:**298-307.
187 Semenza GL. Hypoxia. Crosstalk Between Oxygen Sensing and the Cell Cycle Machinery. *Am J Physiol Cell Physiol* 2011;
188 Greenberg DA, Jin K. From angiogenesis to neuropathology. *Nature* 2005;**438:**954-959.
189 Clarkson AN, Liu H, Schiborra F, Shaw O, Sammut IA, Jackson DM, *et al.* Angiogenesis as a predictive marker of neurological outcome following hypoxia-ischemia. *Brain Res* 2007;**1171:**111-121.
190 Granger A, Abdullah I, Huebner F, Stout A, Wang T, Huebner T, *et al.* Histone deacetylase inhibition reduces myocardial ischemia-reperfusion injury in mice. *FASEB J* 2008;**22:**3549-3560.
191 Zhang Z, Qin X, Tong N, Zhao X, Gong Y, Shi Y, *et al.* Valproic acid-mediated neuroprotection in retinal ischemia injury via histone deacetylase inhibition and transcriptional activation. *Exp Eye Res* 2012;**94:**98-108.
192 Crosson CE, Mani SK, Husain S, Alsarraf O, Menick DR. Inhibition of histone deacetylase protects the retina from ischemic injury. *Invest Ophthalmol Vis Sci* 2010;**51:**3639-3645.
193 Blanquet PR, Mariani J, Fournier B. Temporal assessment of histone H3 phospho-acetylation and casein kinase 2 activation in dentate gyrus from ischemic rats. *Brain Res* 2009;**1302:**10-20.
194 Zager RA, Johnson AC. Renal ischemia-reperfusion injury upregulates histone-modifying enzyme systems and alters histone expression at proinflammatory/profibrotic genes. *Am J Physiol Renal Physiol* 2009;**296:**F1032-F1041.

195 Sibani S, Leclerc D, Weisberg IS, O'Ferrall E, Watkins D, Artigas C, *et al.* Characterization of mutations in severe methylenetetrahydrofolate reductase deficiency reveals an FAD-responsive mutation. *Hum Mutat* 2003;**21:**509-520.
196 Frosst P, Blom HJ, Milos P, Goyette CA, Sheppard CA, Matthews RG, *et al.* A candidate genetic risk factor for vascular disease: a common mutation in methylenetetrahydrofolate reductase *Nature Genetics* 1995;**10:**111-113.
197 UniProt Consortium. Reorganizing the protein space at the Universal Protein Resource (UniProt). *Nucleic Acids Res* 2012;**40:**D71-D75.
198 Brophy CM, Knoepp L, Xin J, Pollock JS. Functional expression of NOS 1 in vascular smooth muscle. *Am J Physiol Heart Circ Physiol* 2000;**278:**H991-H997.
199 Kavdia M, Popel AS. Contribution of nNOS- and eNOS-derived NO to microvascular smooth muscle NO exposure. *J Appl Physiol* 2004;**97:**293-301.
200 Seddon MD, Chowienczyk PJ, Brett SE, Casadei B, Shah AM. Neuronal nitric oxide synthase regulates basal microvascular tone in humans in vivo. *Circulation* 2008;**117:**1991-1996.
201 Liu VW, Huang PL. Cardiovascular roles of nitric oxide: a review of insights from nitric oxide synthase gene disrupted mice. *Cardiovasc Res* 2008;**77:**19-29.
202 Panahian N, Yoshida T, Huang PL, Hedley-Whyte ET, Dalkara T, Fishman MC, *et al.* Attenuated hippocampal damage after global cerebral ischemia in mice mutant in neuronal nitric oxide synthase. *Neuroscience* 1996;**72:**343-354.
203 Bolaños JP, Almeida A. Roles of nitric oxide in brain hypoxia-ischemia. *Biochim Biophys Acta* 1999;**1411:**415-436.
204 Uyttendaele H, Marazzi G, Wu G, Yan Q, Sassoon D, Kitajewski J. Notch4/int-3, a mammary proto-oncogene, is an endothelial cell-specific mammalian Notch gene. *Development* 1996;**122:**2251-2259.
205 Shirayoshi Y, Yuasa Y, Suzuki T, Sugaya K, Kawase E, Ikemura T, *et al.* Proto-oncogene of int-3, a mouse Notch homologue, is expressed in endothelial cells during early embryogenesis. *Genes Cells* 1997;**2:**213-224.
206 Boucher J, Gridley T, Liaw L. Molecular pathways of notch signaling in vascular smooth muscle cells. *Front Physiol* 2012;**3:**81.
207 Uyttendaele H, Closson V, Wu G, Roux F, Weinmaster G, Kitajewski J. Notch4 and Jagged-1 induce microvessel differentiation of rat brain endothelial cells. *Microvasc Res* 2000;**60:**91-103.
208 Funahashi Y, Shawber CJ, Sharma A, Kanamaru E, Choi YK, Kitajewski J. Notch modulates VEGF action in endothelial cells by inducing Matrix Metalloprotease activity. *Vasc Cell* 2011;**3:**2.
209 Liu ZJ, Shirakawa T, Li Y, Soma A, Oka M, Dotto GP, *et al.* Regulation of Notch1 and Dll4 by vascular endothelial growth factor in arterial endothelial cells: implications for modulating arteriogenesis and angiogenesis. *Mol Cell Biol* 2003;**23:**14-25.
210 Lok J, Sardi SP, Guo S, Besancon E, Ha DM, Rosell A, *et al.* Neuregulin-1 signaling in brain endothelial cells. *J Cereb Blood Flow Metab* 2009;**29:**39-43.
211 Cannella B, Pitt D, Marchionni M, Raine CS. Neuregulin and erbB receptor expression in normal and diseased human white matter. *J Neuroimmunol* 1999;**100:**233-242.
212 Carraway KL, Burden SJ. Neuregulins and their receptors. *Curr Opin Neurobiol* 1995;**5:**606-612.
213 Marmor MD, Skaria KB, Yarden Y. Signal transduction and oncogenesis by ErbB/HER receptors. *Int J Radiat Oncol Biol Phys* 2004;**58:**903-913.
214 Datta SR, Brunet A, Greenberg ME. Cellular survival: a play in three Akts. *Genes Dev* 1999;**13:**2905-2927.
215 Brunet A, Datta SR, Greenberg ME. Transcription-dependent and -independent control of neuronal survival by the PI3K-Akt signaling pathway. *Curr Opin Neurobiol* 2001;**11:**297-305.

216 Mei L, Xiong WC. Neuregulin 1 in neural development, synaptic plasticity and schizophrenia. *Nat Rev Neurosci* 2008;**9:**437-452.
217 Guo WP, Fu XG, Jiang SM, Wu JZ. Neuregulin-1 regulates the expression of Akt, Bcl-2, and Bad signaling after focal cerebral ischemia in rats. *Biochem Cell Biol* 2010;**88:**649-654.
218 Shyu WC, Lin SZ, Chiang MF, Yang HI, Thajeb P, Li H. Neuregulin-1 reduces ischemia-induced brain damage in rats. *Neurobiol Aging* 2004;**25:**935-944.
219 Li Y, Xu Z, Ford GD, Croslan DR, Cairobe T, Li Z, *et al.* Neuroprotection by neuregulin-1 in a rat model of permanent focal cerebral ischemia. *Brain Res* 2007;**1184:**277-283.
220 Croslan DR, Schoell MC, Ford GD, Pulliam JV, Gates A, Clement CM, *et al.* Neuroprotective effects of neuregulin-1 on B35 neuronal cells following ischemia. *Brain Res* 2008;**1210:**39-47.
221 Clement CM, Thomas LK, Mou Y, Croslan DR, Gibbons GH, Ford BD. Neuregulin-1 attenuates neointimal formation following vascular injury and inhibits the proliferation of vascular smooth muscle cells. *J Vasc Res* 2007;**44:**303-312.
222 Iivanainen E, Paatero I, Heikkinen SM, Junttila TT, Cao R, Klint P, *et al.* Intra- and extracellular signaling by endothelial neuregulin-1. *Exp Cell Res* 2007;**313:**2896-2909.
223 Rajasekharan S, Kennedy TE. The netrin protein family. *Genome Biol* 2009;**10:**239.
224 Park KW, Crouse D, Lee M, Karnik SK, Sorensen LK, Murphy KJ, *et al.* The axonal attractant Netrin-1 is an angiogenic factor. *Proc Natl Acad Sci U S A* 2004;**101:**16210-16215.
225 Zhang H, Zhang C. An old dog learns a new trick: netrin-1 in cardioprotection *J Mol Cell Cardiol* 2010;**48:**1033-1035.
226 Wu TW, Li WW, Li H. Netrin-1 attenuates ischemic stroke-induced apoptosis. *Neuroscience* 2008;**156:**475-482.
227 Carmeliet P. Blood vessels and nerves: common signals, pathways and diseases. *Nat Rev Genet* 2003;**4:**710-720.
228 Meerabux JM, Ohba H, Fukasawa M, Suto Y, Aoki-Suzuki M, Nakashiba T, *et al.* Human netrin-G1 isoforms show evidence of differential expression. *Genomics* 2005;**86:**112-116.
229 Sun GY, Xu J, Jensen MD, Yu S, Wood WG, González FA, *et al.* Phospholipase A2 in astrocytes: responses to oxidative stress, inflammation, and G protein-coupled receptor agonists. *Mol Neurobiol* 2005;**31:**27-41.
230 Gijón MA, Leslie CC. Regulation of arachidonic acid release and cytosolic phospholipase A2 activation. *J Leukoc Biol* 1999;**65:**330-336.
231 Nadalin S, Buretić-Tomljanović A, Rubesa G, Tomljanović D, Gudelj L. Niacin skin flush test: a research tool for studying schizophrenia. *Psychiatr Danub* 2010;**22:**14-27.
232 Bhattacharya B, Williams R, Latif ML, Roberts RE. Role of cytosolic phospholipase A2 in the enhancement of alpha2-adrenoceptor-mediated vasoconstriction by the thromboxane-mimetic U46619 in the porcine isolated ear artery: comparison with vasopressin-enhanced responses. *Biochem Pharmacol* 2005;**70:**1200-1210.
233 Gabryel B, Chalimoniuk M, Stolecka A, Langfort J. Activation of cPLA2 and sPLA2 in astrocytes exposed to simulated ischemia in vitro. *Cell Biol Int* 2007;**31:**958-965.
234 Hudson CJ, Lin A, Cogan S, Cashman F, Warsh JJ. The niacin challenge test: clinical manifestation of altered transmembrane signal transduction in schizophrenia? *Biol Psychiatry* 1997;**41:**507-513.
235 Tavares H, Yacubian J, Talib LL, Barbosa NR, Gattaz WF. Increased phospholipase A2 activity in schizophrenia with absent response to niacin. *Schizophr Res* 2003;**61:**1-6.
236 Messamore E, Hoffman WF, Janowsky A. The niacin skin flush abnormality in schizophrenia: a quantitative dose-response study. *Schizophr Res* 2003;**62:**251-258.
237 Chang SS, Liu CM, Lin SH, Hwu HG, Hwang TJ, Liu SK, *et al.* Impaired flush response to niacin skin patch among schizophrenia patients and their nonpsychotic relatives: the effect of genetic loading. *Schizophr Bull* 2009;**35:**213-221.

238 Attwell D, Buchan AM, Charpak S, Lauritzen M, Macvicar BA, Newman EA. Glial and neuronal control of brain blood flow. *Nature* 2010;**468:**232-243.
239 Gordon GR, Mulligan SJ, MacVicar BA. Astrocyte control of the cerebrovasculature. *Glia* 2007;**55:**1214-1221.
240 Iliff JJ, Jia J, Nelson J, Goyagi T, Klaus J, Alkayed NJ. Epoxyeicosanoid signaling in CNS function and disease. *Prostaglandins Other Lipid Mediat* 2010;**91:**68-84.
241 Pratt PF, Medhora M, Harder DR. Mechanisms regulating cerebral blood flow as therapeutic targets. *Curr Opin Investig Drugs* 2004;**5:**952-956.
242 Liu YV, Hubbi ME, Pan F, McDonald KR, Mansharamani M, Cole RN, *et al.* Calcineurin promotes hypoxia-inducible factor 1alpha expression by dephosphorylating RACK1 and blocking RACK1 dimerization. *J Biol Chem* 2007;**282:**37064-37073.
243 Morioka M, Hamada J, Ushio Y, Miyamoto E. Potential role of calcineurin for brain ischemia and traumatic injury. *Prog Neurobiol* 1999;**58:**1-30.
244 Bender HU, Almashanu S, Steel G, Hu CA, Lin WW, Willis A, *et al.* Functional consequences of PRODH missense mutations. *Am J Hum Genet* 2005;**76:**409-420.
245 Delwing D, Delwing D, Chiarani F, Kurek AG, Wyse AT. Proline reduces brain cytochrome c oxidase: prevention by antioxidants. *Int J Dev Neurosci* 2007;**25:**17-22.
246 Ferreira AG, Lima DD, Delwing D, Mackedanz V, Tagliari B, Kolling J, *et al.* Proline impairs energy metabolism in cerebral cortex of young rats. *Metab Brain Dis* 2010;**25:**161-168.
247 Underwood JG, Boutz PL, Dougherty JD, Stoilov P, Black DL. Homologues of the Caenorhabditis elegans Fox-1 protein are neuronal splicing regulators in mammals. *Mol Cell Biol* 2005;**25:**10005-10016.
248 Dunn KM, Nelson MT. Potassium channels and neurovascular coupling. *Circ J* 2010;**74:**608-616.
249 Leis JA, Bekar LK, Walz W. Potassium homeostasis in the ischemic brain. *Glia* 2005;**50:**407-416.
250 Félétou M. Calcium-activated potassium channels and endothelial dysfunction: therapeutic options? *Br J Pharmacol* 2009;**156:**545-562.
251 Ehler E, Karlhuber G, Bauer HC, Draeger A. Heterogeneity of smooth muscle-associated proteins in mammalian brain microvasculature. *Cell Tissue Res* 1995;**279:**393-403.
252 Loufrani L, Dubroca C, You D, Li Z, Levy B, Paulin D, *et al.* Absence of dystrophin in mice reduces NO-dependent vascular function and vascular density: total recovery after a treatment with the aminoglycoside gentamicin. *Arterioscler Thromb Vasc Biol* 2004;**24:**671-676.
253 Bansal G, Druey KM, Xie Z. R4 RGS proteins: regulation of G-protein signaling and beyond. *Pharmacol Ther* 2007;**116:**473-495.
254 Hepler JR. Emerging roles for RGS proteins in cell signalling. *Trends Pharmacol Sci* 1999;**20:**376-382.
255 Ruiz de Azua I, Scarselli M, Rosemond E, Gautam D, Jou W, Gavrilova O, *et al.* RGS4 is a negative regulator of insulin release from pancreatic beta-cells in vitro and in vivo. *Proc Natl Acad Sci U S A* 2010;**107:**7999-8004.
256 Albig AR, Schiemann WP. Identification and characterization of regulator of G protein signaling 4 (RGS4) as a novel inhibitor of tubulogenesis: RGS4 inhibits mitogen-activated protein kinases and vascular endothelial growth factor signaling. *Mol Biol Cell* 2005;**16:**609-625.
257 De Vries L, Zheng B, Fischer T, Elenko E, Farquhar MG. The regulator of G protein signaling family *Annual Review of Pharmacology and Toxicology* 2000;**40:**235-271.
258 Hollinger S, Hepler JR. Cellular regulation of RGS proteins: modulators and integrators of G protein signaling. *Pharmacol Rev* 2002;**54:**527-559.
259 Iankova I, Chavey C, Clapé C, Colomer C, Guérineau NC, Grillet N, *et al.* Regulator of G protein signaling-4 controls fatty acid and glucose homeostasis. *Endocrinology* 2008;**149:**5706-5712.

260 Bastin G, Heximer SP. Intracellular regulation of heterotrimeric G-protein signaling modulates vascular smooth muscle cell contraction. *Arch Biochem Biophys* 2011;**510:**182-189.
261 Wieland T, Mittmann C. Regulators of G-protein signalling: multifunctional proteins with impact on signalling in the cardiovascular system. *Pharmacol Ther* 2003;**97:**95-115.
262 Siedlecki AM, Jin X, Thomas W, Hruska KA, Muslin AJ. RGS4, a GTPase activator, improves renal function in ischemia-reperfusion injury. *Kidney Int* 2011;**80:**263-271.
263 Gainetdinov RR, Caron MG. Monoamine transporters: from genes to behavior. *Annu Rev Pharmacol Toxicol* 2003;**43:**261-284.
264 Eriksen J, Jørgensen TN, Gether U. Regulation of dopamine transporter function by protein-protein interactions: new discoveries and methodological challenges. *J Neurochem* 2010;**113:**27-41.
265 Schmitt KC, Reith ME. Regulation of the dopamine transporter: aspects relevant to psychostimulant drugs of abuse. *Ann N Y Acad Sci* 2010;**1187:**316-340.
266 Kaufman MJ, Levin JM, Ross MH, Lange N, Rose SL, Kukes TJ, *et al.* Cocaine-induced cerebral vasoconstriction detected in humans with magnetic resonance angiography. *JAMA* 1998;**279:**376-380.
267 Petitti DB, Sidney S, Quesenberry C, Bernstein A. Stroke and cocaine or amphetamine use. *Epidemiology* 1998;**9:**596-600.
268 Rezkalla SH, Kloner RA. Cocaine-induced acute myocardial infarction. *Clin Med Res* 2007;**5:**172-176.
269 Bogen IL, Haug KH, Roberg B, Fonnum F, Walaas SI. The importance of synapsin I and II for neurotransmitter levels and vesicular storage in cholinergic, glutamatergic and GABAergic nerve terminals. *Neurochem Int* 2009;**55:**13-21.
270 Valtorta F, Benfenati F, Greengard P, Conricode KM, Brewer KA, Exton JH, *et al.* Structure and function of the synapsins. *Structure* 1992;**267:**
271 Hilfiker S, Pieribone VA, Czernik AJ, Kao HT, Augustine GJ, Greengard P. Synapsins as regulators of neurotransmitter release. *Philos Trans R Soc Lond B Biol Sci* 1999;**354:**269-279.
272 Villanueva M, Thornley K, Augustine GJ, Wightman RM. Synapsin II negatively regulates catecholamine release. *Brain Cell Biol* 2006;**35:**125-136.
273 Schmidtko A, Luo C, Gao W, Geisslinger G, Kuner R, Tegeder I. Genetic deletion of synapsin II reduces neuropathic pain due to reduced glutamate but increased GABA in the spinal cord dorsal horn. *Pain* 2008;**139:**632-643.
274 Nishizawa Y. Glutamate release and neuronal damage in ischemia. *Life Sci* 2001;**69:**369-381.
275 Hicks DG, Janarthanan BR, Vardarajan R, Kulkarni SA, Khoury T, Dim D, *et al.* The expression of TRMT2A, a novel cell cycle regulated protein, identifies a subset of breast cancer patients with HER2 over-expression that are at an increased risk of recurrence. *BMC Cancer* 2010;**10:**108.
276 Guarguaglini G, Battistoni A, Pittoggi C, Di Matteo G, Di Fiore B, Lavia P. Expression of the murine RanBP1 and Htf9-c genes is regulated from a shared bidirectional promoter during cell cycle progression. *Biochem J* 1997;**325 (Pt 1):**277-286.
277 Di Matteo G, Salerno M, Guarguaglini G, Di Fiore B, Palitti F, Lavia P. Interactions with single-stranded and double-stranded DNA-binding factors and alternative promoter conformation upon transcriptional activation of the Htf9-a/RanBP1 and Htf9-c genes. *J Biol Chem* 1998;**273:**495-505.
278 Waltenberger J. Modulation of growth factor action: implications for the treatment of cardiovascular diseases. *Circulation* 1997;**96:**4083-4094.
279 Antoine N, Greimers R, De Roanne C, Kusaka M, Heinen E, Simar LJ, *et al.* AGM-1470, a potent angiogenesis inhibitor, prevents the entry of normal but not transformed endothelial cells into the G1 phase of the cell cycle. *Cancer Res* 1994;**54:**2073-2076.

280 Griffioen AW, Molema G. Angiogenesis: potentials for pharmacologic intervention in the treatment of cancer, cardiovascular diseases, and chronic inflammation. *Pharmacol Rev* 2000;**52:**237-268.
281 Sriram K, O'Callaghan JP. Divergent roles for tumor necrosis factor-alpha in the brain. *J Neuroimmune Pharmacol* 2007;**2:**140-153.
282 Tuttolomondo A, Di Raimondo D, di Sciacca R, Pinto A, Licata G. Inflammatory cytokines in acute ischemic stroke. *Curr Pharm Des* 2008;**14:**3574-3589.
283 Takano R, Hisahara S, Namikawa K, Kiyama H, Okano H, Miura M. Nerve growth factor protects oligodendrocytes from tumor necrosis factor-alpha-induced injury through Akt-mediated signaling mechanisms. *J Biol Chem* 2000;**275:**16360-16365.
284 Dan HC, Baldwin AS. Differential involvement of IkappaB kinases alpha and beta in cytokine- and insulin-induced mammalian target of rapamycin activation determined by Akt. *J Immunol* 2008;**180:**7582-7589.
285 Farooqui AA, Ong WY, Horrocks LA. Inhibitors of brain phospholipase A2 activity: their neuropharmacological effects and therapeutic importance for the treatment of neurologic disorders. *Pharmacol Rev* 2006;**58:**591-620.
286 Carmignoto G, Gómez-Gonzalo M. The contribution of astrocyte signalling to neurovascular coupling. *Brain Res Rev* 2010;**63:**138-148.
287 Wang L, Chopp M, Teng H, Bolz M, Francisco MA, Francisco MÃ, *et al.* Tumor necrosis factor α primes cerebral endothelial cells for erythropoietin-induced angiogenesis. *J Cereb Blood Flow Metab* 2011;**31:**640-647.
288 Salceda S, Caro J. Hypoxia-inducible factor 1alpha (HIF-1alpha) protein is rapidly degraded by the ubiquitin-proteasome system under normoxic conditions. Its stabilization by hypoxia depends on redox-induced changes. *J Biol Chem* 1997;**272:**22642-22647.
289 Laughner E, Taghavi P, Chiles K, Mahon PC, Semenza GL. HER2 (neu) signaling increases the rate of hypoxia-inducible factor 1alpha (HIF-1alpha) synthesis: novel mechanism for HIF-1-mediated vascular endothelial growth factor expression. *Mol Cell Biol* 2001;**21:**3995-4004.
290 Allard L, Turck N, Burkhard PR, Walter N, Rosell A, Gex-Fabry M, *et al.* Ubiquitin fusion degradation protein 1 as a blood marker for the early diagnosis of ischemic stroke. *Biomark Insights* 2007;**2:**155-164.
291 Maynard TM, Meechan DW, Dudevoir ML, Gopalakrishna D, Peters AZ, Heindel CC, *et al.* Mitochondrial localization and function of a subset of 22q11 deletion syndrome candidate genes. *Mol Cell Neurosci* 2008;**39:**439-451.
292 Kanehisa M, Goto S. KEGG: kyoto encyclopedia of genes and genomes. *Nucleic Acids Res* 2000;**28:**27-30.
293 Fromer M, Pocklington AJ, Kavanagh DH, Williams HJ, Dwyer S, Gormley P, *et al.* De novo mutations in schizophrenia implicate synaptic networks. *Nature* 2014;**506:**179-184.
294 Zhao Z, Xu J, Chen J, Kim S, Reimers M, Bacanu SA, *et al.* Transcriptome sequencing and genome-wide association analyses reveal lysosomal function and actin cytoskeleton remodeling in schizophrenia and bipolar disorder. *Mol Psychiatry* 2014;
295 Fels J, Jeggle P, Kusche-Vihrog K, Oberleithner H. Cortical actin nanodynamics determines nitric oxide release in vascular endothelium. *PLoS One* 2012;**7:**e41520.
296 Tansey KE, Owen MJ, O'Donovan MC. Schizophrenia genetics: building the foundations of the future. *Schizophr Bull* 2015;**41:**15-19.
297 Mi H, Lazareva-Ulitsky B, Loo R, Kejariwal A, Vandergriff J, Rabkin S, *et al.* The PANTHER database of protein families, subfamilies, functions and pathways. *Nucleic Acids Res* 2005;**33:**D284-D288.
298 Huang da W, Sherman BT, Lempicki RA. Systematic and integrative analysis of large gene lists using DAVID bioinformatics resources. *Nat Protoc* 2009;**4:**44-57.

299R Core Team (2014) *R: A language and environment for statistical computing.* R Foundation for Statistical Computing, Vienna, Austria. URL http://www.R-project.org/
300 Reddy DS. Physiological role of adrenal deoxycorticosterone-derived neuroactive steroids in stress-sensitive conditions. *Neuroscience* 2006;**138:**911-920.
301 Virgin CE, Ha TP, Packan DR, Tombaugh GC, Yang SH, Horner HC, *et al.* Glucocorticoids inhibit glucose transport and glutamate uptake in hippocampal astrocytes: implications for glucocorticoid neurotoxicity. *J Neurochem* 1991;**57:**1422-1428.
302 Gordan GS, Bentinck RC, Eisenberg E. The influence of steroids on cerebral metabolism *Annals of the New York Academy of Sciences* 1951;**54:**575-607.
303 Chang JY, Hardebo JE, Owman C. Kinetic studies on uptake of serotonin and noradrenaline into pial arteries of rats. *J Cereb Blood Flow Metab* 1990;**10:**22-31.
304 Feenstra MG. Dopamine and noradrenaline release in the prefrontal cortex in relation to unconditioned and conditioned stress and reward. *Prog Brain Res* 2000;**126:**133-163.
305 Drake CT, Iadecola C. The role of neuronal signaling in controlling cerebral blood flow. *Brain Lang* 2007;**102:**141-152.
306 González MI, Robinson MB. Neurotransmitter transporters: why dance with so many partners? *Curr Opin Pharmacol* 2004;**4:**30-35.
307 Garcia BG, Wei Y, Moron JA, Lin RZ, Javitch JA, Galli A. Akt is essential for insulin modulation of amphetamine-induced human dopamine transporter cell-surface redistribution. *Mol Pharmacol* 2005;**68:**102-109.
308 Apparsundaram S, Sung U, Price RD, Blakely RD. Trafficking-dependent and -independent pathways of neurotransmitter transporter regulation differentially involving p38 mitogen-activated protein kinase revealed in studies of insulin modulation of norepinephrine transport in SK-N-SH cells. *J Pharmacol Exp Ther* 2001;**299:**666-677.
309 Dimmeler S, Fleming I, Fisslthaler B, Hermann C, Busse R, Zeiher AM. Activation of nitric oxide synthase in endothelial cells by Akt-dependent phosphorylation. *Nature* 1999;**399:**601-605.
310 Gatson JW, Kaur P, Singh M. Dihydrotestosterone differentially modulates the mitogen-activated protein kinase and the phosphoinositide 3-kinase/Akt pathways through the nuclear and novel membrane androgen receptor in C6 cells. *Endocrinology* 2006;**147:**2028-2034.
311 Bishop JD, Nien WL, Dauphinee SM, Too CK. Prolactin activates mammalian target-of-rapamycin through phosphatidylinositol 3-kinase and stimulates phosphorylation of p70S6K and 4E-binding protein-1 in lymphoma cells. *J Endocrinol* 2006;**190:**307-312.
312 Harada H, Itasaka S, Kizaka-Kondoh S, Shibuya K, Morinibu A, Shinomiya K, *et al.* The Akt/mTOR pathway assures the synthesis of HIF-1alpha protein in a glucose- and reoxygenation-dependent manner in irradiated tumors. *J Biol Chem* 2009;**284:**5332-5342.
313 Mistry M, Gillis J, Pavlidis P. Genome-wide expression profiling of schizophrenia using a large combined cohort. *Mol Psychiatry* 2013;**18:**215-225.
314 Martyn CN, Barker DJ, Osmond C. Mothers' pelvic size, fetal growth, and death from stroke and coronary heart disease in men in the UK. *Lancet* 1996;**348:**1264-1268.
315 Osmond C, Kajantie E, Forsén TJ, Eriksson JG, Barker DJ. Infant growth and stroke in adult life: the Helsinki birth cohort study. *Stroke* 2007;**38:**264-270.
316 Woodward LJ, Anderson PJ, Austin NC, Howard K, Inder TE. Neonatal MRI to predict neurodevelopmental outcomes in preterm infants. *N Engl J Med* 2006;**355:**685-694.
317 Nosarti C, Reichenberg A, Murray RM, Cnattingius S, Lambe MP, Yin L, *et al.* Preterm birth and psychiatric disorders in young adult life. *Arch Gen Psychiatry* 2012;**69:**E1-E8.
318 Meyendorf R. Psychopathology in heart disease aside from cardiac surgery: a historical perspective of cardiac psychosis. *Compr Psychiatry* 1979;**20:**326-331.
319 Nollert G, Möhnle P, Tassani-Prell P, Uttner I, Borasio GD, Schmoeckel M, *et al.* Postoperative neuropsychological dysfunction and cerebral oxygenation during cardiac surgery. *Thorac Cardiovasc Surg* 1995;**43:**260-264.

320 Siepe M, Pfeiffer T, Gieringer A, Zemann S, Benk C, Schlensak C, *et al.* Increased systemic perfusion pressure during cardiopulmonary bypass is associated with less early postoperative cognitive dysfunction and delirium. *Eur J Cardiothorac Surg* 2011;**40:**200-207.
321 Meyendorf R. [Mental and neurological disorders associated with heart operations. Pre- and postoperative studies]. *Fortschr Med* 1976;**94:**315-320.
322 Naber D, Bullinger M. Neuroendocrine and psychological variables relating to post-operative psychosis after open-heart surgery. *Psychoneuroendocrinology* 1985;**10:**315-324.
323 Yao FS, Tseng CC, Ho CY, Levin SK, Illner P. Cerebral oxygen desaturation is associated with early postoperative neuropsychological dysfunction in patients undergoing cardiac surgery. *J Cardiothorac Vasc Anesth* 2004;**18:**552-558.
324 Granberg Axèll AI, Malmros CW, Bergbom IL, Lundberg DB. Intensive care unit syndrome/delirium is associated with anemia, drug therapy and duration of ventilation treatment. *Acta Anaesthesiol Scand* 2002;**46:**726-731.
325 McGuire BE, Basten CJ, Ryan CJ, Gallagher J. Intensive care unit syndrome: a dangerous misnomer. *Arch Intern Med* 2000;**160:**906-909.
326 Kahn DA, Prohovnik I, Lucas LR, Sackeim HA. Dissociated effects of amphetamine on arousal and cortical blood flow in humans. *Biol Psychiatry* 1989;**25:**755-767.
327 Wang AM, Suojanen JN, Colucci VM, Rumbaugh CL, Hollenberg NK. Cocaine- and methamphetamine-induced acute cerebral vasospasm: an angiographic study in rabbits. *AJNR Am J Neuroradiol* 1990;**11:**1141-1146.
328 Fandino J, Sherman JD, Zuccarello M, Rapoport RM. Cocaine-induced endothelin-1-dependent spasm in rabbit basilar artery in vivo. *J Cardiovasc Pharmacol* 2003;**41:**158-161.
329 Westover AN, McBride S, Haley RW. Stroke in young adults who abuse amphetamines or cocaine: a population-based study of hospitalized patients. *Arch Gen Psychiatry* 2007;**64:**495-502.
330 De Silva DA, Wong MC, Lee MP, Chen CL, Chang HM. Amphetamine-associated ischemic stroke: clinical presentation and proposed pathogenesis. *J Stroke Cerebrovasc Dis* 2007;**16:**185-186.
331 Łysoń T, Kochanowicz J, Rutkowski R, Turek G, Lewko J. [Cerebral vasospasm in patient with hemorrhagic stroke after amphetamine intake--case report]. *Pol Merkur Lekarski* 2008;**24:**265-267.
332 Rothman RB, Baumann MH, Dersch CM, Romero DV, Rice KC, Carroll FI, *et al.* Amphetamine-type central nervous system stimulants release norepinephrine more potently than they release dopamine and serotonin. *Synapse* 2001;**39:**32-41.
333 Robertson SD, Matthies HJ, Galli A. A closer look at amphetamine-induced reverse transport and trafficking of the dopamine and norepinephrine transporters. *Mol Neurobiol* 2009;**39:**73-80.
334 Bell DS. Comparison of amphetamine psychosis and schizophrenia. *Br J Psychiatry* 1965;**111:**701-707.
335 Callaghan RC, Cunningham JK, Allebeck P, Arenovich T, Sajeev G, Remington G, *et al.* Methamphetamine Use and Schizophrenia: A Population-Based Cohort Study in California. *Am J Psychiatry* 2011;
336 Post RM. Cocaine psychoses: a continuum model. *Am J Psychiatry* 1975;**132:**225-231.
337 Snyder SH. Amphetamine psychosis: a "model" schizophrenia mediated by catecholamines. *Am J Psychiatry* 1973;**130:**61-67.
338 Nunes JV, Broderick PA. Novel research translates to clinical cases of schizophrenic and cocaine psychosis. *Neuropsychiatr Dis Treat* 2007;**3:**475-485.
339 Bramness JG, Gundersen ØH, Guterstam J, Rognli EB, Konstenius M, Løberg EM, *et al.* Amphetamine-induced psychosis--a separate diagnostic entity or primary psychosis triggered in the vulnerable? *BMC Psychiatry* 2012;**12:**221.
340 Prohovnik I, Arnold SE, Smith G, Lucas LR. Physostigmine reversal of scopolamine-induced hypofrontality. *J Cereb Blood Flow Metab* 1997;**17:**220-228.

341 Ziegler B, Tönjes W. [Scopolamine poisoning as a cause of acute paranoid hallucinatory psychoses]. *Psychiatr Prax* 1991;**18:**21-24.
342 Rubner O, Kummerhoff PW, Haase H. [An unusual case of psychosis caused by long-term administration of a scopolamine membrane patch. Paranoid hallucinogenic and delusional symptoms]. *Nervenarzt* 1997;**68:**77-79.
343 Tandon R, Mann NA, Eisner WH, Coppard N. Effect of anticholinergic medication on positive and negative symptoms in medication-free schizophrenic patients. *Psychiatry Res* 1990;**31:**235-241.
344 Ueda Y, Walker SA, Povlishock JT. Perivascular nerve damage in the cerebral circulation following traumatic brain injury. *Acta Neuropathol* 2006;**112:**85-94.
345 McAllister TW. Traumatic Brain Injury and Psychosis: What Is the Connection? *Semin Clin Neuropsychiatry* 1998;**3:**211-223.
346 Román GC. Vascular dementia: distinguishing characteristics, treatment, and prevention. *J Am Geriatr Soc* 2003;**51:**S296-S304.
347 Román GC, Kalaria RN. Vascular determinants of cholinergic deficits in Alzheimer disease and vascular dementia. *Neurobiol Aging* 2006;**27:**1769-1785.
348 Ostling S, Gustafson D, Blennow K, Börjesson-Hanson A, Waern M. Psychotic symptoms in a population-based sample of 85-year-old individuals with dementia. *J Geriatr Psychiatry Neurol* 2011;**24:**3-8.
349 Leroi I, Voulgari A, Breitner JC, Lyketsos CG. The epidemiology of psychosis in dementia. *Am J Geriatr Psychiatry* 2003;**11:**83-91.
350 Devinsky O, Petito CK, Alonso DR. Clinical and neuropathological findings in systemic lupus erythematosus: the role of vasculitis, heart emboli, and thrombotic thrombocytopenic purpura. *Ann Neurol* 1988;**23:**380-384.
351 Jung RE, Segall JM, Grazioplene RG, Qualls C, Sibbitt WL, Roldan CA. Cortical thickness and subcortical gray matter reductions in neuropsychiatric systemic lupus erythematosus. *PLoS One* 2010;**5:**e9302.
352 López-Longo FJ, Carol N, Almoguera MI, Olazarán J, Alonso-Farto JC, Ortega A, *et al.* Cerebral hypoperfusion detected by SPECT in patients with systemic lupus erythematosus is related to clinical activity and cumulative tissue damage. *Lupus* 2003;**12:**813-819.
353 Brey RL, Holliday SL, Saklad AR, Navarrete MG, Hermosillo-Romo D, Stallworth CL, *et al.* Neuropsychiatric syndromes in lupus: prevalence using standardized definitions. *Neurology* 2002;**58:**1214-1220.
354 Stern M, Robbins ES. Psychoses in Systemic Lupus Erythematosus *Arch Gen Psychiatry* 1960;205-212.
355 Foerster K, Foerster G, Glatzel J. Symptomatische Schizophrenie bei Lupus erythematodes disseminatus *Nervenarzt* 1976;**47:**265-267.
356 Kumi S, Kazuya M, Kazunobo O, Hyroo Y. A Case of CNS Lupus Associated with Symptoms of Schizophrenia *Nishinihon Journal of Dermatology* 2001;**63:**116-119.
357 Thomas DD, Navab M, Haake DA, Fogelman AM, Miller JN, Lovett MA. Treponema pallidum invades intercellular junctions of endothelial cell monolayers. *Proc Natl Acad Sci U S A* 1988;**85:**3608-3612.
358 Quist EE, Repesh LA, Zeleznikar R, Fitzgerald TJ. Interaction of Treponema pallidum with isolated rabbit capillary tissues. *Br J Vener Dis* 1983;**59:**11-20.
359 Riley BS, Oppenheimer-Marks N, Radolf JD, Norgard MV. Virulent Treponema pallidum promotes adhesion of leukocytes to human vascular endothelial cells. *Infect Immun* 1994;**62:**4622-4625.
360 Liu Y, Zhang J, Yu L, Cao F, Rao J, Li J, *et al.* A soluble epoxide hydrolase inhibitor--8-HUDE increases pulmonary vasoconstriction through inhibition of K(ATP) channels. *Pulm Pharmacol Ther* 2012;**25:**69-76.

361 Davison K, Bagley CR. Schizophrenia-like Psychoses Associated with Organic Disorders of the Central Nervous System: A Review of the Literature. Part II of the Current Problems in Neuropsychiatry *Brit J Psychiat* 1969;**Special Publication No. 4:**113-184.
362 McCall S, Henry JM, Reid AH, Taubenberger JK. Influenza RNA not detected in archival brain tissues from acute encephalitis lethargica cases or in postencephalitic Parkinson cases. *J Neuropathol Exp Neurol* 2001;**60:**696-704.
363 Anderson LL, Vilensky JA, Duvoisin RC. Review: neuropathology of acute phase encephalitis lethargica: a review of cases from the epidemic period. *Neuropathol Appl Neurobiol* 2009;**35:**462-472.
364 Menninger KA. Influenza and schizophrenia. An analysis of post-influenzal "dementia precox," as of 1918, and five years later further studies of the psychiatric aspects of influenza. 1926. *Am J Psychiatry* 1926;**5:**469-528.
365 Davison K. Schizophrenia-like psychoses associated with organic cerebral disorders: a review. *Psychiatr Dev* 1983;**1:**1-33.
366 Linszen D, van Amelsvoort T. Cannabis and psychosis: an update on course and biological plausible mechanisms. *Curr Opin Psychiatry* 2007;**20:**116-120.
367 Boydell J, Dean K, Dutta R, Giouroukou E, Fearon P, Murray R. A comparison of symptoms and family history in schizophrenia with and without prior cannabis use: implications for the concept of cannabis psychosis. *Schizophr Res* 2007;**93:**203-210.
368 Arendt M, Mortensen PB, Rosenberg R, Pedersen CB, Waltoft BL. Familial predisposition for psychiatric disorder: comparison of subjects treated for cannabis-induced psychosis and schizophrenia. *Arch Gen Psychiatry* 2008;**65:**1269-1274.
369 van Os J. Cannabis Use and Psychosis: A Longitudinal Population-based Study *American Journal of Epidemiology* 2002;**156:**319-327.
370 Walterfang M, Wood SJ, Velakoulis D, Copolov D, Pantelis C. Diseases of white matter and schizophrenia-like psychosis. *Aust N Z J Psychiatry* 2005;**39:**746-756.
371 Trapp BD, Nave KA. Multiple sclerosis: an immune or neurodegenerative disorder? *Annu Rev Neurosci* 2008;**31:**247-269.
372 Yadav R, Zigmond AS. Temporal lobe lesions and psychosis in multiple sclerosis. *BMJ Case Rep* 2010;**2010:**
373 Vafai SB, Mootha VK. Mitochondrial disorders as windows into an ancient organelle. *Nature* 2012;**491:**374-383.
374 Anglin RE, Tarnopolsky MA, Mazurek MF, Rosebush PI. The psychiatric presentation of mitochondrial disorders in adults. *J Neuropsychiatry Clin Neurosci* 2012;**24:**394-409.
375 Craddock N, Owen MJ. The Kraepelinian dichotomy - going, going... but still not gone. *Br J Psychiatry* 2010;**196:**92-95.
376 Cardno AG, Owen MJ. Genetic relationships between schizophrenia, bipolar disorder, and schizoaffective disorder. *Schizophr Bull* 2014;**40:**504-515.
377 Marenco S, Weinberger DR. The neurodevelopmental hypothesis of schizophrenia: following a trail of evidence from cradle to grave. *Dev Psychopathol* 2000;**12:**501-527.
378: Weinberger DR, Levitt P. Neurodevelopmental origins of schizophrenia. In Weinberger DR, Harrison P (eds) *Schizophrenia.* Wiley-Blackwell, Chichester2011, pp 393-412.
Weinberger DR, Harrison PJ (eds.) Schizophrenia. Wiley-Blackwell: Chichester, 2011.
380 Berrios GE. Disorientation states and psychiatry. *Compr Psychiatry* 1982;**23:**479-491.
381 Johnstone EC, Crow TJ, Frith CD, Stevens M, Kreel L, Husband J. The dementia of dementia praecox. *Acta Psychiatr Scand* 1978;**57:**305-324.
382 Goldberg TE, Weinberger DR, Berman KF, Pliskin NH, Podd MH. Further evidence for dementia of the prefrontal type in schizophrenia? A controlled study of teaching the Wisconsin Card Sorting Test. *Arch Gen Psychiatry* 1987;**44:**1008-1014.

383 Gabriel A. Disorientation in chronic psychiatric patients *Canadian journal of psychiatry* 1997;**42:**864-868.
384 Mitelman SA, Buchsbaum MS. Very poor outcome schizophrenia: clinical and neuroimaging aspects. *Int Rev Psychiatry* 2007;**19:**345-357.
385 Kumral E, Gulluoglu H, Dramali B. Thalamic chronotaraxis: isolated time disorientation. *J Neurol Neurosurg Psychiatry* 2007;**78:**880-882.
386 Mwaniki MK, Atieno M, Lawn JE, Newton CR. Long-term neurodevelopmental outcomes after intrauterine and neonatal insults: a systematic review. *Lancet* 2012;**379:**445-452.
387 Hennington BS, Alexander BT. Linking intrauterine growth restriction and blood pressure: insight into the human origins of cardiovascular disease. *Circulation* 2013;**128:**2179-2180.
388 Gaillard R, Steegers EA, Tiemeier H, Hofman A, Jaddoe VW. Placental vascular dysfunction, fetal and childhood growth, and cardiovascular development: the generation R study. *Circulation* 2013;**128:**2202-2210.
389 Barker DJ. Coronary heart disease: a disorder of growth. *Horm Res* 2003;**59 Suppl 1:**35-41.
390 Lawlor DA, Ronalds G, Clark H, Smith GD, Leon DA. Birth weight is inversely associated with incident coronary heart disease and stroke among individuals born in the 1950s: findings from the Aberdeen Children of the 1950s prospective cohort study. *Circulation* 2005;**112:**1414-1418.
391 Barker DJ. Intrauterine programming of coronary heart disease and stroke. *Acta Paediatr Suppl* 1997;**423:**178-82; discussion 183.
392 Goldstein LB, Adams R, Alberts MJ, Appel LJ, Brass LM, Bushnell CD, *et al.* Primary prevention of ischemic stroke: a guideline from the American Heart Association/American Stroke Association Stroke Council: cosponsored by the Atherosclerotic Peripheral Vascular Disease Interdisciplinary Working Group; Cardiovascular Nursing Council; Clinical Cardiology Council; Nutrition, Physical Activity, and Metabolism Council; and the Quality of Care and Outcomes Research Interdisciplinary Working Group. *Circulation* 2006;**113:**e873-e923.
393 Rantakallio P, von Wendt L, Koivu M. Prognosis of perinatal brain damage: a prospective study of a one year birth cohort of 12,000 children. *Early Hum Dev* 1987;**15:**75-84.
394 Jones PB, Rantakallio P, Hartikainen AL, Isohanni M, Sipila P. Schizophrenia as a long-term outcome of pregnancy, delivery, and perinatal complications: a 28-year follow-up of the 1966 north Finland general population birth cohort. *Am J Psychiatry* 1998;**155:**355-364.
395 Baron JC. Perfusion thresholds in human cerebral ischemia: historical perspective and therapeutic implications. *Cerebrovasc Dis* 2001;**11 Suppl 1:**2-8.
396 Marchal G, Furlan M, Beaudouin V, Rioux P, Hauttement JL, Serrati C, *et al.* Early spontaneous hyperperfusion after stroke. A marker of favourable tissue outcome? *Brain* 1996;**119 (Pt 2):**409-419.
397 Sabri O, Erkwoh R, Schreckenberger M, Cremerius U, Schulz G, Dickmann C, *et al.* Regional cerebral blood flow and negative/positive symptoms in 24 drug-naive schizophrenics. *J Nucl Med* 1997;**38:**181-188.
398 Sabri O, Erkwoh R, Schreckenberger M, Owega A, Sass H, Buell U. Correlation of positive symptoms exclusively to hyperperfusion or hypoperfusion of cerebral cortex in never-treated schizophrenics. *Lancet* 1997;**349:**1735-1739.
399 Scheef L, Manka C, Daamen M, Kühn KU, Maier W, Schild HH, *et al.* Resting-state perfusion in nonmedicated schizophrenic patients: a continuous arterial spin-labeling 3.0-T MR study. *Radiology* 2010;**256:**253-260.
400 Heiss WD. Flow thresholds of functional and morphological damage of brain tissue. *Stroke* 1983;**14:**329-331.
401 Heiss WD, Graf R. Therapeutic window in ischaemic stroke. Experimental concepts, neuroimaging studies and implications for pharmacological treatment *CNS Drugs* 1997;**8:**474-491.

402 Heiss WD. The ischemic penumbra: correlates in imaging and implications for treatment of ischemic stroke. The Johann Jacob Wepfer award 2011. *Cerebrovasc Dis* 2011;**32:**307-320.
403 Florence G, Guerit JM, Gueguen B. Electroencephalography (EEG) and somatosensory evoked potentials (SEP) to prevent cerebral ischaemia in the operating room. *Neurophysiol Clin* 2004;**34:**17-32.
404 Théberge J. Perfusion Magnetic Resonance Imaging in Psychiatry *Top Magn Reson Imaging* 2008;**19:**111-130.
405 Allen NC, Bagade S, McQueen MB, Ioannidis JP, Kavvoura FK, Khoury MJ, *et al.* Systematic meta-analyses and field synopsis of genetic association studies in schizophrenia: the SzGene database. *Nat Genet* 2008;**40:**827-834.
406 Ben-Shachar D. Mitochondrial dysfunction in schizophrenia: a possible linkage to dopamine. *J Neurochem* 2002;**83:**1241-1251.
407 Wood SJ, Yücel M, Pantelis C, Berk M. Neurobiology of schizophrenia spectrum disorders: the role of oxidative stress. *Ann Acad Med Singapore* 2009;**38:**396-396.
408 Ong W-Y, Sim K, Farooqui AA. Oxidative stress, antioxidant defenses, and schizophrenia *Asia-Pacific Psychiatry* 2010;**2:**180-190.
409 Prabakaran S, Swatton JE, Ryan MM, Huffaker SJ, Huang JT, Griffin JL, *et al.* Mitochondrial dysfunction in schizophrenia: evidence for compromised brain metabolism and oxidative stress. *Mol Psychiatry* 2004;**9:**684-97, 643.
410 Altar CA, Jurata LW, Charles V, Lemire A, Liu P, Bukhman Y, *et al.* Deficient hippocampal neuron expression of proteasome, ubiquitin, and mitochondrial genes in multiple schizophrenia cohorts. *Biol Psychiatry* 2005;**58:**85-96.
411 Rollins B, Martin MV, Sequeira PA, Moon EA, Morgan LZ, Watson SJ, *et al.* Mitochondrial variants in schizophrenia, bipolar disorder, and major depressive disorder. *PLoS One* 2009;**4:**e4913.
412 Jou SH, Chiu NY, Liu CS. Mitochondrial dysfunction and psychiatric disorders *Chang Gung Med J* 2009;**32:**
413 Rezin GT, Amboni G, Zugno AI, Quevedo J, Streck EL. Mitochondrial Dysfunction and Psychiatric Disorders *Neurochem Res* 2009;**34:**1021-1029.
414 Martins-de-Souza D, Harris LW, Guest PC, Bahn S. The role of energy metabolism dysfunction and oxidative stress in schizophrenia revealed by proteomics. *Antioxid Redox Signal* 2011;**15:**2067-2079.
415 Laitinen T, Koskimies S, Westman P. Foeto-maternal compatibility in HLA-DR, -DQ, and -DP loci in Finnish couples suffering from recurrent spontaneous abortions. *Eur J Immunogenet* 1993;**20:**249-258.
416 Paschen W, Mies G, Hossmann KA. Threshold relationship between cerebral blood flow, glucose utilization, and energy metabolites during development of stroke in gerbils. *Exp Neurol* 1992;**117:**325-333.
417 Zhang S, Boyd J, Delaney K, Murphy TH. Rapid reversible changes in dendritic spine structure in vivo gated by the degree of ischemia. *J Neurosci* 2005;**25:**5333-5338.
418: Bustany P, Henry JF, Sargent T, Zarifian E, Cabanis E, Collard P, Comar D. Local brain protein metabolism in dementia and schizophrenia: In vivo studies with 11C-L-methionine and positron emission tomography. In Heiss WD, Phelps MF (eds) *Positron Emission Tomography of the Brain.* Springer, 1983, pp 208-211.
419 Selemon LD, Goldman-Rakic PS. The reduced neuropil hypothesis: a circuit based model of schizophrenia. *Biol Psychiatry* 1999;**45:**17-25.
420 Garey LJ, Ong WY, Patel TS, Kanani M, Davis A, Mortimer AM, *et al.* Reduced dendritic spine density on cerebral cortical pyramidal neurons in schizophrenia. *J Neurol Neurosurg Psychiatry* 1998;**65:**446-453.

421 Glantz LA, Lewis DA. Decreased dendritic spine density on prefrontal cortical pyramidal neurons in schizophrenia *Archives of general psychiatry* 2000;**57:**65.
422 Obrenovitch TP, Garofalo O, Harris RJ, Bordi L, Ono M, Momma F, *et al.* Brain tissue concentrations of ATP, phosphocreatine, lactate, and tissue pH in relation to reduced cerebral blood flow following experimental acute middle cerebral artery occlusion. *J Cereb Blood Flow Metab* 1988;**8:**866-874.
423 Hossmann KA. Viability thresholds and the penumbra of focal ischemia. *Ann Neurol* 1994;**36:**557-565.
424 Volz HR, Riehemann S, Maurer I, Smesny S, Sommer M, Rzanny R, *et al.* Reduced phosphodiesters and high-energy phosphates in the frontal lobe of schizophrenic patients: a (31)P chemical shift spectroscopic-imaging study. *Biol Psychiatry* 2000;**47:**954-961.
425 Du F, Cooper AJ, Thida T, Sehovic S, Lukas SE, Cohen BM, *et al.* In vivo evidence for cerebral bioenergetic abnormalities in schizophrenia measured using 31P magnetization transfer spectroscopy. *JAMA Psychiatry* 2014;**71:**19-27.
426 Looney JM, Childs HM. The lactic acid and glutathione content of the blood of schizophrenic patients. *J Clin Invest* 1934;**13:**963-968.
427 Regenold WT, Phatak P, Marano CM, Sassan A, Conley RR, Kling MA. Elevated cerebrospinal fluid lactate concentrations in patients with bipolar disorder and schizophrenia: implications for the mitochondrial dysfunction hypothesis. *Biol Psychiatry* 2009;**65:**489-494.
428 Hargadine JR, Branston NM, Symon L. Central conduction time in primate brain ischemia -- a study in baboons. *Stroke* 1980;**11:**637-642.
429 Astrup J. Energy-requiring cell functions in the ischemic brain. Their critical supply and possible inhibition in protective therapy. *J Neurosurg* 1982;**56:**482-497.
430 Ford JM. Schizophrenia: the broken P300 and beyond. *Psychophysiology* 1999;**36:**667-682.
431 Jeon YW, Polich J. Meta-analysis of P300 and schizophrenia: patients, paradigms, and practical implications. *Psychophysiology* 2003;**40:**684-701.
432 Symond MP, Symond MB, Harris AW, Gordon E, Williams LM. "Gamma synchrony" in first-episode schizophrenia: a disorder of temporal connectivity? *Am J Psychiatry* 2005;**162:**459-465.
433 Uhlhaas PJ, Singer W. Abnormal neural oscillations and synchrony in schizophrenia. *Nat Rev Neurosci* 2010;**11:**100-113.
434 Manschreck TC, Maher BA, Rucklos ME, Vereen DR. Disturbed voluntary motor activity in schizophrenic disorder. *Psychol Med* 1982;**12:**73-84.
435 Rao R, Jackson S, Howard R. Primitive reflexes in cerebrovascular disease: a community study of older people with stroke and carotid stenosis. *Int J Geriatr Psychiatry* 1999;**14:**964-972.
436 Dazzan P, Murray RM. Neurological soft signs in first-episode psychosis: a systematic review. *Br J Psychiatry Suppl* 2002;**43:**s50-s57.
437 Hyde TM, Goldberg TE, Egan MF, Lener MC, Weinberger DR. Frontal release signs and cognition in people with schizophrenia, their siblings and healthy controls. *Br J Psychiatry* 2007;**191:**120-125.
438 Utena H, Ezoe T. Studies of the carbohydrate metabolism in brain tissue of schizophrenic patients: The aerobic metabolism of glucose, (in Japanese with English abstract) *Psychiatria et Neurologia Japonica* 1951;**52:**216-232.
439 Martins-de-Souza D, Maccarrone G, Wobrock T, Zerr I, Gormanns P, Reckow S, *et al.* Proteome analysis of the thalamus and cerebrospinal fluid reveals glycolysis dysfunction and potential biomarkers candidates for schizophrenia. *J Psychiatr Res* 2010;
440 English JA, Pennington K, Dunn MJ, Cotter DR. The neuroproteomics of schizophrenia. *Biol Psychiatry* 2011;**69:**163-172.
441 Astrup J, Symon L, Branston NM, Lassen NA. Cortical evoked potential and extracellular K+ and H+ at critical levels of brain ischemia. *Stroke* 1977;**8:**51-57.

442 Riederer P, Gsell W, Calza L, Franzek E, Jungkunz G, Jellinger K, *et al.* Consensus on minimal criteria of clinical and neuropathological diagnosis of schizophrenia and affective disorders for post mortem research. Report from the European Dementia and Schizophrenia Network (BIOMED I). *J Neural Transm Gen Sect* 1995;**102:**255-264.
443 Harrison PJ. The neuropathology of schizophrenia. A critical review of the data and their interpretation. *Brain* 1999;**122 (Pt 4):**593-624.
444 Rehncrona S, Mela L, Siesjö BK. Recovery of brain mitochondrial function in the rat after complete and incomplete cerebral ischemia. *Stroke* 1979;**10:**437-446.
445 Fiskum G, Murphy AN, Beal MF. Mitochondria in neurodegeneration: acute ischemia and chronic neurodegenerative diseases. *J Cereb Blood Flow Metab* 1999;**19:**351-369.
446 Li J, Ma X, Yu W, Lou Z, Mu D, Wang Y, *et al.* Reperfusion promotes mitochondrial dysfunction following focal cerebral ischemia in rats. *PLoS One* 2012;**7:**e46498.
447 Takahashi Y. AN ENZYMOLOGICAL STUDY ON BRAIN TISSUE OF SCHIZOPHRENIC PATIENTS. CARBOHYDRATE METABOLISM. PART I. GLUCOSE* *Psychiatry and Clinical Neurosciences* 1953;**7:**214-237.
448 Schurr A. Brain Anaerobic Lactate Production: A Suicide Note or a Survival Kit? *Dev Neurosci* 1998;**20:**348-357.
449 Muralikrishna Adibhatla R, Hatcher JF. Phospholipase A2, reactive oxygen species, and lipid peroxidation in cerebral ischemia. *Free Radic Biol Med* 2006;**40:**376-387.
450 Mahadik SP, Mukherjee S, Scheffer R, Correnti EE, Mahadik JS. Elevated plasma lipid peroxides at the onset of nonaffective psychosis. *Biol Psychiatry* 1998;**43:**674-679.
451 Khan MM, Evans DR, Gunna V, Scheffer RE, Parikh VV, Mahadik SP. Reduced erythrocyte membrane essential fatty acids and increased lipid peroxides in schizophrenia at the never-medicated first-episode of psychosis and after years of treatment with antipsychotics. *Schizophr Res* 2002;**58:**1-10.
452 Arvindakshan M, Sitasawad S, Debsikdar V, Ghate M, Evans D, Horrobin DF, *et al.* Essential polyunsaturated fatty acid and lipid peroxide levels in never-medicated and medicated schizophrenia patients. *Biol Psychiatry* 2003;**53:**56-64.
453 Li HC, Chen QZ, Ma Y, Zhou JF. Imbalanced free radicals and antioxidant defense systems in schizophrenia: a comparative study. *J Zhejiang Univ Sci B* 2006;**7:**981-986.
454 Huang J, Upadhyay UM, Tamargo RJ. Inflammation in stroke and focal cerebral ischemia. *Surg Neurol* 2006;**66:**232-245.
455 Famakin BM. The Immune Response to Acute Focal Cerebral Ischemia and Associated Post-stroke Immunodepression: A Focused Review. *Aging Dis* 2014;**5:**307-326.
456 Meyer U, Schwarz MJ, Müller N. Inflammatory processes in schizophrenia: a promising neuroimmunological target for the treatment of negative/cognitive symptoms and beyond. *Pharmacol Ther* 2011;**132:**96-110.
457 Miller BJ, Culpepper N, Rapaport MH. C-reactive protein levels in schizophrenia: a review and meta-analysis. *Clin Schizophr Relat Psychoses* 2014;**7:**223-230.
458 Brondani R, Rieder CR, Valente D, Araújo LF, Clausell N. Levels of vascular cell adhesion molecule-1 and endothelin-1 in ischemic stroke: a longitudinal prospective study. *Clin Biochem* 2007;**40:**282-284.
459 Papadopoulos SM, Gilbert LL, Webb RC, D'Amato CJ. Characterization of contractile responses to endothelin in human cerebral arteries: implications for cerebral vasospasm. *Neurosurgery* 1990;**26:**810-815.
460 Schwarz E, Izmailov R, Spain M, Barnes A, Mapes JP, Guest PC, *et al.* Validation of a blood-based laboratory test to aid in the confirmation of a diagnosis of schizophrenia. *Biomark Insights* 2010;**5:**39-47.
461 Sun BD, Liu HM, Nie SN. S100B protein in serum is elevated after global cerebral ischemic injury. *World J Emerg Med* 2013;**4:**165-168.

462 Rothermundt M, Ponath G, Arolt V. S100B in schizophrenic psychosis. *Int Rev Neurobiol* 2004;**59:**445-470.
463 Zhang XY, Xiu MH, Song C, Chen da C, Wu GY, Haile CN, *et al.* Increased serum S100B in never-medicated and medicated schizophrenic patients. *J Psychiatr Res* 2010;**44:**1236-1240.
464 Ghose K, Sandle L. Serum creatine kinase activity in the elderly following a stroke. *Arch Gerontol Geriatr* 1985;**4:**163-167.
465 Meltzer H. Creatine kinase and aldolase in serum: abnormality common to acute psychoses. *Science* 1968;**159:**1368-1370.
466 Swartz CM, Breen KJ. Multiple muscle enzyme release with psychiatric illness. *J Nerv Ment Dis* 1990;**178:**755-759.
467 Hermesh H, Stein D, Manor I, Shechtmann T, Blumensohn R, Meged S, *et al.* Serum creatine kinase levels in untreated hospitalized adolescents during acute psychosis. *J Am Acad Child Adolesc Psychiatry* 2002;**41:**1045-1053.
468 Kirch DG, Kaufmann CA, Papadopoulos NM, Martin B, Weinberger DR. Abnormal cerebrospinal fluid protein indices in schizophrenia. *Biol Psychiatry* 1985;**20:**1039-1046.
469 Bauer K, Kornhuber J. Blood-cerebrospinal fluid barrier in schizophrenic patients. *Eur Arch Psychiatry Neurol Sci* 1987;**236:**257-259.
470 Müller N, Ackenheil M. Immunoglobulin and albumin content of cerebrospinal fluid in schizophrenic patients: relationship to negative symptomatology. *Schizophr Res* 1995;**14:**223-228.
471 Ueno M, Tomimoto H, Akiguchi I, Wakita H, Sakamoto H. Blood-brain barrier disruption in white matter lesions in a rat model of chronic cerebral hypoperfusion. *J Cereb Blood Flow Metab* 2002;**22:**97-104.
472 Wu JS, Chen XC, Chen H, Shi YQ. A study on blood-brain barrier ultrastructural changes induced by cerebral hypoperfusion of different stages. *Neurol Res* 2006;**28:**50-58.
473 Uranova NA, Zimina IS, Vikhreva OV, Krukov NO, Rachmanova VI, Orlovskaya DD. Ultrastructural damage of capillaries in the neocortex in schizophrenia. *World J Biol Psychiatry* 2010;**11:**567-578.
474 Solenski NJ, diPierro CG, Trimmer PA, Kwan AL, Helm GA, Helms GA. Ultrastructural changes of neuronal mitochondria after transient and permanent cerebral ischemia. *Stroke* 2002;**33:**816-824.
475 Dewar D, Underhill SM, Goldberg MP. Oligodendrocytes and ischemic brain injury *Journal of Cerebral Blood Flow & Metabolism* 2003;**23:**263-274.
476 Davis KL, Stewart DG, Friedman JI, Buchsbaum M, Harvey PD, Hof PR, *et al.* White matter changes in schizophrenia: evidence for myelin-related dysfunction. *Arch Gen Psychiatry* 2003;**60:**443-456.
477 Dwork AJ, Mancevski B, Rosoklija G. White matter and cognitive function in schizophrenia. *Int J Neuropsychopharmacol* 2007;**10:**513-536.
478 Bernstein HG, Steiner J, Bogerts B. Glial cells in schizophrenia: pathophysiological significance and possible consequences for therapy. *Expert Rev Neurother* 2009;**9:**1059-1071.
479 Martins-de-Souza D. Proteome and transcriptome analysis suggests oligodendrocyte dysfunction in schizophrenia. *J Psychiatr Res* 2010;**44:**149-156.
480 Rosoklija G, Toomayan G, Ellis SP, Keilp J, Mann JJ, Latov N, *et al.* Structural abnormalities of subicular dendrites in subjects with schizophrenia and mood disorders: preliminary findings. *Arch Gen Psychiatry* 2000;**57:**349-356.
481 Falkai P, Parlapani E, Gruber O, Schmitt A. The Neuropathology of Schizophrenia: Central Role for the Hippocampus? *Advances in Schizophrenia Research 2009* 2010;149-165.
482: Harrison PJ, Lewis DA, Kleinman JE. Neuropathology of schizophrenia. In Weinberger DR, Harrison PJ (eds) *Schizophrenia.* Wiley-Blackwell, Chichester2011, pp 372-392.
483 Nuwer MR. Intraoperative electroencephalography. *J Clin Neurophysiol* 1993;**10:**437-444.

484 Bramon E, Rabe-Hesketh S, Sham P, Murray RM, Frangou S. Meta-analysis of the P300 and P50 waveforms in schizophrenia. *Schizophr Res* 2004;**70:**315-329.
485 Qiu YQ, Tang YX, Chan RC, Sun XY, He J. P300 aberration in first-episode schizophrenia patients: a meta-analysis. *PLoS One* 2014;**9:**e97794.
486 Mathalon DH, Ford JM, Pfefferbaum A. Trait and state aspects of P300 amplitude reduction in schizophrenia: a retrospective longitudinal study. *Biol Psychiatry* 2000;**47:**434-449.
487 Fano S, Behrens CJ, Heinemann U. Hypoxia suppresses kainate-induced gamma-oscillations in rat hippocampal slices. *Neuroreport* 2007;**18:**1827-1831.
488 Huchzermeyer C, Albus K, Gabriel HJ, Otáhal J, Taubenberger N, Heinemann U, *et al.* Gamma oscillations and spontaneous network activity in the hippocampus are highly sensitive to decreases in pO2 and concomitant changes in mitochondrial redox state. *J Neurosci* 2008;**28:**1153-1162.
489 Davidson LL, Heinrichs RW. Quantification of frontal and temporal lobe brain-imaging findings in schizophrenia: a meta-analysis. *Psychiatry Res* 2003;**122:**69-87.
490 Hill K, Mann L, Laws KR, Stephenson CM, Nimmo-Smith I, McKenna PJ. Hypofrontality in schizophrenia: a meta-analysis of functional imaging studies. *Acta Psychiatr Scand* 2004;**110:**243-256.
491 Novak B, Milcinski M, Grmek M, Kocmur M. Early effects of treatment on regional cerebral blood flow in first episode schizophrenia patients evaluated with 99Tc-ECD-SPECT. *Neuro Endocrinol Lett* 2005;**26:**685-689.
492 McFarland RA.*Archives of Psychology* 1932;**145:**
493 Elvevåg B, Goldberg TE. Cognitive impairment in schizophrenia is the core of the disorder. *Crit Rev Neurobiol* 2000;**14:**1-21.
494: Arango C, Carpenter WT. The schizophrenia construct: symptomatic presentation. In Weinberger DR, Harrison PJ (eds) *Schizophrenia.* Wiley-Blackwell, Chichester2011, pp 9-23.
495 Black DW. Pathological laughter: a review of the literature *The Journal of nervous and mental disease* 1982;**170:**67-71.
496 Andreasen NC. The evolving concept of schizophrenia: from Kraepelin to the present and future. *Schizophr Res* 1997;**28:**105-109.
497 Mesholam-Gately RI, Giuliano AJ, Goff KP, Faraone SV, Seidman LJ. Neurocognition in first-episode schizophrenia: a meta-analytic review. *Neuropsychology* 2009;**23:**315-336.
498 Harrison PJ. Schizophrenia: a disorder of neurodevelopment? *Curr Opin Neurobiol* 1997;**7:**285-289.
499 Moises H, Zoega T, Li L, Hood L. Genes and Neurodevelopment in Schizophrenia. DiLalla L*Behavior Genetics Principles - Principles in Development, Personality, and Psychopathology.* American Psychological Association, Washington, DC2004, pp 145-157.
500 Cannon M, Jones PB, Murray RM. Obstetric complications and schizophrenia: historical and meta-analytic review. *Am J Psychiatry* 2002;**159:**1080-1092.
501 Eriksson JG, Kajantie E, Thornburg KL, Osmond C, Barker DJ. Mother's body size and placental size predict coronary heart disease in men. *Eur Heart J* 2011;**32:**2297-2303.
502 Dalman C, Allebeck P, Cullberg J, Grunewald C, Köster M. Obstetric complications and the risk of schizophrenia: a longitudinal study of a national birth cohort. *Arch Gen Psychiatry* 1999;**56:**234-240.
503 Roberts JM, Cooper DW. Pathogenesis and genetics of pre-eclampsia. *Lancet* 2001;**357:**53-56.
504 Brown MC, Best KE, Pearce MS, Waugh J, Robson SC, Bell R. Cardiovascular disease risk in women with pre-eclampsia: systematic review and meta-analysis. *Eur J Epidemiol* 2013;**28:**1-19.
505 Abel KM, Drake R, Goldstein JM. Sex differences in schizophrenia. *Int Rev Psychiatry* 2010;**22:**417-428.

506 Barker DJ, Osmond C, Golding J, Kuh D, Wadsworth ME. Growth in utero, blood pressure in childhood and adult life, and mortality from cardiovascular disease. *British Medical Journal* 1989;**298:**564.
507 Mazumder B, Almond D, Park K, Crimmins EM, Finch CE. Lingering prenatal effects of the 1918 influenza pandemic on cardiovascular disease. *J Dev Orig Health Dis* 2010;**1:**26-34.
508 St Clair D, Xu M, Wang P, Yu Y, Fang Y, Zhang F, *et al.* Rates of adult schizophrenia following prenatal exposure to the Chinese famine of 1959-1961. *JAMA* 2005;**294:**557-562.
509 Xu MQ, Sun WS, Liu BX, Feng GY, Yu L, Yang L, *et al.* Prenatal malnutrition and adult schizophrenia: further evidence from the 1959-1961 Chinese famine. *Schizophr Bull* 2009;**35:**568-576.
510 Roseboom TJ, Painter RC, van Abeelen AF, Veenendaal MV, de Rooij SR. Hungry in the womb: what are the consequences? Lessons from the Dutch famine. *Maturitas* 2011;**70:**141-145.
511 Palmer CG. Evidence for maternal-fetal genotype incompatibility as a risk factor for schizophrenia. *J Biomed Biotechnol* 2010;**2010:**576318.
512 Wang T. Amniotic epithelium in rhesus incompatibility: light and electron microscopic examination. *Placenta* 1981;**2:**35-43.
513 Barresi G, Tuccari G, Marullo O, Chimicata S, Inferrera C. Vascular changes in placentae from cases of materno-fetal rhesus incompatibility. *Appl Pathol* 1983;**1:**310-316.
514 Davies G, Welham J, Chant D, Torrey EF, McGrath J. A systematic review and meta-analysis of Northern Hemisphere season of birth studies in schizophrenia. *Schizophr Bull* 2003;**29:**587-593.
515 McGrath JJ, Welham JL. Season of birth and schizophrenia: a systematic review and meta-analysis of data from the Southern Hemisphere. *Schizophr Res* 1999;**35:**237-242.
516 Lawlor DA, Davey Smith G, Mitchell R, Ebrahim S. Temperature at birth, coronary heart disease, and insulin resistance: cross sectional analyses of the British women's heart and health study. *Heart* 2004;**90:**381-388.
517 Refsum H, Nurk E, Smith AD, Ueland PM, Gjesdal CG, Bjelland I, *et al.* The Hordaland Homocysteine Study: a community-based study of homocysteine, its determinants, and associations with disease. *J Nutr* 2006;**136:**1731S-1740S.
518 De Meyer T, Rietzschel ER, De Buyzere ML, De Bacquer D, Van Criekinge W, De Backer GG, *et al.* Paternal age at birth is an important determinant of offspring telomere length. *Hum Mol Genet* 2007;**16:**3097-3102.
519 Brouilette S, Singh RK, Thompson JR, Goodall AH, Samani NJ. White cell telomere length and risk of premature myocardial infarction. *Arterioscler Thromb Vasc Biol* 2003;**23:**842-846.
520 Mulder EJ, Robles de Medina PG, Huizink AC, Van den Bergh BR, Buitelaar JK, Visser GH. Prenatal maternal stress: effects on pregnancy and the (unborn) child. *Early Hum Dev* 2002;**70:**3-14.
521 Seckl JR, Holmes MC. Mechanisms of disease: glucocorticoids, their placental metabolism and fetal 'programming' of adult pathophysiology. *Nat Clin Pract Endocrinol Metab* 2007;**3:**479-488.
522 Kempton MJ, Stahl D, Williams SC, DeLisi LE. Progressive lateral ventricular enlargement in schizophrenia: a meta-analysis of longitudinal MRI studies. *Schizophr Res* 2010;**120:**54-62.
523 Olabi B, Ellison-Wright I, McIntosh AM, Wood SJ, Bullmore E, Lawrie SM. Are there progressive brain changes in schizophrenia? A meta-analysis of structural magnetic resonance imaging studies. *Biol Psychiatry* 2011;**70:**88-96.
524 Barker DJ, Osmond C, Kajantie E, Eriksson JG. Growth and chronic disease: findings in the Helsinki Birth Cohort. *Ann Hum Biol* 2009;**36:**445-458.
525 Flensborg-Madsen T, Sørensen HJ, Revsbech R, Mortensen EL. Early motor developmental milestones and level of neuroticism in young adulthood: a 23-year follow-up study of the Copenhagen Perinatal Cohort. *Psychol Med* 2013;**43:**1293-1301.

526 Maier W, Minges J, Lichtermann D, Heun R, Franke P. Personality variations in healthy relatives of schizophrenics. *Schizophr Res* 1994;**12:**81-88.
527 Widiger TA, Costa Jr PT. Personality and Personality Disorders *J Abnorm Psychol* 1994;**103:**78-91.
528 Meggendorfer F. Klinische und genealogische Untersuchungen über Moral insanity *Zeitschrift für die gesamte Neurologie und Psychiatrie* 1921;**66:**208-231.
529 Eysenck HJ, Eysenck MW. *Personality and individual differences: A natural science approach* New York, 1985: Plenum Press
530 Heston LL. Psychiatric disorders in foster home reared children of schizophrenic mothers. *Br J Psychiatry* 1966;**112:**819-825.
531 Shen BJ, Avivi YE, Todaro JF, Spiro A, Laurenceau JP, Ward KD, *et al.* Anxiety characteristics independently and prospectively predict myocardial infarction in men the unique contribution of anxiety among psychologic factors. *J Am Coll Cardiol* 2008;**51:**113-119.
532 Wong ND, Reading AE. Personality correlates of type A behavior *Personality and Individual Differences* 1989;**10:**991-996.
533 Boks MP, Russo S, Knegtering R, van den Bosch RJ. The specificity of neurological signs in schizophrenia: a review. *Schizophr Res* 2000;**43:**109-116.
534 Bachmann S, Degen C, Geider FJ, Schröder J. Neurological soft signs in the clinical course of schizophrenia: results of a meta-analysis. *Front Psychiatry* 2014;**5:**185.
535 Hyde TM, Deep-Soboslay A, Iglesias B, Callicott JH, Gold JM, Meyer-Lindenberg A, *et al.* Enuresis as a premorbid developmental marker of schizophrenia. *Brain* 2008;**131:**2489-2498.
536 Fergusson DM, Horwood LJ. Nocturnal enuresis and behavioral problems in adolescence: a 15-year longitudinal study. *Pediatrics* 1994;**94:**662-668.
537 Young GC. Personality factors and the treatment of enuresis *Behav Res Ther* 1965;**3:**103-105.
538 Hellman DS, Blackman N. Enuresis, firesetting and cruelty to animals: a triad predictive of adult crime. *Am J Psychiatry* 1966;**122:**1431-1435.
539 Woodberry KA, Giuliano AJ, Seidman LJ. Premorbid IQ in schizophrenia: a meta-analytic review. *Am J Psychiatry* 2008;**165:**579-587.
540 Huber G, Gross G, Schüttler R, Linz M. Longitudinal studies of schizophrenic patients. *Schizophr Bull* 1980;**6:**592-605.
541 Urfer-Parnas A, Lykke Mortensen E, Saebye D, Parnas J. Pre-morbid IQ in mental disorders: a Danish draft-board study of 7486 psychiatric patients. *Psychol Med* 2010;**40:**547-556.
542 Maccabe JH, Brébion G, Reichenberg A, Ganguly T, McKenna PJ, Murray RM, *et al.* Superior intellectual ability in schizophrenia: Neuropsychological characteristics. *Neuropsychology* 2012;**26:**181-190.
543: von Stumm S, Chamorro-Premuzic T, Ackerman PL. Re-Visiting Intelligence– Personality Associations - Vindicating Intellectual Investment. In Chamorro-Premuzic T, von Stumm S, Furnham A (eds) *The Wiley-Blackwell Handbook of Individual Differences.* Blackwell Publishing, 2011, pp 217-241.
544 Saha S, Chant D, Welham J, McGrath J. A systematic review of the prevalence of schizophrenia. *PLoS Med* 2005;**2:**e141.
545 Mondelli V, Pariante CM. Adding aspirin to antipsychotics reduces psychopathology in adults with schizophrenia spectrum disorders. *Evid Based Ment Health* 2010;**13:**122.
546 Field TS, Benavente OR. Current status of antiplatelet agents to prevent stroke. *Curr Neurol Neurosci Rep* 2011;**11:**6-14.
547 Essali A, Al-Haj Haasan N, Li C, Rathbone J. Clozapine versus typical neuroleptic medication for schizophrenia. *Cochrane Database Syst Rev* 2009;CD000059.
548 Melnik T, Soares BG, Puga ME, Atallah AN. Efficacy and safety of atypical antipsychotic drugs (quetiapine, risperidone, aripiprazole and paliperidone) compared with placebo or typical

antipsychotic drugs for treating refractory schizophrenia: overview of systematic reviews. *Sao Paulo Med J* 2010;**128:**141-166.
549 Miller DD, Rezai K, Alliger R, Andreasen NC. The effect of antipsychotic medication on relative cerebral blood perfusion in schizophrenia: assessment with technetium-99m hexamethyl-propyleneamine oxime single photon emission computed tomography. *Biol Psychiatry* 1997;**41:**550-559.
550 Goozée R, Handley R, Kempton MJ, Dazzan P. A systematic review and meta-analysis of the effects of antipsychotic medications on regional cerebral blood flow (rCBF) in schizophrenia: association with response to treatment. *Neurosci Biobehav Rev* 2014;**43:**118-136.
551 Müller N, Riedel M, Scheppach C, Brandstätter B, Sokullu S, Krampe K, *et al.* Beneficial antipsychotic effects of celecoxib add-on therapy compared to risperidone alone in schizophrenia. *Am J Psychiatry* 2002;**159:**1029-1034.
552 Akhondzadeh S, Tabatabaee M, Amini H, Ahmadi Abhari SA, Abbasi SH, Behnam B. Celecoxib as adjunctive therapy in schizophrenia: a double-blind, randomized and placebo-controlled trial. *Schizophr Res* 2007;**90:**179-185.
553 Müller N, Krause D, Dehning S, Musil R, Schennach-Wolff R, Obermeier M, *et al.* Celecoxib treatment in an early stage of schizophrenia: results of a randomized, double-blind, placebo-controlled trial of celecoxib augmentation of amisulpride treatment. *Schizophr Res* 2010;**121:**118-124.
554 Sommer IE, de Witte L, Begemann M, Kahn RS. Nonsteroidal anti-inflammatory drugs in schizophrenia: ready for practice or a good start? a meta-analysis. *J Clin Psychiatry* 2011;
555 López-Villodres JA, De La Cruz JP, Muñoz-Marin J, Guerrero A, Reyes JJ, González-Correa JA. Cytoprotective effect of nonsteroidal antiinflammatory drugs in rat brain slices subjected to reoxygenation after oxygen-glucose deprivation. *Eur J Pharm Sci* 2012;
556 Tharyan P, Adams CE. Electroconvulsive therapy for schizophrenia. *Cochrane Database Syst Rev* 2005;CD000076.
557 Leonhardt G, de Greiff A, Weber J, Ludwig T, Wiedemayer H, Forsting M, *et al.* Brain perfusion following single seizures. *Epilepsia* 2005;**46:**1943-1949.
558 Hogan RE, Cook MJ, Binns DW, Desmond PM, Kilpatrick CJ, Murrie VL, *et al.* Perfusion patterns in postictal 99mTc-HMPAO SPECT after coregistration with MRI in patients with mesial temporal lobe epilepsy *Journal of Neurology, Neurosurgery & Psychiatry* 1997;**63:**235.
559 Galynker II, Weiss J, Ongseng F, Finestone H. ECT treatment and cerebral perfusion in Catatonia. *J Nucl Med* 1997;**38:**251-254.
560 Ehrenreich H, Hinze-Selch D, Stawicki S, Aust C, Knolle-Veentjer S, Wilms S, *et al.* Improvement of cognitive functions in chronic schizophrenic patients by recombinant human erythropoietin. *Mol Psychiatry* 2007;**12:**206-220.
561 Shafi NI, Andresen J, Marrelli SP, Bryan RM. Erythropoietin potentiates EDHF-mediated dilations in rat middle cerebral arteries. *J Neurotrauma* 2008;**25:**257-265.
562 Chong ZZ, Maiese K. Erythropoietin involves the phosphatidylinositol 3-kinase pathway, 14-3-3 protein and FOXO3a nuclear trafficking to preserve endothelial cell integrity. *Br J Pharmacol* 2007;**150:**839-850.
563 Ehrenreich H, Timner W, Sirén AL. A novel role for an established player: anemia drug erythropoietin for the treatment of cerebral hypoxia/ischemia. *Transfus Apher Sci* 2004;**31:**39-44.
564 Pajonk FG, Wobrock T, Gruber O, Scherk H, Berner D, Kaizl I, *et al.* Hippocampal plasticity in response to exercise in schizophrenia. *Arch Gen Psychiatry* 2010;**67:**133-143.
565 Vaynman S, Ying Z, Gomez-Pinilla F. Hippocampal BDNF mediates the efficacy of exercise on synaptic plasticity and cognition. *Eur J Neurosci* 2004;**20:**2580-2590.
566 Pereira AC, Huddleston DE, Brickman AM, Sosunov AA, Hen R, McKhann GM, *et al.* An in vivo correlate of exercise-induced neurogenesis in the adult dentate gyrus. *Proc Natl Acad Sci U S A* 2007;**104:**5638-5643.

567 Rathbone J, Zhang L, Zhang M, Xia J, Liu X, Yang Y, *et al.* Chinese herbal medicine for schizophrenia: cochrane systematic review of randomised trials. *Br J Psychiatry* 2007;**190:**379-384.
568 Singh V, Singh SP, Chan K. Review and meta-analysis of usage of ginkgo as an adjunct therapy in chronic schizophrenia. *Int J Neuropsychopharmacol* 2010;**13:**257-271.
569 Krieglstein J, Beck T, Seibert A. Influence of an extract of Ginkgo biloba on cerebral blood flow and metabolism. *Life Sci* 1986;**39:**2327-2334.
570 Mashayekh A, Pham DL, Yousem DM, Dizon M, Barker PB, Lin DD. Effects of Ginkgo biloba on cerebral blood flow assessed by quantitative MR perfusion imaging: a pilot study. *Neuroradiology* 2011;**53:**185-191.
571 Lee EJ, Chen HY, Wu TS, Chen TY, Ayoub IA, Maynard KI. Acute administration of Ginkgo biloba extract (EGb 761) affords neuroprotection against permanent and transient focal cerebral ischemia in Sprague-Dawley rats. *J Neurosci Res* 2002;**68:**636-645.
572 Fucetola R, Newcomer JW, Craft S, Melson AK. Age- and dose-dependent glucose-induced increases in memory and attention in schizophrenia. *Psychiatry Res* 1999;**88:**1-13.
573 Stone WS, Thermenos HW, Tarbox SI, Poldrack RA, Seidman LJ. Medial temporal and prefrontal lobe activation during verbal encoding following glucose ingestion in schizophrenia: A pilot fMRI study. *Neurobiol Learn Mem* 2005;**83:**54-64.
574: Sokoloff L. Circulation and energy metabolism of the brain. In Siegel G, Agranoff B, Albers RW, Molinoff P (eds) *Basic neurochemistry: molecular, cellular, and medical aspects.* Raven Press, New York1989,
575Kalinowsky LB, Hippius H, Klein HE. *Biological Treatments in Psychiatry* New York, 1982: Grune & Stratton
576.Crammer, J. L. Insulin coma therapy for schizophrenia. *J R Soc Med* **93,** 332-3 (2000).
577 Katakam PV, Domoki F, Lenti L, Gáspár T, Institoris A, Snipes JA, *et al.* Cerebrovascular responses to insulin in rats. *J Cereb Blood Flow Metab* 2009;**29:**1955-1967.
578 Sanderson TH, Kumar R, Murariu-Dobrin AC, Page AB, Krause GS, Sullivan JM. Insulin activates the PI3K-Akt survival pathway in vulnerable neurons following global brain ischemia. *Neurol Res* 2009;**31:**947-958.
579 Tallroth G, Ryding E, Agardh CD. Regional cerebral blood flow in normal man during insulin-induced hypoglycemia and in the recovery period following glucose infusion. *Metabolism* 1992;**41:**717-721.
580 Olincy A, Harris JG, Johnson LL, Pender V, Kongs S, Allensworth D, *et al.* Proof-of-concept trial of an alpha7 nicotinic agonist in schizophrenia. *Arch Gen Psychiatry* 2006;**63:**630-638.
581 Freedman R, Olincy A, Buchanan RW, Harris JG, Gold JM, Johnson L, *et al.* Initial phase 2 trial of a nicotinic agonist in schizophrenia. *Am J Psychiatry* 2008;**165:**1040-1047.
582 Uchida S, Hotta H, Misawa H, Kawashima K. Sustained subcutaneous infusion of nicotine enhances cholinergic vasodilation in the cerebral cortex induced by stimulation of the nucleus basalis of Meynert in rats. *Eur J Pharmacol* 2011;**654:**235-240.
583 Kinross-Wright V. CHLORPROMAZINE AND RESERPINE IN THE TREATMENT OF PSYCHOSES *Annals of the New York Academy of Sciences* 1955;**61:**174-182.
584 Bertler A. Effect of Reserpine on the Storage of Catechol Amines in Brain and Other Tissues *Acta Physiologica Scandinavica* 1961;**51:**75-83.
585 Shamon SD, Perez MI. Blood pressure lowering efficacy of reserpine for primary hypertension. *Cochrane Database Syst Rev* 2009;CD007655.
586 Martín JL. Review: slow repetitive transcranial magnetic stimulation reduces auditory hallucinations in people with schizophrenia. *Evid Based Ment Health* 2007;**10:**114.
587 Fitzgerald PB, Daskalakis ZJ. A review of repetitive transcranial magnetic stimulation use in the treatment of schizophrenia. *Can J Psychiatry* 2008;**53:**567-576.

588 Takano B, Drzezga A, Peller M, Sax I, Schwaiger M, Lee L, *et al.* Short-term modulation of regional excitability and blood flow in human motor cortex following rapid-rate transcranial magnetic stimulation. *Neuroimage* 2004;**23:**849-859.
589 Ohnishi T, Matsuda H, Imabayashi E, Okabe S, Takano H, Arai N, *et al.* rCBF changes elicited by rTMS over DLPFC in humans. *Suppl Clin Neurophysiol* 2004;**57:**715-720.
590 Knoch D, Treyer V, Regard M, Müri RM, Buck A, Weber B. Lateralized and frequency-dependent effects of prefrontal rTMS on regional cerebral blood flow. *Neuroimage* 2006;**31:**641-648.
591 Crossley NA, Constante M, McGuire P, Power P. Efficacy of atypical v. typical antipsychotics in the treatment of early psychosis: meta-analysis. *Br J Psychiatry* 2010;**196:**434-439.
592 Howes OD, Egerton A, Allan V, McGuire P, Stokes P, Kapur S. Mechanisms underlying psychosis and antipsychotic treatment response in schizophrenia: insights from PET and SPECT imaging. *Curr Pharm Des* 2009;**15:**2550-2559.
593 Cohen RM, Semple WE, Gross M, Nordahl TE, Holcomb HH, Dowling MS, *et al.* The effect of neuroleptics on dysfunction in a prefrontal substrate of sustained attention in schizophrenia. *Life Sci* 1988;**43:**1141-1150.
594 Di Filippo M, Tozzi A, Costa C, Belcastro V, Tantucci M, Picconi B, *et al.* Plasticity and repair in the post-ischemic brain. *Neuropharmacology* 2008;**55:**353-362.
595 Braun SM, Jessberger S. Adult neurogenesis and its role in neuropsychiatric disease, brain repair and normal brain function. *Neuropathol Appl Neurobiol* 2014;**40:**3-12.
596 Crayton JW, Meltzer HY. Motor endplate alterations in schizophrenic patients. *Nature* 1976;**264:**658-659.
597 Wakade CG, Mahadik SP, Waller JL, Chiu FC. Atypical neuroleptics stimulate neurogenesis in adult rat brain. *J Neurosci Res* 2002;**69:**72-79.
598 Shingo T, Gregg C, Enwere E, Fujikawa H, Hassam R, Geary C, *et al.* Pregnancy-stimulated neurogenesis in the adult female forebrain mediated by prolactin. *Science* 2003;**299:**117-120.
599 Garver DL, Holcomb JA, Christensen JD. Compromised myelin integrity during psychosis with repair during remission in drug-responding schizophrenia. *Int J Neuropsychopharmacol* 2008;**11:**49-61.
600 Wolf SA, Melnik A, Kempermann G. Physical exercise increases adult neurogenesis and telomerase activity, and improves behavioral deficits in a mouse model of schizophrenia. *Brain Behav Immun* 2011;**25:**971-980.
601 Malchow B, Keller K, Hasan A, Dörfler S, Schneider-Axmann T, Hillmer-Vogel U, *et al.* Effects of Endurance Training Combined With Cognitive Remediation on Everyday Functioning, Symptoms, and Cognition in Multiepisode Schizophrenia Patients. *Schizophr Bull* 2015;
602 Horwood JM, Dufour F, Laroche S, Davis S. Signalling mechanisms mediated by the phosphoinositide 3-kinase/Akt cascade in synaptic plasticity and memory in the rat. *Eur J Neurosci* 2006;**23:**3375-3384.
603 Peltier J, O'Neill A, Schaffer DV. PI3K/Akt and CREB regulate adult neural hippocampal progenitor proliferation and differentiation. *Dev Neurobiol* 2007;**67:**1348-1361.
604 Reif A, Fritzen S, Finger M, Strobel A, Lauer M, Schmitt A, *et al.* Neural stem cell proliferation is decreased in schizophrenia, but not in depression. *Mol Psychiatry* 2006;**11:**514-522.
605 Thagard PR. The Best Explanation: Criteria for Theory Choice *Journal of Philosophy* 1978;**75:**76-92.
606 Schmidt-Kastner R, van Os J, Esquivel G, Steinbusch HW, Rutten BP. An environmental analysis of genes associated with schizophrenia: hypoxia and vascular factors as interacting elements in the neurodevelopmental model. *Mol Psychiatry* 2012;**17:**1194-1205.

607 Green MF, Satz P, Christenson C. Minor physical anomalies in schizophrenia patients, bipolar patients, and their siblings. *Schizophr Bull* 1994;**20:**433-440.
608 Lieberman JA. Is schizophrenia a neurodegenerative disorder? A clinical and neurobiological perspective. *Biol Psychiatry* 1999;**46:**729-739.
609 Isohanni I, Järvelin MR, Jones P, Jokelainen J, Isohanni M. Can excellent school performance be a precursor of schizophrenia? A 28-year follow-up in the Northern Finland 1966 birth cohort. *Acta Psychiatr Scand* 1999;**100:**17-26.
610 Tiihonen J, Haukka J, Henriksson M, Cannon M, Kieseppä T, Laaksonen I, *et al.* Premorbid intellectual functioning in bipolar disorder and schizophrenia: results from a cohort study of male conscripts. *Am J Psychiatry* 2005;**162:**1904-1910.
611 Khandaker GM, Barnett JH, White IR, Jones PB. A quantitative meta-analysis of population-based studies of premorbid intelligence and schizophrenia. *Schizophr Res* 2011;
612 Černis E, Vassos E, Brébion G, McKenna PJ, Murray RM, David AS, *et al.* Schizophrenia patients with high intelligence: A clinically distinct sub-type of schizophrenia? *Eur Psychiatry* 2015;
613 Howard R, Jeste D. Late-onset schizophrenia. In Weinberger DR, Harrison PJ (eds) *Schizophrenia.* Wiley-Blackwell, Chichester, 2011, pp 47-61.
614 Hulshoff Pol HE, Kahn RS. What happens after the first episode? A review of progressive brain changes in chronically ill patients with schizophrenia. *Schizophr Bull* 2008;**34:**354-366.
615 Rabinowitz J, Levine SZ, Haim R, Häfner H. The course of schizophrenia: progressive deterioration, amelioration or both? *Schizophr Res* 2007;**91:**254-258.
